# Genetic basis for broad interspecific compatibility in *Solanum verrucosum*

**DOI:** 10.1111/tpj.70426

**Published:** 2025-08-21

**Authors:** William Behling, Joseph Coombs, Thilanka Ranaweera, Brieanne Vaillancourt, John P. Hamilton, Julia Brose, C. Robin Buell, David S. Douches

**Affiliations:** ^1^ Department of Plant Soil and Microbial Sciences, Molecular Plant Science Michigan State University 1066 Bogue Street Room 2210 East Lansing Michigan 48824 USA; ^2^ Department of Plant Biology, Plant Biology Laboratories Michigan State University 612 Wilson Rd Room 166 East Lansing Michigan 48824 USA; ^3^ Department of Energy (DOE) Great Lakes Bioenergy Research Center Michigan State University East Lansing Michigan 48824 USA; ^4^ Center for Applied Genetic Technologies University of Georgia 111 Riverbend Rd. Room 249 Athens Georgia 30602 USA; ^5^ Department of Crop & Soil Sciences University of Georgia 111 Riverbend Rd. Room 249 Athens Georgia 30602 USA; ^6^ Institute of Plant Breeding, Genetics, & Genomics University of Georgia 111 Riverbend Rd. Room 249 Athens Georgia 30602 USA; ^7^ The Plant Center University of Georgia 111 Riverbend Rd. Room 249 Athens Georgia 30602 USA

**Keywords:** interspecific compatibility, unilateral incompatibility, gametophytic self‐incompatibility, potato, *Solanum verrucosum*, *Solanum*

## Abstract

*Solanum verrucosum* Schlechtendal (2*x* = 2*n* = 24) is unique among the clade 4 *Solanum* Sect *Petota* species. In addition to being one of the only fully self‐compatible diploid potato species, *S. verrucosum* is the only clade 4 species that lacks prezygotic interspecific reproductive barriers. This allows *S. verrucosum* to accept pollen from a broad range of *Solanum* species and thereby serving as a genetic “bridge” between the cultivated or primary potato gene pool and distantly related wild relatives in the tertiary gene pool. The genetic mechanisms underlying self‐compatibility in *Solanum* often underpin interspecific compatibility interactions, which in *S. verrucosum*, has been attributed to the lack of *S‐RNase* expression. Using an interspecific F2 mapping population (*n* = 150), we investigated the genetic mechanisms responsible for the lack of interspecific reproductive barriers in *S. verrucosum*. This F2 population was evaluated for the ability to accept pollen from two clade 1, 1 EBN species (*S. pinnatisectum* and *S. tarnii*); from which two QTL for interspecific compatibility were identified on chromosomes 1 and 11, explaining 56.6% of the phenotypic variation observed. To identify the genetic basis of interspecific compatibility, we generated a chromosome‐scale genome assembly of *S. verrucosum* MSII1813‐2 and performed gene expression profiling of reproductive organs. Differential gene expression of *S‐RNase*, located within the chromosome 1 QTL, confirmed the central role of the *S*‐locus and specifically, *S‐RNase*, in interspecific compatibility. Discovery of a non‐*S*‐locus QTL is consistent with previous findings that other non‐*S*‐locus factors are necessary for interspecific compatibility in *S. verrucosum*.

## Significance Statement

Identifying the genetic components that contribute to the lack of prezygotic interspecific reproductive barriers in *Solanum verrucosum* will allow for more efficient utilization of crop wild relatives for the genetic improvement of potato. This is the first conclusive evidence of a non‐*S*‐locus factor on chromosome 11 being involved in the interspecific reproductive barriers of *Solanum*.

## INTRODUCTION

The evolutionary history of *Solanum verrucosum* Schlechtendal (2*x* = 2*n* = 24) is an enigma. Despite its native range in north and central Mexico, its closest living relatives are members of the *S. brevicaule* species complex native to central Bolivia (Huang et al., 2019; Spooner et al., 2004; Yan et al., 2023). *S. verrucosum* is also unusual as it is one of the only diploid potato species that is fully self‐compatible (SC) (Abdalla & Hermsen, 1973; Cipar et al., 1964). In *Solanum*, there is a significant overlap between genetic factors that govern gametophytic self‐incompatibility and those that mediate interspecific compatibility (Baek et al., 2015; Tovar‐Méndez et al., 2014, 2017). This correlation is particularly striking in *S. verrucosum*, which is highly SC, self‐fertile, and able to accept pollen from a broad range of wild *Solanum* species (Behling et al., 2024). This allows *S. verrucosum* to serve as a genetic “bridge” between the cultivated or primary potato gene pool and the distantly related species in the tertiary gene pool (Behling & Douches, 2023; Bethke et al., 2017; Jansky & Hamernik, 2009). In recent years, utilizing *S. verrucosum* to access the diploid wild potato species in the tertiary gene pool has gained more interest; since as a group, these species are exceptionally rich in novel and valuable disease and pest resistance traits (Bethke et al., 2017; Jansky & Hamernik, 2009). The first to document the ability of *S. verrucosum* to hybridize with these sexually isolated species were Hermsen and Ramanna (1976) when they made interspecific hybrids with *S. bulbocastanum*. Later work by multiple researchers demonstrated that *S. verrucosum* is capable of creating bridge hybrids with a broad range of wild species, bypassing the interspecific reproductive barriers (IRBs) that isolate distantly related species from cultivated germplasm (Bamberg et al., 2021; Behling et al., 2024; Dinu et al., 2005; Jansky & Hamernik, 2009; Yermishin et al., 2014).

In *S. verrucosum*, self‐compatibility has been attributed to the lack of *S*‐ribonuclease (*S‐RNase*) expression in the style (Behling, 2022; Eijlander et al., 2000). *S‐RNase* plays a central role in the gametophytic self‐incompatibility (GSI) system in *Solanum* species (Kubo et al., 2010). The GSI system is controlled by a single multiallelic locus, called the *S*‐locus (Kubo et al., 2010). This locus contains two major factors, pistil‐expressed *S‐RNase* and a suite of pollen‐expressed *S*‐locus F‐box (SLF) proteins (Kubo et al., 2010; Mcclure, 2006). S‐RNase in conjunction with other factors such as HT exerts a cytotoxic effect on incompatible pollen tubes penetrating the style (Goldraij et al., 2006; Lee et al., 2023). In compatible non‐self‐pollinations, SLF proteins along with other members of the SCF complex initiate the degradation of non‐self *S‐RNase* allowing pollen tubes to grow unimpeded (Goldraij et al., 2006; Kubo et al., 2010). The lack of *S‐RNase* expression in *S. verrucosum* would, in theory, remove a central barrier to self and interspecific pollen.

While *S‐RNase* plays a central role in self‐incompatibility in *Solanum*, it is not the only factor in interspecific compatibility. In the tomato clade, it has been demonstrated that S‐RNase alone cannot reject incompatible pollen tubes, and a functional allele of *HT‐A* or *HT‐B* must also be present (Tovar‐Méndez et al., 2014). Additionally, HT was found to mediate S‐RNase‐independent pollen rejection in interspecific pollinations in tomato (Tovar‐Méndez et al., 2017). The overlap between GSI and IRBs is complex and highly dependent on the parents involved (Moreels et al., 2023; Murfett et al., 1996). For example, in interspecific crosses between the wild and cultivated tomato species *S. pennellii* and *S. lycopersicon*, the additional factors ornithine decarboxylase (ODC2) and defective in induced resistance 1‐like (SpDIR1L) play significant roles in interspecific pollen rejection (Muñoz‐Sanz et al., 2021; Qin & Chetelat, 2021). Given the close relationship between the potato and tomato clades, it would be reasonable to conclude that other factors such as HT, ODC2, and DIR1L. may also play a role in interspecific compatibility in potato. This is supported by previous research in which CRISPR‐Cas9 knockouts of *S‐RNase* in *S. tuberosum* could not replicate the phenotype observed in *S. verrucosum*, demonstrating the role of additional factors (Behling & Douches, 2023).

**Figure 1:**
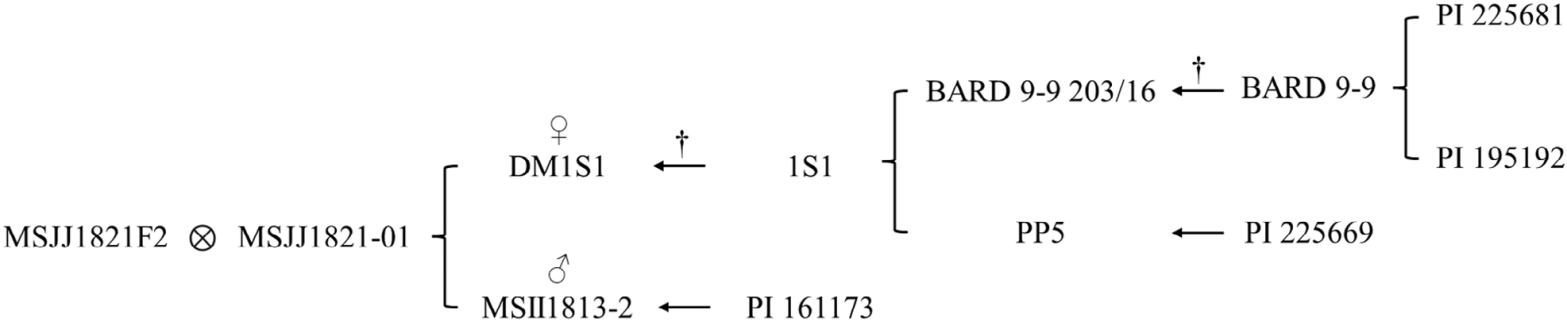
Pedigree of mapping population and its parents. MSII1813‐2 and PP5 are clonal selections derived from their source accessions. ^†^Represents the generation of doubled monoploids via anther culture and spontaneous chromosome doubling during regeneration in tissue culture (Paz & Veilleux, 1999).

To identify the genetic basis of broad interspecific compatibility in *S. verrucosum*, we created an F2 mapping population (*n* = 150) segregating for interspecific reproductive barriers. The parental clones DM1S1 and MSII1813‐2 were selected as parents. The F1 and the resulting F2 population were designated MSJJ1821 (Figure 1). The female parent DM1S1 is a *S. tuberosum* Group Phureja doubled monoploid that is effectively male sterile and has functional prezygotic IRBs (Figure S1) (Jayakody et al., 2023). The male parent MSJJ1813‐2 is a *S. verrucosum* selection made from PI 161173 and exhibits a high degree of male fertility, self‐compatibility, and lacks prezygotic IRBs (Behling et al., 2024). The population was evaluated for the ability to accept pollen from the two clade 1, 1 endosperm balance number (1 EBN) species *S. pinnatisectum* and *S. tarnii*, and QTL analysis was used to identify loci associated with reproductive traits (Johnston et al., 1980). An additional replicated experiment explored the variation in post‐zygotic hybridization barriers in five F2 progeny that expressed the parental *S. verrucosum* interspecific compatibility phenotype. This replicated experiment used pollen from the 1 EBN species *S. commersonii* and *S. tarnii*, which typically have strong interspecific reproductive barriers with *S. tuberosum* (Behling & Douches, 2023). To further dissect the molecular basis of interspecific compatibility in *S. verrucosum*, we generated a genome assembly for MSII1813‐2 and performed gene expression profiling in reproductive organs to identify genes within significant QTL associated with interspecific compatibility.

## RESULTS AND DISCUSSION

### F2 phenotyping

**Figure 2:**
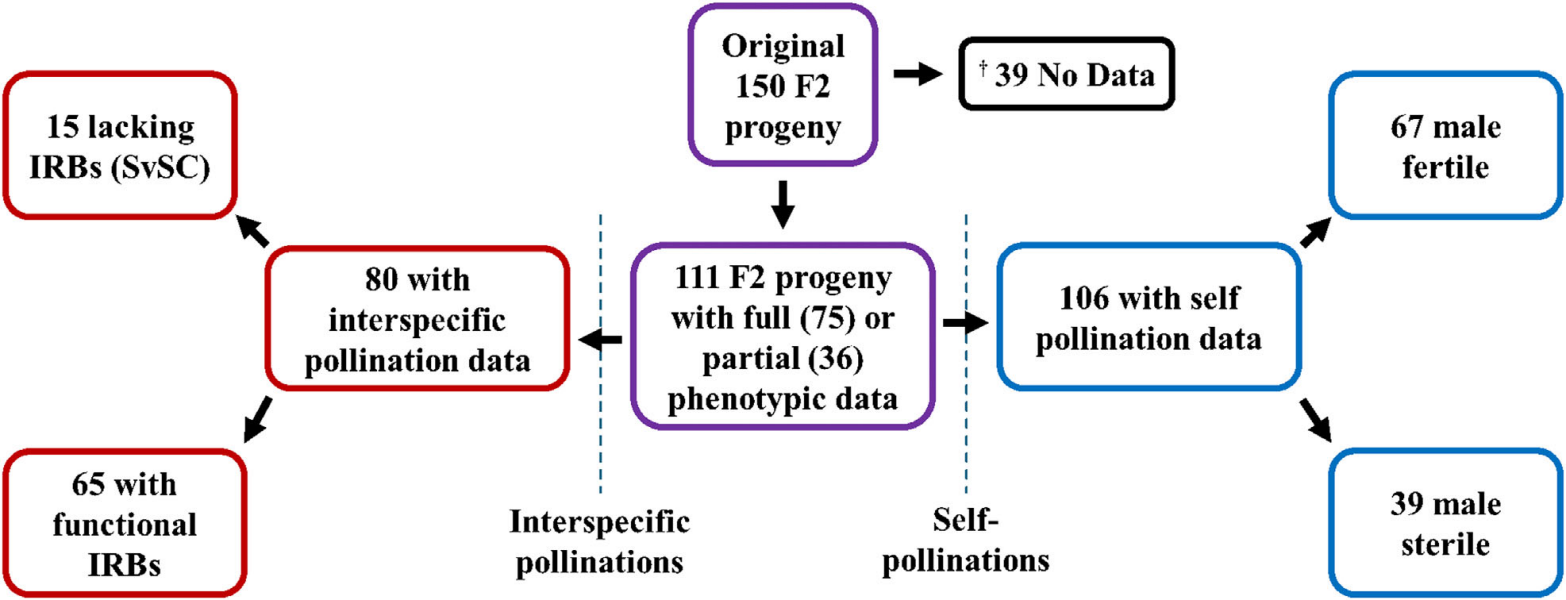
Pipeline for phenotyping the F2 population. IRBs, interspecific reproductive barriers; SvSC, the parental *S. verrucosum* self‐compatibility phenotype that also lacks IRBs. ^†^Individuals that failed to flower, died during the course of the experiment, or lacked sufficient pollen stainability.

The F2 population was phenotyped over a period of 48 days for interspecific compatibility and male fertility using self‐pollinations, pollen viability assays, and interspecific pollinations with *S. pinnatisectum* and *S. tarnii* pollen donors. Of the original 150 F2 progeny, full self‐ and interspecific pollination data could only be collected for 75 individuals (Table S1). Of the remaining 75 individuals, partial phenotype data (either interspecific or self‐pollination data) were obtained for 36 individuals, while the remaining 39 individuals failed to flower, died during the course of the experiment, or lacked sufficient pollen stainability and thus were excluded from QTL analyses. From the 111 F2 individuals with full or partial pollination data, we initially classified them into different phenotypic classes based on their behavior in self‐ and interspecific crosses as we hypothesized that self‐compatibility could be segregating. It later became apparent after further examination of pollen phenotypes using fluorescent microscopy and in vitro pollen germination assays that self‐compatibility was not segregating, but male fertility was (Behling, 2022; Lee, 2021). Therefore, F2s that had initially been classified as self‐incompatible (SI), failing to set fruit after repeated self‐pollination, were reclassified as male sterile. After completion of the phenotyping, two sets of phenotypic data, one for interspecific compatibility and one for male fertility, each with two phenotypic classes remained (Figure 2, Table 1).

**Table 1:** Summary of phenotyping results from self‐ and interspecific pollinations

	Lacking IRBs (SvSC)	Functional IRBs	Partial data	Total
Male fertile	13	48	6	67
Male sterile	2	12	25	39
Partial data	—	5	—	5
Total	15	65	31	111

In self‐pollination assays, 67 individuals produced fruit after self‐pollination, indicating that they were male fertile, representing our first phenotypic class. An additional 39 individuals did not set fruit after self‐pollination and were initially deemed SI and later reclassified to male sterile, representing our second phenotypic class (Figure 2, Table 1). Because all F2s had at least one *S. verrucosum S‐RNase* allele based on SNP data, all F2s would theoretically be SC, much like the F1, if they had been male fertile.

Interspecific pollinations were used to identify F2 progeny with the parental *S. verrucosum* interspecific compatibility phenotype (SvSC) which lacks IRBs. We utilized *S. pinnatisectum* and *S. tarnii* as pollen donors for these interspecific pollinations as pollen tubes from these species are inhibited in the styles of DM1S1 (Figure S1). After the completion of the phenotyping process, 15 F2 individuals were classified as having SvSC or lacking IRBs similar to the *S. verrucosum* parental phenotype, and 65 individuals were classified as having functional IRBs (Figure 2, Table 1).

### SNP genotyping and linkage map construction

Single nucleotide polymorphism (SNP) genotyping was performed using total genomic DNA on the Illumina Infinium SolCAP V4 30K Potato SNP Array (Endelman et al., 2024). The Illumina GenomeStudio v2.0.5 software was used for calling three cluster SNP genotypes with a diploid model. The genotype calls were filtered to exclude SNPs categorized as poor quality markers and missing data resulting in a total of 7322 quality‐filtered SNPs for further analysis. After removing non‐segregating SNPs, co‐segregating SNPs, and SNPs mapped to chr00 on the DM v4.03 pseudomolecule, a final set of 1937 segregating SNPs was used to construct the linkage map. Linkage groups for all chromosomes were well defined based on the SNP positions in the DM1‐3516 v4.03 genome assembly (Table 2). The linkage map spanned a genetic distance of 890.6 cM. Of the 1937 mapped SNPs, 1492 markers mapped to unique linkage group positions, and 445 were mapped to co‐segregating positions. The distance between markers varied from 0.1 to 8.4 cM, with an average of 0.6 cM between SNPs.

**Table 2:** Summary of SNP marker information from mapping population linkage map

LG	Physical length DM1S1 v1 (Mb)	Physical length MSII1813‐2 v1 (Mb)	Physical length DM 1‐3 516 v4.03 (Mb)	Map length (cM)	Number of mapped SNPs	Average interloci distance (cM)	Interloci distance range (cM)
1	88.2	90.4	88.7	109.4	201	0.5	0.3–8.4
2	47.2	46.8	48.6	62.4	181	0.3	0.3–1.4
3	60.5	59.8	62.3	80.7	180	0.5	0.3–2.8
4	71.1	68.7	72.2	82.2	180	0.5	0.2–2.8
5	54.9	50.3	52.1	78.1	139	0.6	0.3–6.7
6	60.6	52.2	59.5	75.4	189	0.4	0.3–4.0
7	56.3	54.3	56.8	63.2	156	0.4	0.3–1.7
8	58.3	56.4	56.9	67.8	188	0.4	0.3–3.9
9	69.1	60.3	61.5	82.9	169	0.5	0.3–3.9
10	60.1	57.1	59.8	67.8	115	0.6	0.2–7.5
11	47.9	43.5	45.5	57.2	107	0.5	0.1–2.1
12	59.0	58.9	61.2	63.5	132	0.5	0.3–2.5
Total	733.1	698.7	725.0	890.6	1937	N/A	0.1–8.4

### QTL analysis—Identification of QTLs for interspecific compatibility

**Figure 3:**
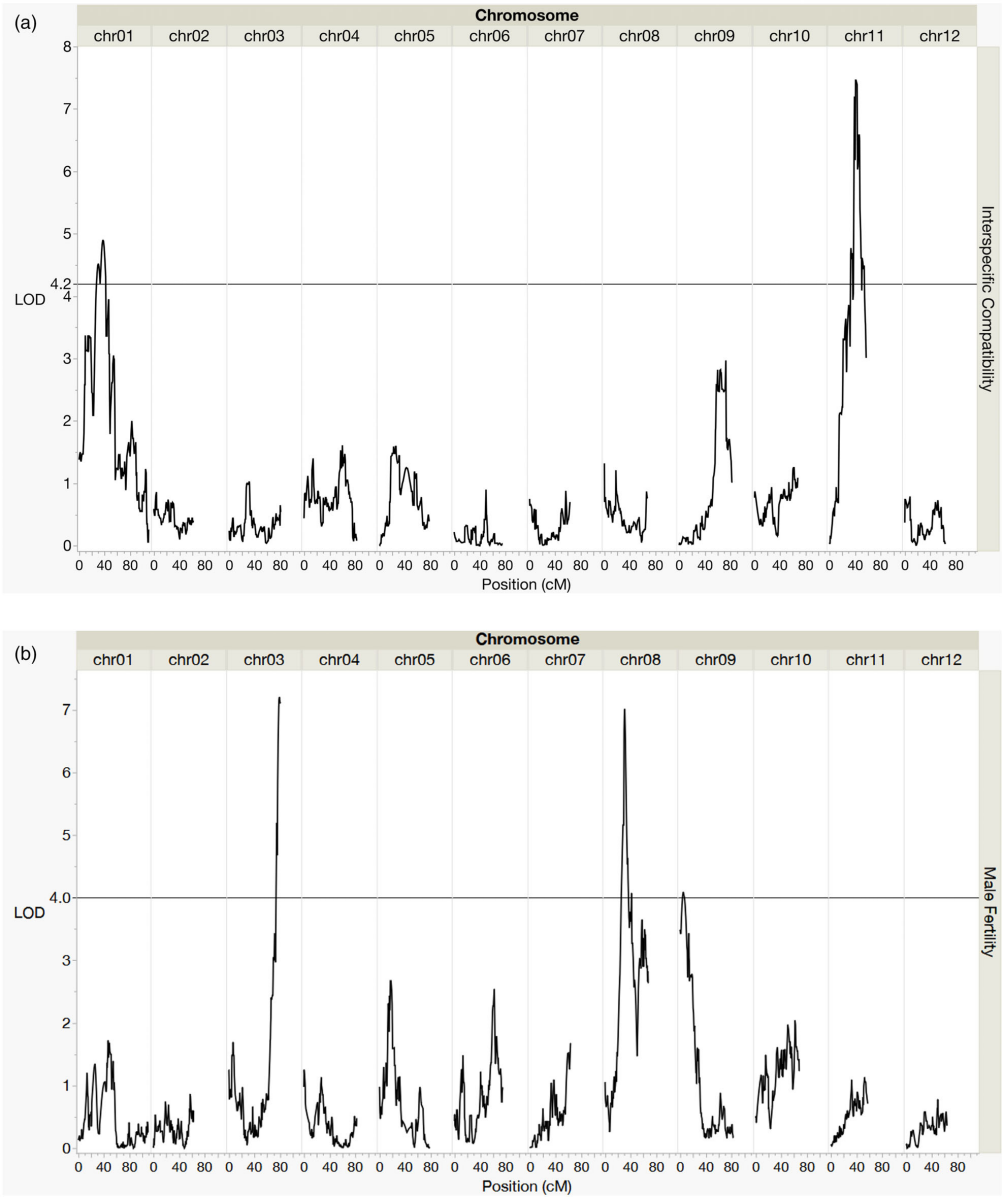
LOD score distribution for interspecific compatibility and male fertility QTLs across the 12 potato chromosomes. Fruit development after interspecific and self‐pollination was used as proxies for interspecific compatibility (a) and male fertility (b), respectively.

For QTL analysis, the multiple QTL model (MQM) was used in MapQTL 6 (Van Ooijen, 2009) resulting in two significant QTL regions associated with interspecific compatibility: the first QTL encompassing five SNPs between 26.3 and 42.2 cM on chromosome 1 and the second between 33.2 and 54.4 cM on chromosome 11 (Figure 3, Table 3, and Table S2). The 15.9 cM QTL interval on chromosome 1 encompasses the centromeric region according to analyses from Hosaka et al. (2022) as well as the *S*‐locus given the location of *S‐RNase* and *SLF* positions in the parental genome assemblies (Figures S2 and S3, Tables S3 and S4) (Jayakody et al., 2023). The presence of two QTLs for interspecific compatibility (chromosomes 1 and 11) agrees with previous findings that found the lack of *S‐RNase* expression cannot replicate the complete absence of prezygotic IRBs as observed in *S. verrucosum* (Behling & Douches, 2023). The second QTL associated with interspecific compatibility on chromosome 11 is novel to this population. No factors associated with GSI or prezygotic IRBs have been previously identified on chromosome 11 in *Solanum*. More work is necessary to find the exact identity of this pistil‐expressed unilateral incompatibility factor (denoted hereinafter *ui11.1*) located in the chromosome 11 QTL. It seems that *ui11.1* has an epistatic relationship with *S‐RNase* on chromosome 1, as F2 progeny lacking IRBs are significantly more likely to be homozygous for the *S. verrucosum* alleles of *S‐RNase* and *ui11.1* than any other genotype combination (Fisher's exact test *P* < 0.0001) (Figure S4).

**Table 3:** Significant SNPs with the highest LOD scores from each QTL

SNP	Chromosome	Position DM1‐3 v4.03 (Mb)	Position DM1‐3 v6.1 (Mb)	Interval map position (cM)	LOD	LOD significance threshold	% Phenotypic variation explained
Interspecific compatibility							
solcap_snp_c1_8906	chr01	13.7	13.0	26.3	4.0	4.2	19.9
	*chr01*	*0.0*	*Centromere 31–34*	*30.3*	*4.5*	*4.2*	*22.4*
	*chr01*	*0.0*	*S‐locus* *35–47*	*31.3*	*4.5*	*4.2*	*22.2*
solcap_snp_c2_54811	chr01	46.3	53.0	33.0	4.2	4.2	21.0
PotVar0095207	chr01	45.8	53.4	33.0	4.2	4.2	21.0
ST4.03ch01_45174658	chr01	45.2	54.0	33.4	4.2	4.2	21.0
solcap_snp_c2_2653	chr01	58.7	60.4	41.8	4.3	4.2	21.5
ST4.03ch11_40111293	chr11	40.1	41.7	40.7	7.5	4.2	34.2
PotVar0112839	chr11	40.5	42.0	41.1	7.5	4.2	34.2
PotVar0112395	chr11	40.6	42.1	41.4	7.5	4.2	34.2
Male fertility							
ST4.03ch03_61342320	chr03	61.3	59.7	79.0	7.2	4.0	27.1
ST4.03ch03_61233807	chr03	61.2	59.6	79.0	7.2	4.0	27.1
PotVar0020829	chr03	61.2	59.6	79.0	7.2	4.0	27.1
solcap_snp_c2_51053	chr08	46.4	48.7	30.8	7.0	4.0	26.5
ST4.03ch09_585190	chr09	0.6	0.6	6.0	4.0	4.0	16.2

The italics show the location of the centromeric region and S‐locus. There are no SNPs associated with these regions.

### QTL analysis—Additional QTLs discovered for male fertility

Due to the segregation of male fertility in the F2 population, three additional significant QTLs were discovered that are associated with male fertility: 74.9–80.7 cM on chromosome 3, 25.3–41.4 cM on chromosome 8, and a single SNP at 6 cM on chromosome 9 (Figure 3, Table 3, Table S2). Together, these QTL explain 69.8% of the phenotypic variation observed between F2 progeny for fruit development following self‐pollination. We initially assumed that SC may segregate in this population, yet later determined that self‐pollen failed to germinate on the styles of presumed “self‐incompatible” individuals, indicating that SC was not segregating, but male fertility was.

On chromosome 3, the SNP at the QTL peak PotVar0020829 is located 31.3 kb upstream of a Solver.v1.03_VERG035240, which encodes a phospholipid:diacylglycerol acyltransferase (PDAT) with reduced expression in the pollen of the male sterile F2, MSJJ1821F2‐041 (Table S5). In *Arabidopsis thaliana*, PDAT1 together with Acyl‐CoA:diacylglycerol acyltransferase 1 (DGAT1) serves essential functions in pollen and embryo development, with double mutants unable to produce viable pollen (Bai et al., 2023; Zhang et al., 2009). The protein sequences of the parental homologs for this PDAT are similar but not the identical (Figure S5). In this population, individuals with at least one DM1S1 allele at this locus were significantly more likely to be male fertile (Fisher's exact test *P* < 0.0001). This is also demonstrated in the F2 progeny used for RNA sequencing, as the male fertile F2 individuals MSJJ1821‐049 and MSJJ1821‐091 are, respectively, homozygous and heterozygous for the DM1S1 allele, while male sterile MSJJ1821‐041 is homozygous for the MSII1813‐2 (*S. verrucosum*) allele according to the SNP data.

**Figure 4:**
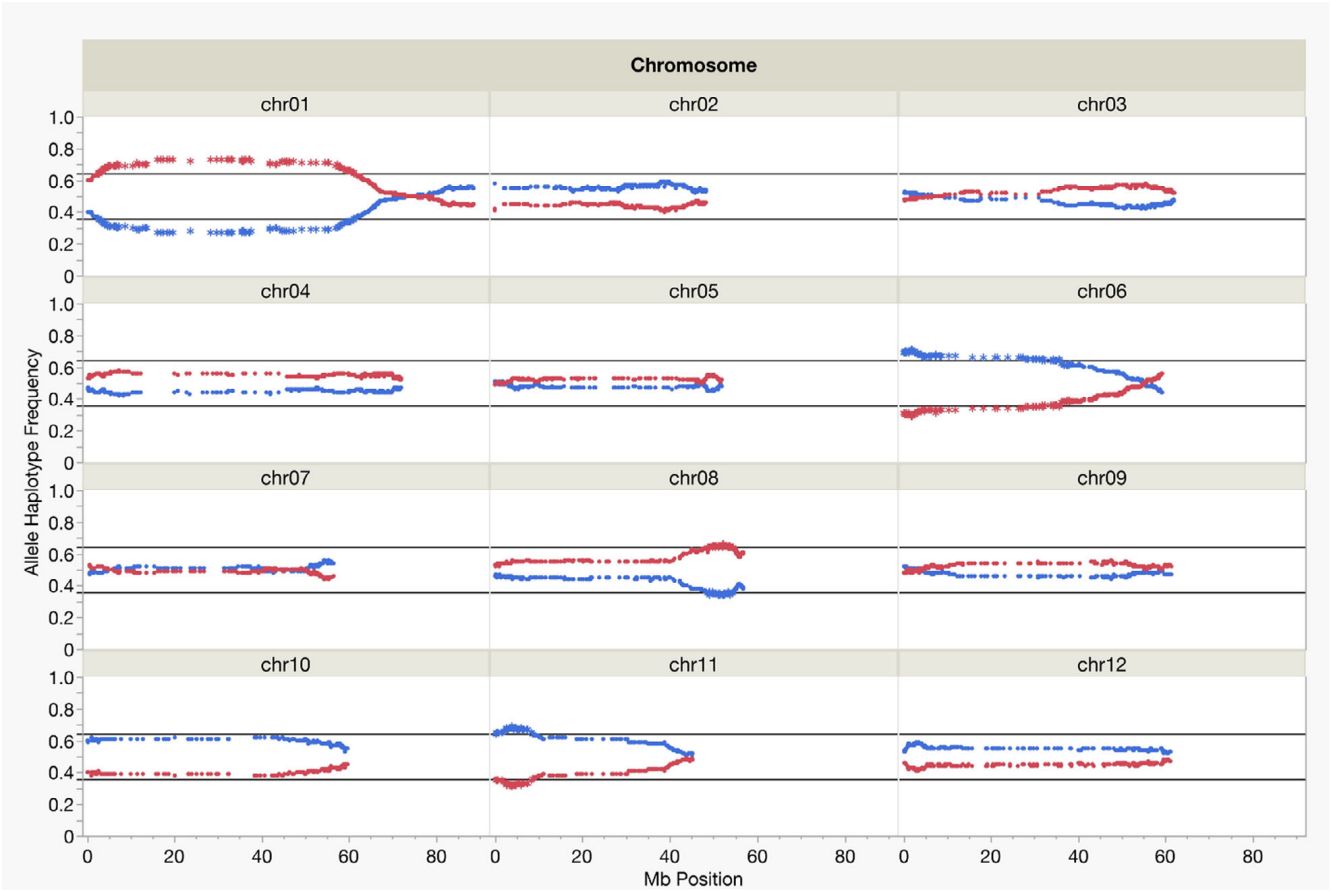
Distribution of segregation ratios of allele haplotypes plotted over physical distance (Mb) for each chromosome according to DM v4.03 assembly. Significance threshold (black line) was determined with *χ* ^2^ test and Bonferroni multiple test correction for the 6983 SNPs evaluated. *α* = 7.16 × 10^−6^. The DM1S1 haplotypes are plotted in blue and MSII1813‐2 haplotypes in red.

On chromosome 8, the SNP of greatest significance in the QTL associated with male fertility was solcap_snp_c2_51053 (Table 3). This SNP is in a UDP‐glycosyltransferase superfamily protein (UGT) (Soltu.DM.08G019750.1) and is upstream of several pectinacetylesterase family proteins (PAEs); both UGTs and PAEs are generally associated with pollen fertility. In petunia, UGT79B31 is necessary for flavonoid modification essential for pollen function growth, and it has been demonstrated in tobacco and potato that perturbations in PAE expression result in male sterility (Cankar et al., 2014; Gou et al., 2012; Knoch et al., 2018). All pollen‐expressed PAEs from MSII1813‐2 in this QTL region have low expression except for Solver.v1.08_VERG019120, which is sister to a clade of orthologous PAEs across *Solanum chacoense* M6 accession, *S. tuberosum* DM 1‐3 516 R44, *S. tuberosum* RH89‐039‐16, *S. tuberosum* DM1S1, *Solanum chacoense*, and an additional PAE in *S. verrucosum* (Figure S6). The PAEs in the same region in DM and DM1S1 (both male sterile) are sister to the additional lowly expressed PAE in *S. verrucosum*. Whereas the highly male fertile *S. chacoense* diverged most recently from Solver.v1.08_VERG019120, which could indicate a shared expression change across male sterile accessions after their divergence (Figure S6). Significant segregation distortion at this locus on chromosome 8 indicates that pollen carrying the DM1S1 allele may have impaired function leading to lower transmission of the DM1S1 allele (Figure 4). Additionally, the results of the Fisher's exact test (*P* < 0.0001) indicate a significant association between male sterility and being homozygous for the DM1S1 allele at the chromosome 8 QTL locus. This QTL may partially explain the general lack of male fertility in DM1S1 as well (Jayakody et al., 2023).

The final QTL associated with male fertility is on chromosome 9 and is represented by the SNP ST4.03ch09_585190 (0.6 Mb DM v4.03) which resides within one of the receptor lectin kinases (LecRKs) (Soltu.DM.09G000810.1) in this QTL region. In *A. thaliana*, tomato, and rice, regular expression of certain functional LecRKs is necessary for normal pollen development (Micol‐Ponce et al., 2022; Peng et al., 2020; Wan et al., 2008). In this QTL region, a multiple sequence alignment of pollen‐expressed Soltu.DM.09G000840, a Concanavalin A‐like lectin protein kinase family protein, shows a clear deletion in the MSII1813‐2 haplotype relative to DM and DM1S1 (Figure S7). In our population, the presence of at least one DM1S1 allele at this locus on chromosome 9 was significantly associated with male fertility, Fisher's exact test (*P* = 0.0002).

### Transmission ratio distortion

Significant transmission ratio distortion (TRD) was observed on chromosomes 1, 6, 8, and 11 (*α* = 7.16 × 10^−6^) (Figure 4). The largest deviation from expected ratios was on chromosome 1, which was expected due to the presence of the *S*‐locus and its proximity to the pericentromeric region. The region of TRD on chromosome 8 was co‐located with the QTL on chromosome 8 associated with male fertility (Figures 3 and 4, Table 3). No other regions with significant TRD, besides those on chromosomes 1 and 8, were associated with QTLs for IRBs or male fertility. On chromosome 11, the region with significant TRD and the QTL for *ui11.1* are located on opposite ends of the chromosome (Figures 3 and 4, Table 3).

### Genome assembly and annotation of *S. verrucosum* MSII1813‐2 genome

Using PacBio HiFi reads, we assembled the *S. verrucosum* MSII1813‐2 genome resulting in a final assembly of 738 577 088 bp in 505 scaffolds with an N50 length of 57.1 Mb, of which 94.8% was anchored to chromosomes (Table S6). Benchmarking Universal Single Copy Orthologs (BUSCO) analysis of the genome revealed 99.3% complete BUSCO orthologs, with 97.3% being single copy orthologs, indicative of a high‐quality genome assembly (Table S7). Repetitive sequences were identified, resulting in 67% of the genome annotated as repetitive, of which retroelements were the most abundant (34.7%) (Table S8). Protein coding sequences were annotated to facilitate the identification of genes associated with reproductive traits; a total of 48 510 working gene models were annotated (Table S9) with an overall complete BUSCO score of 93.5% (Table S7).

### RNA‐seq and differential expression analysis

The QTL region on chromosome 1 is consistent with the position of the *S*‐locus in potato, which is located between 35 and 47 Mb on the DM1‐3 v6.1 assembly. The closest segregating SNPs to the *S*‐locus are at 13 Mb and 52.9 Mb, between which sit the centromeric region (31–34 Mb on the DM1‐3 v6.1 assembly) and the *S*‐locus (Hosaka et al., 2022). QTL mapping in this region is particularly challenging due to the suppression of recombination in the pericentromeric region and *S*‐locus along with strong prezygotic gamete selection against DM1S1 *S*‐alleles due to GSI, which results in severe segregation distortion (Clot et al., 2024; Kubo et al., 2010). While the small size of the mapping population contributes to the inability to define the QTL region, even large populations see significant transmission distortion and virtually no recombination in this region of chromosome 1 (Clot et al., 2024). For these reasons, RNA‐seq and differential expression analysis were utilized to gain insight into this region on chromosome 1.

**Figure 5:**
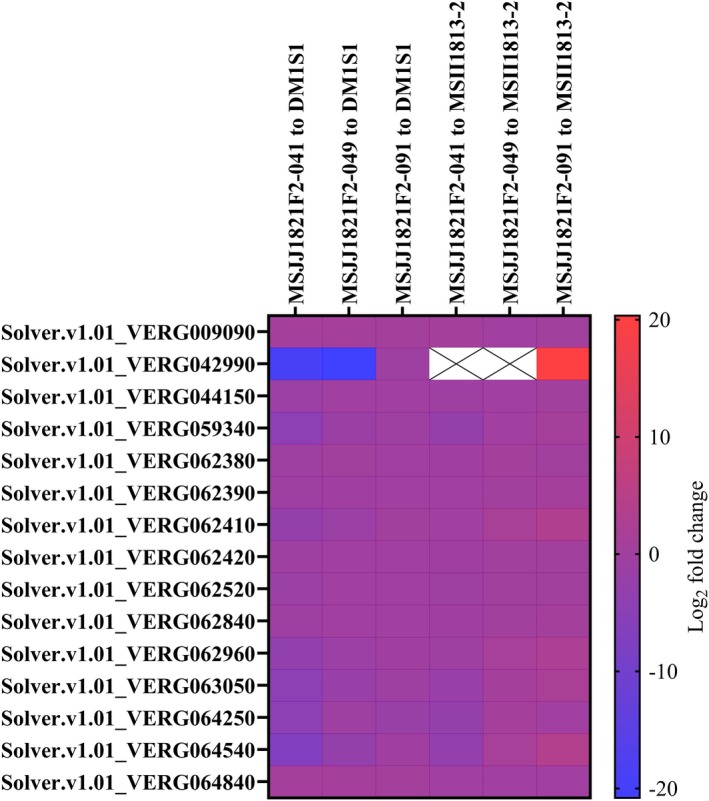
Heat map of Log_2_ fold change of significantly DE genes in the QTL region on chromosome 1. Stylar‐expressed *S‐RNase* is shown as Solver.v1.01_VERG042990. Comparisons between MSII1813‐2^†^ and the F2 progeny MSJJ1821F2‐041^†^ and MSJJ1821F2‐049^†^ have very low read counts and are not significantly differentially expressed between each other (adjusted *P* ≥ 0.05). The white cells represent genes with a base mean too low to calculate statistics in the DESeq2 package. ^†^Phenotypes of lines in comparisons: DM1S1 = male sterile with functional IRBs, MSII1813‐2 = male fertile and absence of IRBs (SvSC), MSJJ1821F2‐041 = male sterile with functional IRBs, MSJJ1821F2‐049 = male fertile and absence of IRBs (SvSC), and MSJJ1821F2‐091 = male fertile with functional IRBs.

Total RNA was isolated from styles and pollen of the parents of the F2 population and three F2 individuals representing the range of phenotypic variation observed in the F2 population: MSJJ1821F2‐041 = male sterile with functional IRBs, MSJJ1821F2‐049 = male fertile and absence of IRBs (SvSC), and MSJJ1821F2‐091 = male fertile with functional IRBs. For each plant, at least 30 styles and 100 μl of pollen were collected from newly opened flowers to remain consistent with phenotyping conditions. Sequencing was performed, yielding an average of 67.6 million for each sample. To identify significantly differentially expressed (DE) stylar genes between the F2 progeny being studied, each F2 was first compared to both parents and then genes were filtered by QTL region and significance. This resulted in 15 candidate genes on chromosome 1 and one on chromosome 11 that were DE in the styles of the F2 progeny and their parents, correlating with our observed phenotypes (Table S11). *S‐RNase* (Solver.v1.01_VERG042990) is located within the chromosome 1 QTL. Expression of *S‐RNase* was essentially absent in MSJJ1821F2‐041 and MSJJ1821F2‐049 compared to DM1S1, and *S‐RNase* was not significantly DE (adjusted *P* > 0.05) when compared with MSII1813‐2 (Figure 5). MSJJ1821F2‐091 had similar *S‐RNase* expression to DM1S1 and significantly higher expression than MSII1813‐2 (Figure 5). The significantly DE gene candidate on chromosome 11 in this analysis had no known molecular functions consistent with interspecific compatibility (Table S11). The F2 progeny MSJJ1821F2‐041 (functional IRBs) and MSJJ1821F2‐049 (nonfunctional IRBs) share their *S‐RNase* expression profiles with *S. verrucosum* while exhibiting different phenotypes (Figure 5). This demonstrates the importance of *ui11.1* in maintaining functional IRBs in the absence of *S‐RNase* as MSJJ1821F2‐041 is homozygous for the DM1S1 allele of *ui11.1* and MSJJ1821F2‐049 is homozygous for the MSII1813‐2 allele.

### Chromosome 11 QTL (ui11.1)

**Figure 6:**
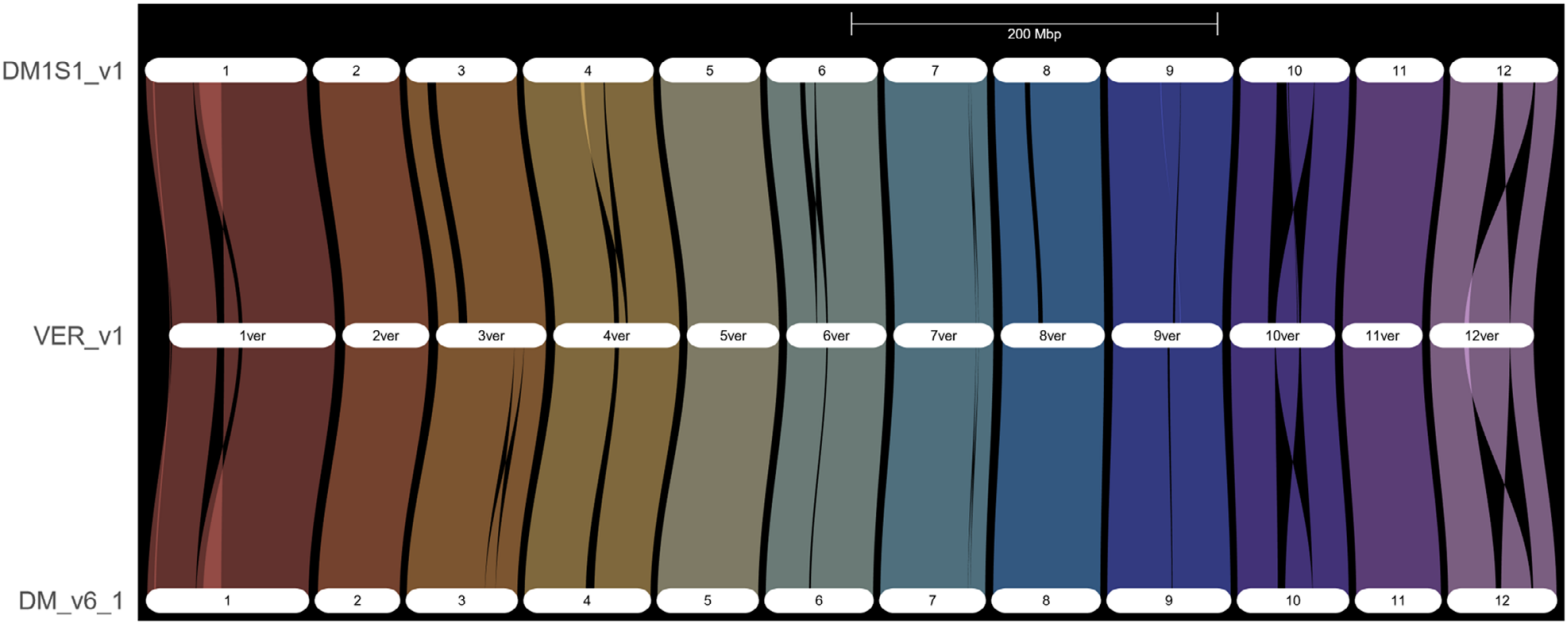
Riparian plot showing collinearity between the genomes of MSII1813‐2 (VER_v1), DM1S1, and DM 1‐3 516 R44 (DM_v6.1). Chromosomes scaled by physical position.

To understand genetic differences that underlie the chromosome 11 *ui11.1* QTL, we first identified syntenic blocks at the whole genome level between *S. verrucosum* (MSII1813‐2), DM1S1, and DM v6.1. In total, 302.46 Mb containing 66 100 genes was present in syntenic blocks between the three genomes reflecting a high degree of collinearity between these genomes apparent in the riparian plot (Data S1, Figure 6). With respect to structural variation between the two parents of the F2 population, 211.06 Mb (49 030 genes) was present as syntenic blocks with 17 250 genes in *S. verrucosum* not present in DM1S1 and 11 890 genes in DM1S1 not present in *S. verrucosum*.

**Figure 7:**
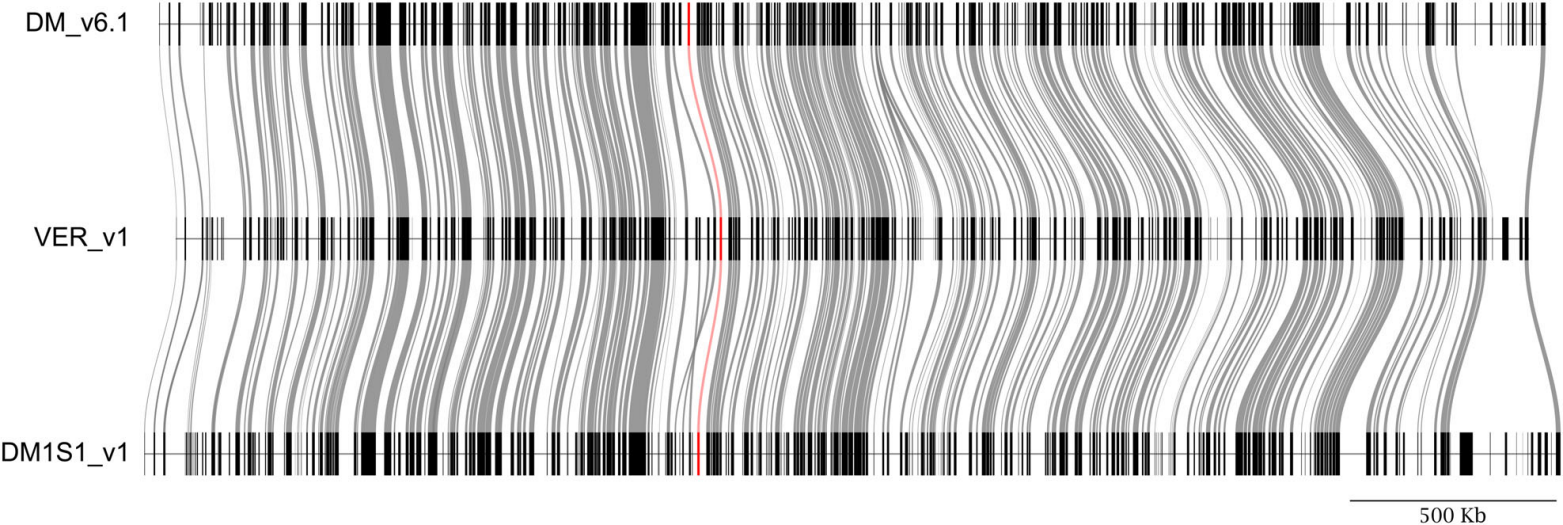
Synteny plot for the 1.5‐LOD supported interval for *ui11.1* on chromosome 11 across the reference genome DM 1‐3 516 R44 (DM_v6.1) and the two parents of the mapping population MSII1813‐2 (VER_v1) and DM1S1. The position of Soltu.DM.11G021610.1 across the three genomes is highlighted in red.

Within the 1.5‐LOD supported interval in the chromosome 11 *ui11.1* QTL, there are 254 genes in DMv6.1, 239 genes in *S. verrucosum*, and 240 in DM1S1; of these, 235 are present in a 1:1:1 syntenic relationship. Between the two parents of the F2 population, 223 genes are present in a 1:1 relationship between the two genomes, while 15 genes are novel to DM1S1 and 14 genes novel to *S. verrucosum* (Figure 7, Table S10). Within the peak QTL region, which spans approximately 464 026 bp, a search for DE genes revealed the candidate gene (Soltu.DM.11G021610.1) with an expression pattern correlating with the ability to accept interspecific pollen (Figure S8). Soltu.DM.11G021610.1 is a Fatty acyl‐CoA reductase (FAR) annotated as a Jojoba acyl CoA reductase‐related male sterility protein. FARs are enzymes that catalyze the reduction of fatty‐acyl‐CoA substrates into primary fatty alcohols and serve conserved roles in lipid synthesis in the formation of extracellular lipid barriers (Zhang et al., 2022). The MSII1813‐2 and DM1S1 orthologs of Soltu.DM.11G021610.1 are 99% identical, but the expression is significantly higher in MSII1813‐2 and MSJJ1821F2‐049 that lack prezygotic IRBs in the style, compared to DM1S1 and the other F2 progeny that have functional IRBs (Figures S8 and S9). While it is unclear how FARs would affect the ability to accept interspecific pollen, they have a well‐established role in male fertility in other plants, where lack of function leads to male sterility (Liu et al., 2019; Zhang et al., 2021). In *A. thaliana*, Soltu.DM.11G021610.1 is most similar to AT4G33790.1 or FAR3, which is highly expressed in the carpel of mature flowers (Klepikova et al., 2016).

### Replicated pollination trial—Variation in postzygotic reproductive barriers

**Figure 8:**
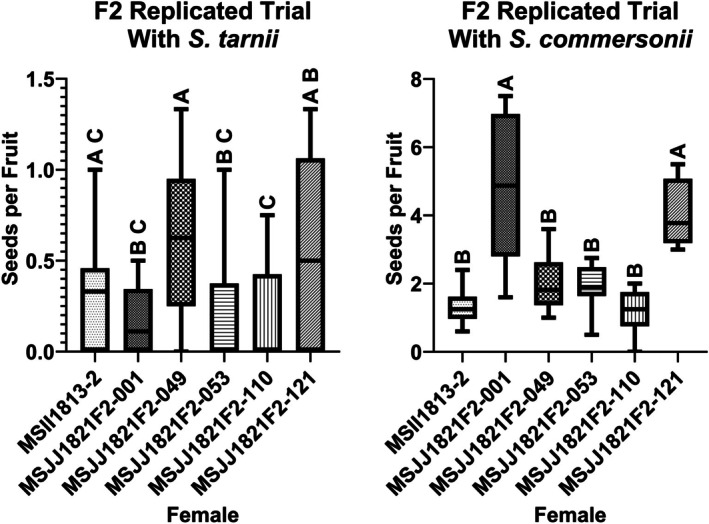
Results from a replicated pollination trial for variation in postzygotic interspecific reproductive barriers. Male parents (*S. tarnii* and *S. commersonii*) are listed at the top of each graph; seeds per fruit is depicted on the y‐axis, and the F2s tested along with the *S. verrucosum* parent MSII1813‐2 are represented on the x‐axis. Means comparisons with the same letter designation are not significantly different, as determined by Tukey's HSD (*α* = 0.05).

The replicated pollination trial sought to explore any variation in postzygotic IRBs present in the F2 progeny. All five F2 progeny examined lacked prezygotic IRBs, sharing the SvSC phenotype of the *S. verrucosum* parent. We observed significant variation between F2 progeny for postzygotic IRBs in terms of seeds per fruit (Figure 8). For example, in pollinations with *S. tarnii*, all F2 progeny in the replicated pollination trial (MSJJ1821F2‐001, MSJJ1821F2‐049, MSJJ1821F2‐053, MSJJ1821F2‐110, and MSJJ1821F2‐121) were not significantly different from the *S. verrucosum* parent MSII1813‐2 (Figure 8). However, significant differences in seed set were observed between the five F2 progeny; MSJJ1821F2‐049 produced significantly more hybrid seed per fruit compared to MSJJ1821F2‐001, MSJJ1821F2‐053, and MSJJ1821F2‐110, while producing a comparable number of seeds per fruit to MSJJ1821F2‐121.

Different patterns were also observed in interspecific reproductive success between the two male parents used in the experiment (*S. commersonii* and *S. tarnii*). This supports previous research that found *S. tarnii* (clade 1) and *S. commersonii* (clade 4) have significantly different responses to the same postzygotic IRBs in *S. verrucosum* (Behling et al., 2024; Spooner et al., 2004). Overall, pollinations between F2 progeny and *S. commersonii* were more likely to produce seed. Furthermore, the differences between F2 progeny, in terms of seed production, differed from pollinations with *S. tarnii*. In pollinations with *S. commersonii*, MSJJ1821F2‐001 and MSJJ1821F2‐121, produced significantly more seeds per fruit compared with the remaining F2 lines tested and the *S. verrucosum* parent, which produced a similar number of seeds per fruit (Figure 8). Segregation of alleles underpinning minor differences in effective ploidy between parents could explain the variation in seeds per fruit observed in the F2 (Behling et al., 2024). Fine mapping with larger mapping populations and multiple replications would be required to tease out genetic differences.

### General conclusions

Interspecific compatibility in *Solanum* is a complex trait with considerable overlap with self‐compatibility (Baek et al., 2015; Tovar‐Méndez et al., 2014). However, self‐compatibility alone cannot guarantee a loss of female IRBs, as the mechanisms that govern IRBs are varied and often specific to the parental species pairing (Behling, 2022; Behling & Douches, 2023; Tovar‐Méndez et al., 2014). The broad interspecific compatibility phenotype observed in *S. verrucosum* is unique and striking as it does not seem to vary according to the pollen parent used. In this population, the genetic basis for this broad interspecific compatibility was associated with two QTLs which together explain 56.6% of the variance observed; together they provide self‐compatibility via the loss of *S‐RNase* expression and a loss of IRBs comparable to the *S. verrucosum* parent. This is evidenced in the replicated pollination trial where all individuals were homozygous at the QTL locus on chromosome 1 for the *S. verrucosum S‐RNase* allele, and all but MSJJ1821F2‐110 were homozygous at the QTL locus on chromosome 11 for the *S. verrucosum ui11.1* allele.

We had expected to find a QTL on chromosome 12 corresponding to *HT* as previous research findings on IRBs in the tomato clade, which found *S‐RNase* and *HT* to be the primary determinants for IRBs (Tovar‐Méndez et al., 2014). Instead, we were surprised to find a significant QTL on chromosome 11, and no QTL on chromosome 12. No genes known to be associated with IRBs have been previously identified on chromosome 11 in *Solanum*. It is also unclear why *HT* was not a significant factor in this population but *ui11.1* was. We also identified several candidate genes associated with the QTLs for male fertility on chromosomes 3, 8, and 9. Candidate genes for these QTL are associated with pollen development and function and are ideal targets for further investigation into male fertility in potato.

Future research efforts should focus on determining the identity of *ui11.1*. The only significantly DE gene in the QTL region has no known association with GSI or IRBs (Table S11). The development of an RIL population from this F2 would provide a higher mapping resolution for *ui11.1* and be a useful tool for understanding post‐zygotic IRBs (Jansky et al., 2024; Kaiser et al., 2021). This would facilitate marker‐assisted selection for this trait, allowing for efficient integration into cultivated breeding populations. The full incorporation of SvSC into cultivated germplasm will allow breeders to cross species in the tertiary gene pool directly to cultivated breeding lines, eliminating the need for bridge crosses with *S. verrucosum* and accelerating the incorporation of favorable alleles.

## MATERIALS AND METHODS

### Plant materials

The MSJJ1821 mapping population was generated from a cross between the doubled monoploid *S. tuberosum* Group Phureja clone DM1S1 (female) and the *S. verrucosum* clone MSII1813‐2 (male) (Figure 1). The clone DM1S1 was generated at and provided by Virginia Tech University (Dr. Richard Veilleux) and is a doubled monoploid derived from the heterozygous clone 1S1 via anther culture and subsequent chromosome doubling. 1S1 and DM1S1 have been sequenced, and the genome assembly, annotation, and genomic data are publicly available (Jayakody et al., 2023). MSII1813‐2 is a clonal selection from the *S. verrucosum* PI 161173 and was selected for its ability to reliably produce interspecific hybrids with *S. pinnatisectum*. During the course of these experiments, MSII1813‐2 was sequenced, and a high‐quality chromosome scale assembly was created. Previous experiments demonstrated that 1S1 and its derived doubled monoploid DM1S1 have functioning prezygotic IRBs, while the *S. verrucosum* parent MSII1813‐2 does not (Figure S1). Three pollen donors were used to evaluate IRBs in F2 progeny: CMM2‐3, a *S. commersonii* selection made at Michigan State University from PI 473411; 347 766‐1, a *S. pinnatisectum* clone selected from PI 347766 at the USDA‐ARS Vegetable Crops Research Unit (Madison, WI) for its late blight (*Phytophthora infestans*) resistance; and for *S. tarnii*, a bulk population of five individuals from PI 570642. The clone 347766‐1 and the bulk population from PI 570642 were used to evaluate prezygotic IRBs for the F2 mapping population, and CMM2‐3 and the bulk population from PI 570642 were used as pollen donors for the replicated pollination experiment evaluating post‐zygotic IRBs.

### Greenhouse and tissue culture conditions

Clones used in these experiments were maintained in tissue culture utilizing Murashige and Skoog Basal Medium with vitamins and sucrose (PhytoTech Labs, Lexana, KS, USA) and Phyto Agar (Research Products International, Mount Prospect, IL, USA) prepared with DI water and balanced with 1 M HCl and 1 N NaOH to a pH of 5.8 and cultured in growth chambers with a 16‐hr light/8‐hr dark photoperiod at 22°C and an average light intensity of 200 μmoles m^−2^ s^−1^. For the duration of the experiments, greenhouse conditions were set at 20°C with a 16‐hr photoperiod under Philips GreenPower light‐emitting diode (LED) DR/W‐MB lights (Philips Lighting Holding B.V., Eindhoven, Netherlands).

### Generation of mapping population

The initial pollination between DM1S1 and MSII1813‐2 was made in December of 2021 (Figure 1). Approximately 8 weeks after pollination, mature fruit was harvested, and the F1 seed was extracted and allowed to dry thoroughly. F1 seed was treated with 1500 ppm GA3 to break seed dormancy and sown in 10.2 cm (4 inch) pots in March of 2022. A single F1 individual (MSJJ1821‐01) was selected as a parent for the F2 mapping population and was self‐pollinated in April 2022 to produce the F2 seed generation. In September of 2022, the F2 seed was treated with 1500 ppm GA3 and sown in 10.2 cm (4 inch) pots. Approximately 3 weeks after germination, 150 seedlings were transferred to 50‐cell trays. Seedlings were allowed to grow in the 50‐cell flats for 3 weeks before transferring them to 14 L (3.8 gallon) plastic pots filled with Suremix Perlite peat and perlite soilless medium (Michigan Grower Products INC., Galesburd, MI, USA) where the plants remained for the duration of the experiment.

The direction of the initial cross between DM1S1 and MSII1813‐2 is crucial to the utility of this population. When *S. verrucosum* is crossed to cultivated germplasm as the female, the progeny typically exhibit CMS (Abdalla & Hermsen, 1972). By using DM1S1 as the female, this ensures progeny will have cytoplasm compatible with a cultivated nuclear genome. DM1S1 is derived from *S. tuberosum* Group Phureja germplasm originally sourced from Columbia, likely containing P cytoplasm (Hosaka & Hanneman, 1988; Hosaka & Sanetomo, 2012; Paz & Veilleux, 1999).

### Interspecific compatibility phenotyping

The F2 generation was phenotyped in a stepwise process using self‐pollinations, pollen viability assays, and finally interspecific pollinations with *S. pinnatisectum* and *S. tarnii*. Self‐ and interspecific compatibility was phenotyped by pollinating at least two inflorescences of four or more flowers per individual (total *n* ≥ 9) for self‐pollinations, and at least six or more flowers on interspecific pollinations (total *n* ≥ 6), over a period of 48 days. Data on fruit per pollination, seeds per fruit, and seeds per pollination were collected for every pollination and used to calculate phenotypic classes. Individuals that produced greater than 0.2 fruit per selfed flower were classified as male fertile while the remaining were classified as male sterile. In interspecific pollinations, individuals that produced more than 0.2 fruit per pollinated flower were classified as having the *S. verrucosum* parental phenotype (SvSC). Pollen viability was assessed by collecting pollen from five to six mature anthers directly onto a glass slide, then immediately staining with acetocarmine‐glycerol as described by Ordonez (2014), covered with a cover slip, and sealed using clear nail polish. These slides were then stored at room temperature in the dark and visualized the same day under 10× and 40× magnification. A minimum of 100 pollen grains was used to calculate the percentage of viable pollen. Stained, turgid pollen is classified as viable while any grains that are shriveled, unstained, or unusually large or small are classified as unviable.

### Tissue collection, DNA extraction, and SNP genotyping

Total genomic DNA was isolated from freeze‐dried leaf samples of the parents and F2 progeny of the mapping population following the Mag‐Bind Plant DNA Plus 96 Kit protocol (Omega Bio‐tek, Norcross, GA, USA). Single nucleotide polymorphism (SNP) genotyping was performed using the Illumina Infinium SolCAP V4 30K Potato SNP Array using the Illumina iScan Reader (Illumina, San Diego, CA) following the manufacturer's protocol. The Illumina GenomeStudio v2.0.5 software was used for calling three cluster SNP genotypes (Illumina) for a diploid model. The genotype calls were filtered to exclude SNPs categorized as poor‐quality markers and those with >10% missing data. A total of 7322 quality‐filtered, segregating SNPs were used for analysis (Hirsch et al., 2013).

### Linkage map and QTL analysis

Because both parents of this population are homozygous, this is a true F2 population which is unusual in potato. The 7322 quality‐filtered SNPs were recoded as (a, h, b) for mapping as an F2 population using JoinMap 5 software (Kyazma B.V., Wageningen, Netherlands). Non‐segregating and co‐segregating SNPs as well as SNPs that mapped to chr00 on the DM v4.03 pseudomolecule were excluded from analysis. Twelve linkage groups were generated using the final filtered, segregating 1937 SNPs using the maximum likelihood mapping feature with default parameters. The physical position of mapped SNPs for DM1‐3516 v4.03 pseudomolecules was used to compare genetic and physical maps. MapQTL 6 (Kyazma B.V.) was used for QTL analysis using map files created in JoinMap. The interspecific compatibility and self‐fertility phenotype data were used for QTL interval mapping. The genome‐wide permutation test interval values were used to determine the LOD significance threshold at the 0.05 level. The QTL figures with LOD scores plotted across the genetic map position of the 12 chromosomes were generated using JMP Pro 17 (SAS Institute Inc, Cary, NC, USA).

### Transmission ration distortion (TRD)

The 7322 quality‐filtered SNPs were further filtered to remove outliers leaving a total of 6983 SNPs to evaluate TRD. Deviation from the expected 1:2:1 Mendelian genotypic class frequencies was determined using *χ* ^2^ tests for each SNP marker with 2 degrees of freedom and a *P*‐value of *α* = 0.05. Significance thresholds were calculated using Bonferroni multiple test correction utilizing an *α* = 0.05/6983 SNPs = 7.16 × 10^−6^ (*χ* ^2^ = 23.69, df = 2). Haplotypes were plotted over physical distance (Mb) for each chromosome according to DM v4.03 assembly using JMP Pro 17 (SAS Institute Inc).

### Tissue collection, RNA extraction, and sequencing

Total RNA was isolated from styles and pollen of the parents of the F2 population and three F2 individuals representing the range of phenotypic variation within the F2 population: MSJJ1821F2‐041 = male sterile with functional IRBs, MSJJ1821F2‐049 = male fertile and absence of IRBs (SvSC), and MSJJ1821F2‐091 = male fertile with functional IRBs. For each plant, at least 30 styles and 100 μl were collected from newly opened flowers to remain consistent with phenotyping conditions. Tissue samples were harvested directly into liquid nitrogen and stored at −80°C until processing. Total RNA was isolated from collected tissue using the Qiagen RNeasy Plant Mini Kit (Qiagen, Hilden, Germany) following the recommended protocol provided by the manufacturer. RNA was quantified using the Qubit RNA Broad Range Assay Kit (Thermo Fisher Scientific, Waltham, MA, USA). RNA quality was evaluated on the Agilent 4200 TapeStation using the high sensitivity RNA assay (Agilent Technologies, Santa Clara, CA, USA). Library preparation and sequencing were performed by Azenta (Genewiz, South Plainfield, New Jersey, USA) on the Illumina NovaSeq platform (150 bp paired‐end sequencing) to obtain 20–30 million reads per sample.

### *S. verrucosum* MSII1813‐2 genome sequencing and assembly

High molecular weight genomic DNA was isolated from *S. verrucosum* MSII1813‐2 (PI 161173) immature leaves and shoot tips. Plants were grown in a greenhouse at 20°C, 16‐h light, 8‐h dark followed by a 24‐h dark treatment immediately before harvest. DNA was isolated via a crude CTAB extraction applied to a Qiagen 500/g Genomic‐tip (Qiagen, Germantown, MD, USA) followed by an Amicon buffer exchange (Millipore, Burlington, MA, USA) (Vaillancourt & Buell, 2019). PacBio HiFi libraries were prepared using the PacBio HiFi Express Kit 2.0 and sequenced on two SMRT cells on the PacBio Sequel II (Menlo Park, CA, USA) by the University of Minnesota Genomics Center (Minneapolis, MN, USA), yielding a total of 48.62 Gb with a N50 of 26 kb. PacBio HiFi reads were assembled using hifiasm v0.19.7 (Cheng et al., 2021, 2022) with a similarity threshold (−s) set to 0.2. Assembly contigs less than 50 kb in size were discarded using SeqKit v2.3.0 (Shen et al., 2016, 2024). Ragtag v2.1.0 (Alonge et al., 2022) was run twice to scaffold the assembly using the scaffold function with the DM 1‐3 516 R44 v6.1 genome assembly (Pham et al., 2020). Assembly statistics were calculated using assembly‐stats (https://github.com/sanger‐pathogens/assembly‐stats). BUSCO v5.4.0 (Manni, Berkeley, Seppey, Simão, & Zdobnov, 2021; Manni, Berkeley, Seppey, & Zdobnov, 2021) along with the embryophyta_odb10 database was used to evaluate the completeness of the genome assembly. Both PacBio HiFi reads and the genome assembly were split into 150 bp fragments using SeqKit v2.3.0 (Shen et al., 2016) and input into Kraken v2.1.2 (Wood et al., 2019) with the k2_pluspfp_20220908 database (https://benlangmead.github.io/aws‐indexes/k2) to check for contamination. D‐Genies (Cabanettes & Klopp, 2018) was used to visualize the successful scaffolding of the genome assembly against the DM genome using the output from minimap2‐x asm10‐secondary = no v2.23‐r1111 (Li, 2021) and samtools v1.12 (Danecek et al., 2021).

### *S. verrucosum* MSII1813‐2 genome annotation

The *S. verrucosum* MSII1813‐2 genome assembly was repeat masked by first creating a custom repeat library (CRL) for the genome. Repeats were first identified with RepeatModeler (v2.03; https://github.com/Dfam‐consortium/RepeatModeler) (Flynn et al., 2020) and protein‐coding genes filtered out from the repeat database using ProtExcluder v1.2 (Campbell et al., 2014) to create a CRL. The CRL was then combined with Viridiplantae repeats from RepBase v20150807 (Bao et al., 2015) to generate the final CRL for the genome. The genome assembly was repeat‐masked using the respective final CRL and RepeatMasker (v4.1.2‐p1, https://www.repeatmasker.org/RepeatMasker) (Tarailo‐Graovac & Chen, 2009) using the parameters ‐e ncbi ‐s ‐nolow ‐no_is ‐gff.

RNA‐seq libraries were processed for genome annotation by first cleaning with Cutadapt v4.6 (Martin, 2011) using a minimum length of 100 nt and quality cutoff of 10, then aligning the cleaned reads to the genome assembly using HISAT2 v2.2.1 (Kim et al., 2019). The aligned RNA‐seq reads were assembled using Stringtie v2.2.1 (Kovaka et al., 2019) and transcripts less than 500 nt were removed.

The gene models were created using BRAKER2 v2.1.6 (Brůna et al., 2021) using the soft‐masked genome assembly and the aligned RNA‐seq libraries as hints. Functional annotation was assigned by searching the working gene model proteins against the TAIR v10 (Lamesch et al., 2011) database and the Swiss‐Prot plant proteins (release 2015_08) database using BLASTP v2.12.0 (Altschul et al., 1990) and the PFAM v35.0 (EL‐Gebali et al., 2018) database using PfamScan v1.6 (Li et al., 2015) and assigning the annotation based on the first significant hit. BUSCO v5.4.0 (Manni, Berkeley, Seppey, Simão, & Zdobnov, 2021; Manni, Berkeley, Seppey, & Zdobnov, 2021) along with the embryophyta_odb10 database was used to evaluate the completeness of the genome annotation.

### RNA‐seq data processing and differential gene expression analysis

The quality of the raw RNA‐seq data was inspected initially using FastQC (Andrews, 2010) Reads were then trimmed using Trimmomatic v3.39 (Bolger et al., 2014) using default settings except for LEADING:3, TRAILING:3, SLIDINGWINDOW:4:20, and MINLEN:36 to remove low‐quality bases and Illumina universal paired‐end adapters. Trimmed sequences were then quality checked using FastQC and mapped to their respective parental genomes. The DM1S1 v1 genome and annotation were retrieved from Spud DB (http://spuddb.uga.edu/). Trimmed RNA‐seq reads from MSII1813‐2 and MSJJ1821F2‐049 were mapped to the *S. verrucosum* genome assembly using TopHat2 (Kim et al., 2013), and those from DM1S1, MSJJ1821F2‐041, and MSJJ1821F2‐091 to the DM1S1 v1 genome assembly. After mapping, custom scripts were used to remove reads that mapped to multiple genes as well as any reads with lower mapping quality (>60). Mapped reads were then processed using HTseq (Anders et al., 2014) to obtain the read counts per gene.

To call differential expression between DM1S1 and *S. verrucosum* genomes, we first did a BLAST search (Altschul et al., 1990) between coding DNA (cDNA) sequences of all the genes of DM1S1 and *S. verrucosum* to identify the orthologous genes between the two parents. These orthologous genes were identified by making a BLAST database using the cDNA sequences for all of the genes in the DM1S1 genome. We then used this DM1S1 cDNA BLAST database to search for the best‐matching sequences (*E*‐value >1e^−3^) in the *S. verrucosum* genome assembly. DM1S1 sequences with the highest sequence similarity to *S. verrucosum* cDNA sequences were classified as orthologous genes. Finally, differential gene expression was calculated using the DESeq2 package (Love et al., 2014) and custom scripts in R. The differential expression comparisons between *S. verrucosum* and DM1S1 samples were carried out by measuring differential gene expression between respective orthologous genes.

### Identifying gene candidates for Chr 1, 8, and 11

The differential expression data were used in conjunction with the QTL position and SNP genotype data to identify candidate genes associated with the loss of IRBs on Chr 1 and 11 using an R script. For chromosome 1, DE genes were first filtered to exclude genes outside of the QTL region based on their position in the parental MSII1813‐2 genome. Then, using a significance threshold of *α* = 0.01, we selected genes that were significantly DE between DM1S1 and the F2 progeny MSJJ1821F2‐041 and MSJJ1821F2‐049, but were not significantly DE between DM1S1 and MSJJ1821F2‐091. The resulting list of genes was then filtered to select genes that were not significantly DE between MSII1813‐2 and the F2 progeny MSJJ1821F2‐041 and MSJJ1821F2‐049 and not significantly DE between MSII1813‐2 and MSJJ1821F2‐091. A similar analysis was used to identify candidate genes on chromosome 11. DE genes were first filtered to exclude those outside of the chromosome 11 QTL region, and using a criterion based on the SNP genotypes and phenotypes of the F2s, significantly DE genes were selected. For MSJJ1821‐041, we selected genes that were not significantly DE from DM1S1 and significantly DE from MSII1813‐2. We then added DE comparisons from MSJJ1821‐049 that were significantly DE from DM1S1 and not significantly DE from MSII1813‐2.

On Chr 8, representative protein sequencing from working gene models was obtained from *S. tuberosum* DM 1‐3 516 R44 (Pham et al., 2020), *S. tuberosum* RH89‐039‐16 (Zhou et al., 2020), *S. tuberosum* DM1S1 (Jayakody et al., 2023), *Solanum chacoense* M6 (https://spuddb.uga.edu/), *S. verrucosum*, and the outgroup *S. lycopersicum* M82 (https://spuddb.uga.edu/). A gene tree of pectinactylesterases was generated using Orthofinder v.2.5.5 (Emms & Kelly, 2019) with the options ‐M msa and ‐T raxml. Multiple sequence alignments were performed using MAFFT v7.520 (Katoh & Standley, 2013) and phylogeny using RAxML v8.2.12 (Stamatakis, 2014).

Synteny between the *S. verrucosum*, DM1S1 (Jayakody et al., 2023), and DMv6.1 (Pham et al., 2020) reference genomes was determined using GENESPACE (v1.3.1) (Lovell et al., 2022).

### Statistical analysis of genotypic and phenotypic correlations

Where appropriate, statistical analyses were made between phenotypic classes and genotypic classes using Fisher's exact test. SNP genotype and phenotypic class data were used to generate contingency tables for these analyses using GraphPad Prism v10.2.3 software (GraphPad Software, Boston, MA, USA).

### Replicated interspecific compatibility pollination experiment

Five F2 progeny with the *S. verrucosum* interspecific compatibility phenotype (SvSC) were selected to evaluate postzygotic barriers (MSJJ1821F2‐001, MSJJ1821F2‐049, MSJJ1821F2‐053, MSJJ1821F2‐110, and MSJJ1821F2‐121). F2 progeny were selected based on vigor, flower production, and susceptibility to root pathogens. In December 2022, stolons were used to propagate five individuals for each F2 genotype (total *n* = 25). Pollen donors were transplanted to the greenhouse from tissue culture at the same time. During the month of February 2023, the five F2 genotypes were pollinated using pollen from the *S. commersonii* clone CMM2‐3 (total *n* = 226) and the bulk *S. tarnii* population derived from PI 570642 (total *n* = 495). Resultant fruit were allowed to ripen for 8 weeks before harvesting, after which any resulting hybrid seed was extracted.

## CONFLICT OF INTEREST

The authors declare no conflict of interest.

## Supporting information


**Data S1.** Synteny results from GENESPACE.

**Figure S1.** Behavior of *S. pinnatisectum* pollen tubes in the styles of the parents, F1, and F2 progeny.
**Figure S2.** Phylogeny of *S‐RNase* CDS sequences from parents of the mapping population.
**Figure S3.** Phylogeny of *SLF* sequences, showing the relationship of parental *SLF* sequences used in the mapping population.
**Figure S4.** Mosaic plots showing the distribution of QTL haplotypes across phenotypic classes.
**Figure S5.** Multiple sequence alignment of Soltu.DM.03G036770.1 and the DM1S1 and MSII1813‐2 orthologs.
**Figure S6.** Gene tree of pectinacetylesterases from *S. verrucosum* MSII1813‐2, *S. chacoense* M6, *S. tuberosum* DM, *S. tuberosum* DM1S1, *S. tuberosum* RH, and *Solanum lycopersicum* M82 that was used as an outgroup.
**Figure S7.** Multiple sequence alignment of Soltu.DM.09G000840.1 and the DM1S1 and MSII1813‐2 orthologs.
**Figure S8.** Heat map of Log_2_ fold change of *ui11.1* candidate gene Soltu.DM.11G021610.1 in the chromosome 11 QTL.
**Figure S9.** Multiple sequence alignment of Soltu.DM.11G021610 and the DM1S1 and MSII1813‐2 orthologs.
**Table S1.** Pollination phenotyping data for the F2 mapping population.
**Table S2.** List of significant SNPs, their physical and map positions, LOD values, and nearest annotation on DM v6.1 assembly.
**Table S3.** *S‐RNase* and *SLF* sequences from parents used in the mapping population used in the construction of phylogenies.
**Table S4.** *S‐RNase* and *SLF* sequences used as comparison for the construction of phylogenies.
**Table S5.** Transcripts per million (TPM) values for pollen expressed Solver.v1.03_VERG035240.1.
**Table S6.** Genome assembly metrics for *S. verrucosum* MSII1813‐2.
**Table S7.** Benchmarking universal single copy orthologs in the *S. verrucosum* MSII1813‐2 genome sequence and annotation.
**Table S8.** Repetitive sequences identified in *S. verrucosum* MSII1813‐2.
**Table S9.** Protein coding genes annotated in *S. verrucosum* MSII1813‐2.
**Table S10.** Syntenic genes within the chromosome 11 QTL for ui11.1.
**Table S11.** List of significantly differentially expressed genes (*α* = 0.01) in the styles of F2 progeny and parents used in the RNA‐seq and differential expression analysis.

## Data Availability

The clone DM1S1 and the PI for MSII1813‐2 are available through the United States Department of Agriculture Potato Genebank. PacBio HiFi sequencing data for MSII1813‐2 and RNA sequencing data are available via the NCBI Sequence Read Archive under BioProject PRJNA1153436 and PRJNA1234657, respectively. Genome sequence and annotation are available on Figshare under https://doi.org/10.6084/m9.figshare.29815418.v1. All code used for RNA‐seq mapping and data processing can be found in GitHub repositories: https://github.com/ShiuLab/RNA‐seq_data_processing.git and https://github.com/Thilanka‐lt/William_et_al_2025.git, respectively.

## References

[tpj70426-bib-0001] Abdalla, M.M.F. & Hermsen, J.G.T. (1972) Plasmons and male sterility types in *Solanum verrucosum* and its interspecific hybrid derivatives. Euphytica, 21, 209–220.

[tpj70426-bib-0002] Abdalla, M.M.F. & Hermsen, J.G.T. (1973) An evaluation of *Solanum verrucosum* Schlechtd. For its possible use in potato breeding. Euphytica, 22, 19–27.

[tpj70426-bib-0003] Alonge, M. , Lebeigle, L. , Kirsche, M. , Jenike, K. , Ou, S. , Aganezov, S. et al. (2022) Automated assembly scaffolding using RagTag elevates a new tomato system for high‐throughput genome editing. Genome Biology, 23, 258. 36522651 10.1186/s13059-022-02823-7 PMC9753292

[tpj70426-bib-0004] Altschul, S.F. , Gish, W. , Miller, W. , Myers, E.W. & Lipman, D.J. (1990) Basic local alignment search tool. Journal of Molecular Biology, 215, 403–410. 2231712 10.1016/S0022-2836(05)80360-2

[tpj70426-bib-0005] Anders, S. , Pyl, P.T. & Huber, W. (2014) HTSeq—a python framework to work with high‐throughput sequencing data. Bioinformatics, 31, 166–169. 25260700 10.1093/bioinformatics/btu638 PMC4287950

[tpj70426-bib-0090] Andrews, S. , Krueger, F. , Segonds‐Pichon, A. , Biggins, L. , Krueger, C. & Wingett, S. (2010) FastQC. A quality control tool for high throughput sequence data, 370.

[tpj70426-bib-0007] Baek, Y.S. , Covey, P.A. , Petersen, J.J. , Chetelat, R.T. , Mcclure, B. & Bedinger, P.A. (2015) Testing the SI × SC rule: pollen–pistil interactions in interspecific crosses between members of the tomato clade (Solanum section Lycopersicon, Solanaceae). American Journal of Botany, 102, 302–311. 25667082 10.3732/ajb.1400484

[tpj70426-bib-0008] Bai, M. , Gao, H. , Yang, Y. & Wu, H. (2023) Changes in the content of pollen total lipid and TAG in Arabidopsis thaliana DGAT1 mutant as11. AoB Plants, 15(2), plad012. 37064996 10.1093/aobpla/plad012 PMC10100649

[tpj70426-bib-0009] Bamberg, J. , Kielar, A. , Del Rio, A. & Douches, D. (2021) Making hybrids with the wild potato *Solanum jamesii* . American Journal of Potato Research, 98, 187–193.

[tpj70426-bib-0010] Bao, W. , Kojima, K.K. & Kohany, O. (2015) Repbase update, a database of repetitive elements in eukaryotic genomes. Mobile DNA, 6, 11. 26045719 10.1186/s13100-015-0041-9 PMC4455052

[tpj70426-bib-0011] Behling, W. (2022) The Role of Prezygotic Self‐Compatibility in Facilitating Interspecific Compatibility in Solanum Section Petota. M.S., Michigan State University. Available from: https://www.proquest.com/openview/aa5d1fec843794bdcf377c04189569f9/1?pq-origsite=gscholar&cbl=18750&diss=y [Accessed 15th August 2025].

[tpj70426-bib-0012] Behling, W. , Coombs, J. , Collins, P. & Douches, D. (2024) An analysis of inter‐endosperm balance number crosses with the wild potato *Solanum verrucosum* . American Journal of Potato Research, 101, 34–44.

[tpj70426-bib-0013] Behling, W.L. & Douches, D.S. (2023) The effect of self‐compatibility factors on interspecific compatibility in solanum section Petota. Plants, 12, 1709. 37111931 10.3390/plants12081709 PMC10144722

[tpj70426-bib-0014] Bethke, P.C. , Halterman, D.A. & Jansky, S. (2017) Are we getting better at using wild potato species in light of new tools? Crop Science, 57, 1241–1258.

[tpj70426-bib-0015] Bolger, A.M. , Lohse, M. & Usadel, B. (2014) Trimmomatic: a flexible trimmer for Illumina sequence data. Bioinformatics, 30, 2114–2120. 24695404 10.1093/bioinformatics/btu170 PMC4103590

[tpj70426-bib-0016] Brůna, T. , Hoff, K.J. , Lomsadze, A. , Stanke, M. & Borodovsky, M. (2021) BRAKER2: automatic eukaryotic genome annotation with GeneMark‐EP+ and AUGUSTUS supported by a protein database. NAR Genomics and Bioinformatics, 3, lqaa108. 33575650 10.1093/nargab/lqaa108 PMC7787252

[tpj70426-bib-0018] Cabanettes, F. & Klopp, C. (2018) D‐GENIES: dot plot large genomes in an interactive, efficient and simple way. PeerJ, 6, e4958. 29888139 10.7717/peerj.4958 PMC5991294

[tpj70426-bib-0019] Campbell, M.S. , Holt, C. , Moore, B. & Yandell, M. (2014) Genome annotation and curation using MAKER and MAKER‐P. Current Protocols in Bioinformatics, 48, 4.11.1–4.11.39. 10.1002/0471250953.bi0411s48 PMC4286374 25501943

[tpj70426-bib-0020] Cankar, K. , Kortstee, A. , Toonen, M.A.J. , Wolters‐Arts, M. , Houbein, R. , Mariani, C. et al. (2014) Pectic arabinan side chains are essential for pollen cell wall integrity during pollen development. Plant Biotechnology Journal, 12, 492–502. 24428422 10.1111/pbi.12156

[tpj70426-bib-0021] Cheng, H. , Concepcion, G.T. , Feng, X. , Zhang, H. & Li, H. (2021) Haplotype‐resolved de novo assembly using phased assembly graphs with hifiasm. Nature Methods, 18, 170–175. 33526886 10.1038/s41592-020-01056-5 PMC7961889

[tpj70426-bib-0022] Cheng, H. , Jarvis, E.D. , Fedrigo, O. , Koepfli, K.‐P. , Urban, L. , Gemmell, N.J. et al. (2022) Haplotype‐resolved assembly of diploid genomes without parental data. Nature Biotechnology, 40, 1332–1335. 10.1038/s41587-022-01261-x PMC9464699 35332338

[tpj70426-bib-0023] Cipar, M.S. , Peloquin, S.J. & Hougas, R.W. (1964) Variability in the expression of self‐incompatibility in tuber‐bearing diploid Solanum species. American Potato Journal, 41, 155–162.

[tpj70426-bib-0024] Clot, C.R. , Wang, X. , Koopman, J. , Navarro, A.T. , Bucher, J. , Visser, R.G.F. et al. (2024) High‐density linkage map constructed from a skim sequenced diploid potato population reveals transmission distortion and QTLs for tuber yield and pollen shed. Potato Research, 67, 139–163.

[tpj70426-bib-0025] Danecek, P. , Bonfield, J.K. , Liddle, J. , Marshall, J. , Ohan, V. , Pollard, M.O. et al. (2021) Twelve years of SAMtools and BCFtools. GigaScience, 10(2), giab008. 33590861 10.1093/gigascience/giab008 PMC7931819

[tpj70426-bib-0026] Dinu, I.I. , Hayes, R.J. , Kynast, R.G. , Phillips, R.L. & Thill, C.A. (2005) Novel inter‐series hybrids in Solanum, section Petota. Theoretical and Applied Genetics, 110, 403–415. 15517147 10.1007/s00122-004-1782-x

[tpj70426-bib-0027] Eijlander, R. , Ter Laak, W. , Hermsen, J.G.T. , Ramanna, M.S. & Jacobsen, E. (2000) Occurrence of self‐compatibility, self‐incompatibility and unilateral incompatibility after crossing diploid *S. tuberosum* (SI) with *S. verrucosum* (SC): I. Expression and inheritance of self‐compatibility. Euphytica, 115, 127–139.

[tpj70426-bib-0028] EL‐Gebali, S. , Mistry, J. , Bateman, A. , Eddy, S.R. , Luciani, A. , Potter, S.C. et al. (2018) The Pfam protein families database in 2019. Nucleic Acids Research, 47(D1), D427–D432. 10.1093/nar/gky995 PMC6324024 30357350

[tpj70426-bib-0029] Emms, D.M. & Kelly, S. (2019) OrthoFinder: phylogenetic orthology inference for comparative genomics. Genome Biology, 20, 1–14. 31727128 10.1186/s13059-019-1832-y PMC6857279

[tpj70426-bib-0030] Endelman, J.B. , Kante, M. , Lindqvist‐Kreuze, H. , Kilian, A. , Shannon, L.M. , Caraza‐Harter, M.V. et al. (2024) Targeted genotyping‐by‐sequencing of potato and data analysis with R/polyBreedR. The Plant Genome, 17(3), e20484. 38887158 10.1002/tpg2.20484 PMC12807073

[tpj70426-bib-0031] Flynn, J.M. , Hubley, R. , Goubert, C. , Rosen, J. , Clark, A.G. , Feschotte, C. et al. (2020) RepeatModeler2 for automated genomic discovery of transposable element families. Proceedings of the National Academy of Sciences, 117, 9451–9457. 10.1073/pnas.1921046117 PMC7196820 32300014

[tpj70426-bib-0032] Goldraij, A. , Kondo, K. , Lee, C.B. , Hancock, C.N. , Sivaguru, M. , Vazquez‐Santana, S. et al. (2006) Compartmentalization of S‐RNase and HT‐B degradation in self‐incompatible nicotiana. Nature, 439, 805–810. 16482149 10.1038/nature04491

[tpj70426-bib-0033] Gou, J.‐Y. , Miller, L.M. , Hou, G. , Yu, X.‐H. , Chen, X.‐Y. & Liu, C.‐J. (2012) Acetylesterase‐mediated deacetylation of pectin impairs cell elongation, pollen germination, and plant reproduction. The Plant Cell, 24, 50–65. 22247250 10.1105/tpc.111.092411 PMC3289554

[tpj70426-bib-0034] Hermsen, J.G.T. & Ramanna, M.S. (1976) Barriers to hybridization of *Solanum bulbocastanum* dun. and *S. verrucosum* Schlechtd. and structural hybridity in their F1 plants. Euphytica, 25, 1–10.

[tpj70426-bib-0035] Hirsch, C.N. , Hirsch, C.D. , Felcher, K. , Coombs, J. , Zarka, D. , Van Deynze, A. et al. (2013) Retrospective view of north American potato (*Solanum tuberosum* L.) breeding in the 20th and 21st centuries. G3: Genes, Genomes, Genetics, 3, 1003–1013. 23589519 10.1534/g3.113.005595 PMC3689798

[tpj70426-bib-0036] Hosaka, A.J. , Sanetomo, R. & Hosaka, K. (2022) A de novo genome assembly of *Solanum verrucosum* Schlechtendal, a Mexican diploid species geographically isolated from other diploid A‐genome species of potato relatives. G3: Genes, Genomes, Genetics, 12, jkac166. 35775942 10.1093/g3journal/jkac166 PMC9339273

[tpj70426-bib-0037] Hosaka, K. & Hanneman, R.E. (1988) Origin of chloroplast DNA diversity in the Andean potatoes. Theoretical and Applied Genetics, 76, 333–340. 24232196 10.1007/BF00265332

[tpj70426-bib-0038] Hosaka, K. & Sanetomo, R. (2012) Development of a rapid identification method for potato cytoplasm and its use for evaluating Japanese collections. Theoretical and Applied Genetics, 125, 1237–1251. 22696007 10.1007/s00122-012-1909-4

[tpj70426-bib-0039] Huang, B. , Ruess, H. , Liang, Q. , Colleoni, C. & Spooner, D.M. (2019) Analyses of 202 plastid genomes elucidate the phylogeny of Solanum section Petota. Scientific Reports, 9, 4454. 30872631 10.1038/s41598-019-40790-5 PMC6418237

[tpj70426-bib-0040] Jansky, S. & Hamernik, A. (2009) The introgression of 2× 1EBN solanum species into the cultivated potato using *Solanum verrucosum* as a bridge. Genetic Resources and Crop Evolution, 56, 1107–1115.

[tpj70426-bib-0041] Jansky, S. , Hamernik, A. & Endelman, J.B. (2024) Diploid interspecific recombinant inbred lines for genetic mapping in potato. American Journal of Potato Research, 101, 153–161.

[tpj70426-bib-0042] Jayakody, T.B. , Hamilton, J.P. , Jensen, J. , Sikora, S. , Wood, J.C. , Douches, D.S. et al. (2023) Genome report: genome sequence of 1S1, a transformable and highly regenerable diploid potato for use as a model for gene editing and genetic engineering. G3: Genes, Genomes, Genetics, 13, jkad036. 36755392 10.1093/g3journal/jkad036 PMC10085803

[tpj70426-bib-0043] Johnston, S.A. , Den Nijs, T.P.M. , Peloquin, S.J. & Hanneman, R.E. (1980) The significance of genic balance to endosperm development in interspecific crosses. Theoretical and Applied Genetics, 57, 5–9. 24302359 10.1007/BF00276002

[tpj70426-bib-0044] Kaiser, N.R. , Billings, G. , Coombs, J. , Buell, C.R. , ENCISO‐Rodríguez, F. & Douches, D.S. (2021) Self‐fertility and resistance to the Colorado potato beetle (*Leptinotarsa decemlineata*) in a diploid *Solanum chacoense* recombinant inbred line population. Crop Science, 61, 3392–3414.

[tpj70426-bib-0045] Katoh, K. & Standley, D.M. (2013) MAFFT multiple sequence alignment software version 7: improvements in performance and usability. Molecular Biology and Evolution, 30(4), 772–780. 23329690 10.1093/molbev/mst010 PMC3603318

[tpj70426-bib-0046] Kim, D. , Paggi, J.M. , Park, C. , Bennett, C. & Salzberg, S.L. (2019) Graph‐based genome alignment and genotyping with HISAT2 and HISAT‐genotype. Nature Biotechnology, 37, 907–915. 10.1038/s41587-019-0201-4 PMC7605509 31375807

[tpj70426-bib-0047] Kim, D. , Pertea, G. , Trapnell, C. , Pimentel, H. , Kelley, R. & Salzberg, S.L. (2013) TopHat2: accurate alignment of transcriptomes in the presence of insertions, deletions and gene fusions. Genome Biology, 14, R36. 23618408 10.1186/gb-2013-14-4-r36 PMC4053844

[tpj70426-bib-0048] Klepikova, A.V. , Kasianov, A.S. , Gerasimov, E.S. , Logacheva, M.D. & Penin, A.A. (2016) A high resolution map of the *Arabidopsis thaliana* developmental transcriptome based on RNA‐seq profiling. The Plant Journal, 88(6), 1058–1070. 27549386 10.1111/tpj.13312

[tpj70426-bib-0049] Knoch, E. , Sugawara, S. , Mori, T. , Nakabayashi, R. , Saito, K. & YONEKURA‐Sakakibara, K. (2018) UGT79B31 is responsible for the final modification step of pollen‐specific flavonoid biosynthesis in *Petunia hybrida* . Planta, 247, 779–790. 29214446 10.1007/s00425-017-2822-5 PMC5856881

[tpj70426-bib-0050] Kovaka, S. , Zimin, A.V. , Pertea, G.M. , Razaghi, R. , Salzberg, S.L. & Pertea, M. (2019) Transcriptome assembly from long‐read RNA‐seq alignments with StringTie2. Genome Biology, 20, 278. 31842956 10.1186/s13059-019-1910-1 PMC6912988

[tpj70426-bib-0051] Kubo, K.‐I. , Entani, T. , Takara, A. , Wang, N. , Fields, A.M. , Hua, Z. et al. (2010) Collaborative non‐self recognition system in S‐RNase–based self‐incompatibility. Science, 330, 796–799. 21051632 10.1126/science.1195243

[tpj70426-bib-0052] Lamesch, P. , Berardini, T.Z. , Li, D. , Swarbreck, D. , Wilks, C. , Sasidharan, R. et al. (2011) The Arabidopsis information resource (TAIR): improved gene annotation and new tools. Nucleic Acids Research, 40, D1202–D1210. 22140109 10.1093/nar/gkr1090 PMC3245047

[tpj70426-bib-0053] Lee, S. (2021) Generation of HT‐B and HT‐B Plus S‐RNase knockout lines to understand self‐compatibility in diploid potato. M.S., Michigan State University.

[tpj70426-bib-0054] Lee, S. , ENCISO‐Rodriguez, F.E. , Behling, W. , Jayakody, T. , Panicucci, K. , Zarka, D. et al. (2023) HT‐B and S‐RNase CRISPR‐Cas9 double knockouts show enhanced self‐fertility in diploid *Solanum tuberosum* . Frontiers in Plant Science, 14, 1151347. 37324668 10.3389/fpls.2023.1151347 PMC10264808

[tpj70426-bib-0055] Li, H. (2021) New strategies to improve minimap2 alignment accuracy. Bioinformatics, 37, 4572–4574. 34623391 10.1093/bioinformatics/btab705 PMC8652018

[tpj70426-bib-0056] Li, W. , Cowley, A. , Uludag, M. , Gur, T. , Mcwilliam, H. , Squizzato, S. et al. (2015) The EMBL‐EBI bioinformatics web and programmatic tools framework. Nucleic Acids Research, 43, W580–W584. 25845596 10.1093/nar/gkv279 PMC4489272

[tpj70426-bib-0057] Liu, F. , Ma, L. , Wang, Y. , Li, Y. , Zhang, X. , Xue, F. et al. (2019) GhFAD2–3 is required for anther development in *Gossypium hirsutum* . BMC Plant Biology, 19, 1–17. 31500565 10.1186/s12870-019-2010-9 PMC6734329

[tpj70426-bib-0058] Love, M.I. , Huber, W. & Anders, S. (2014) Moderated estimation of fold change and dispersion for RNA‐seq data with DESeq2. Genome Biology, 15, 550. 25516281 10.1186/s13059-014-0550-8 PMC4302049

[tpj70426-bib-0059] Lovell, J.T. , Sreedasyam, A. , Schranz, M.E. , Wilson, M. , Carlson, J.W. , Harkess, A. et al. (2022) GENESPACE tracks regions of interest and gene copy number variation across multiple genomes. eLife, 11, e78526. 36083267 10.7554/eLife.78526 PMC9462846

[tpj70426-bib-0060] Manni, M. , Berkeley, M.R. , Seppey, M. , Simão, F.A. & Zdobnov, E.M. (2021) BUSCO update: novel and streamlined workflows along with broader and deeper phylogenetic coverage for scoring of eukaryotic, prokaryotic, and viral genomes. Molecular Biology and Evolution, 38, 4647–4654. 34320186 10.1093/molbev/msab199 PMC8476166

[tpj70426-bib-0061] Manni, M. , Berkeley, M.R. , Seppey, M. & Zdobnov, E.M. (2021) BUSCO: assessing genomic data quality and beyond. Current Protocols, 1, e323. 34936221 10.1002/cpz1.323

[tpj70426-bib-0062] Martin, M. (2011) Cutadapt removes adapter sequences from high‐throughput sequencing reads. EMBnet Journal, 17(3), 10–12.

[tpj70426-bib-0063] Mcclure, B. (2006) New views of S‐RNase‐based self‐incompatibility. Current Opinion in Plant Biology, 9, 639–646. 17027324 10.1016/j.pbi.2006.09.004

[tpj70426-bib-0064] Micol‐Ponce, R. , García‐Alcázar, M. , Lebrón, R. , Capel, C. , Pineda, B. , García‐Sogo, B. et al. (2022) Tomato POLLEN DEFICIENT 2 encodes a G‐type lectin receptor kinase required for viable pollen grain formation. Journal of Experimental Botany, 74, 178–193. 10.1093/jxb/erac419 PMC9786849 36260406

[tpj70426-bib-0065] Moreels, P. , Bigot, S. , Defalque, C. , Correa, F. , Martinez, J.P. , Lutts, S. et al. (2023) Intra‐and inter‐specific reproductive barriers in the tomato clade. Frontiers in Plant Science, 14, 1326689. 38143584 10.3389/fpls.2023.1326689 PMC10739309

[tpj70426-bib-0066] Muñoz‐Sanz, J.V. , TOVAR‐Méndez, A. , Lu, L. , Dai, R. & Mcclure, B. (2021) A cysteine‐rich protein, spDIR1L, implicated in S‐RNase‐independent pollen rejection in the tomato (Solanum section Lycopersicon) clade. International Journal of Molecular Sciences, 22(23), 13067. 34884871 10.3390/ijms222313067 PMC8657656

[tpj70426-bib-0067] Murfett, J. , Strabala, T.J. , Zurek, D.M. , Mou, B. , Beecher, B. & Mcclure, B.A. (1996) S RNase and interspecific pollen rejection in the genus nicotiana: multiple pollen‐rejection pathways contribute to unilateral incompatibility between self‐incompatible and self‐compatible species. The Plant Cell, 8(6), 943–958. 12239407 10.1105/tpc.8.6.943 PMC161150

[tpj70426-bib-0068] Ordonez, B. (2014) Pollen viability assessment.

[tpj70426-bib-0069] Paz, M.M. & Veilleux, R.E. (1999) Influence of culture medium and in vitro conditions on shoot regeneration in *Solanum phureja* monoploids and fertility of regenerated doubled monoploids. Plant Breeding, 118, 53–57.

[tpj70426-bib-0070] Peng, X. , Wang, M. , Li, Y. , Yan, W. , Chang, Z. , Chen, Z. et al. (2020) Lectin receptor kinase OsLecRK‐S.7 is required for pollen development and male fertility. Journal of Integrative Plant Biology, 62, 1227–1245. 31833176 10.1111/jipb.12897

[tpj70426-bib-0071] Pham, G.M. , Hamilton, J.P. , Wood, J.C. , Burke, J.T. , Zhao, H. , Vaillancourt, B. et al. (2020) Construction of a chromosome‐scale long‐read reference genome assembly for potato. GigaScience, 9(9), giaa100. 32964225 10.1093/gigascience/giaa100 PMC7509475

[tpj70426-bib-0072] Qin, X. & Chetelat, R.T. (2021) Ornithine decarboxylase genes contribute to S‐RNase‐independent pollen rejection. Plant Physiology, 186(1), 452–468. 33576789 10.1093/plphys/kiab062 PMC8154068

[tpj70426-bib-0073] Shen, W. , Le, S. , Li, Y. & Hu, F. (2016) SeqKit: a cross‐platform and ultrafast toolkit for FASTA/Q file manipulation. PLoS One, 11, e0163962. 27706213 10.1371/journal.pone.0163962 PMC5051824

[tpj70426-bib-0074] Shen, W. , Sipos, B. & Zhao, L. (2024) SeqKit2: a Swiss army knife for sequence and alignment processing. iMeta, 3, e191. 38898985 10.1002/imt2.191 PMC11183193

[tpj70426-bib-0075] Spooner, D.M.B. , Van Den Rodrigues, R.G. , Rodrigues, A. , Bamberg, J.B. , Hijmans, R.J. & Lara‐Cabrera, S. (2004) Wild potatoes (Solanum section Petota; Solanaceae) of north and Central America.

[tpj70426-bib-0076] Stamatakis, A. (2014) RAxML version 8: a tool for phylogenetic analysis and post‐analysis of large phylogenies. Bioinformatics, 30(9), 1312–1313. 24451623 10.1093/bioinformatics/btu033 PMC3998144

[tpj70426-bib-0077] Tarailo‐Graovac, M. & Chen, N. (2009) Using RepeatMasker to identify repetitive elements in genomic sequences. Current Protocols in Bioinformatics, 25, 4.10.1–4.10.14. 10.1002/0471250953.bi0410s25 19274634

[tpj70426-bib-0078] Tovar‐Méndez, A. , Kumar, A. , Kondo, K. , Ashford, A. , Baek, Y.S. , Welch, L. et al. (2014) Restoring pistil‐side self‐incompatibility factors recapitulates an interspecific reproductive barrier between tomato species. The Plant Journal, 77, 727–736. 24387692 10.1111/tpj.12424

[tpj70426-bib-0079] Tovar‐Méndez, A. , Lu, L. & Mcclure, B. (2017) HT proteins contribute to S‐RNase‐independent pollen rejection in Solanum. The Plant Journal, 89, 718–729. 27862494 10.1111/tpj.13416

[tpj70426-bib-0080] Vaillancourt, B. & Buell, C.R. (2019) High molecular weight DNA isolation method from diverse plant species for use with Oxford nanopore sequencing. *bioRxiv*, 783159.

[tpj70426-bib-0081] Van Ooijen, J.W. (2009) MapQTL® 6, Software for the mapping of quantitative trait loci in experimental populations of diploid species. Wageningen, Netherlands: Kyazma BV, 5.

[tpj70426-bib-0082] Wan, J. , Patel, A. , Mathieu, M. , Kim, S.‐Y. , Xu, D. & Stacey, G. (2008) A lectin receptor‐like kinase is required for pollen development in Arabidopsis. Plant Molecular Biology, 67, 469–482. 18392777 10.1007/s11103-008-9332-6

[tpj70426-bib-0083] Wood, D.E. , Lu, J. & Langmead, B. (2019) Improved metagenomic analysis with kraken 2. Genome Biology, 20, 257. 31779668 10.1186/s13059-019-1891-0 PMC6883579

[tpj70426-bib-0084] Yan, L.‐J. , Zhu, Z.‐G. , Wang, P. , Fu, C.‐N. , Guan, X.‐J. , Kear, P. et al. (2023) Comparative analysis of 343 plastid genomes of Solanum section Petota: insights into potato diversity, phylogeny, and species discrimination. Journal of Systematics and Evolution, 61, 599–612.

[tpj70426-bib-0085] Yermishin, A.P. , Polyukhovich, Y.V. , Voronkova, E.V. & Savchuk, A.V. (2014) Production of hybrids between the 2EBN bridge species *Solanum verrucosum* and 1EBN diploid potato species. American Journal of Potato Research, 91, 610–617.

[tpj70426-bib-0086] Zhang, M. , Fan, J. , Taylor, D.C. & Ohlrogge, J.B. (2009) DGAT1 and PDAT1 acyltransferases have overlapping functions in Arabidopsis triacylglycerol biosynthesis and are essential for normal pollen and seed development. The Plant Cell, 21(12), 3885–3901. 20040537 10.1105/tpc.109.071795 PMC2814504

[tpj70426-bib-0087] Zhang, S. , Wu, S. , Niu, C. , Liu, D. , Yan, T. , Tian, Y. et al. (2021) ZmMs25 encoding a plastid‐localized fatty acyl reductase is critical for anther and pollen development in maize. Journal of Experimental Botany, 72(12), 4298–4318. 33822021 10.1093/jxb/erab142

[tpj70426-bib-0088] Zhang, X. , Liu, Y. , Ayaz, A. , Zhao, H. & Lü, S. (2022) The plant fatty acyl reductases. International Journal of Molecular Sciences, 23(24), 16156. 36555796 10.3390/ijms232416156 PMC9783961

[tpj70426-bib-0089] Zhou, Q. , Tang, D. , Huang, W. , Yang, Z. , Zhang, Y. , Hamilton, J.P. et al. (2020) Haplotype‐resolved genome analyses of a heterozygous diploid potato. Nature Genetics, 52(10), 1018–1023. 32989320 10.1038/s41588-020-0699-x PMC7527274

