# Supplementary material for: Genetic basis for broad interspecific compatibility in Solanum verrucosum
Source: Plant J. 2025 Aug 21;123(4):e70426. doi: 10.1111/tpj.70426 (PMC12368477; doi:10.1111/tpj.70426)
Supplement: Supplementary file 2 — Figure S1. Behavior of S. pinnatisectum pollen tubes in the styles of the parents, F1, and F2 progeny. Figure S2. Phylogeny of S‐RNase CDS sequences from parents of the mapping population. Figure S3. Phylogeny of SLF sequences, showing the relationship of parental SLF sequences used in the mapping population. Figure S4. Mosaic plots showing the distribution of QTL haplotypes across phenotypic classes. Figure S5. Multiple sequence alignment of Soltu.DM.03G036770.1 and the DM1S1 and MSII1813‐2 orthologs. Figure S6. Gene tree of pectinacetylesterases from S. verrucosum MSII1813‐2, S. chacoense M6, S. tuberosum DM, S. tuberosum DM1S1, S. tuberosum RH, and Solanum lycopersicum M82 that was used as an outgroup. Figure S7. Multiple sequence alignment of Soltu.DM.09G000840.1 and the DM1S1 and MSII1813‐2 orthologs. Figure S8. Heat map of Log2 fold change of ui11.1 candidate gene Soltu.DM.11G021610.1 in the chromosome 11 QTL. Figure S9. Multiple sequence alignment of Soltu.DM.11G021610 and the DM1S1 and MSII1813‐2 orthologs. Table S1. Pollination phenotyping data for the F2 mapping population. Table S2. List of significant SNPs, their physical and map positions, LOD values, and nearest annotation on DM v6.1 assembly. Table S3. S‐RNase and SLF sequences from parents used in the mapping population used in the construction of phylogenies. Table S4. S‐RNase and SLF sequences used as comparison for the construction of phylogenies. Table S5. Transcripts per million (TPM) values for pollen expressed Solver.v1.03_VERG035240.1. Table S6. Genome assembly metrics for S. verrucosum MSII1813‐2. Table S7. Benchmarking universal single copy orthologs in the S. verrucosum MSII1813‐2 genome sequence and annotation. Table S8. Repetitive sequences identified in S. verrucosum MSII1813‐2. Table S9. Protein coding genes annotated in S. verrucosum MSII1813‐2. Table S10. Syntenic genes within the chromosome 11 QTL for ui11.1. Table S11. List of significantly differentially expressed genes (α = 0.01) [file TPJ-123-0-s002.zip › Manuscript_Supplemental_Tables_and_Figures.V8.2.docx]

Figure S1. Behavior of *S. pinnatisectum* pollen tubes in the styles of the parents, F1, and F2 progeny. Red arrows indicate where the longest pollen tube stops in the styles, or where pollen tubes are present in the ovary, as is the case for MSII1813-2 and MSJJ1821-049. The F2 MSJJ1821-041 is male sterile, failing to set fruit upon self-pollination, and inhibits the growth of *S. pinnatisectum* interspecific pollen tubes in the style. The F2 MSJJ1821-091 is male fertile setting abundant fruit after self-pollination, but also inhibits the growth of *S. pinnatisectum* pollen tubes. MSJJ1821F2-049 has the same phenotype as the *S. verrucosum* parent MSII1813-2, showing both strong male fertility and lack of interspecific reproductive barriers.

| Line | selfed flowers | selfed fruit | self seed | fruit per flower | total interspecific flowers | total interspecific fruit | total interspecific seed | pollen stainability | male fertility phenotypic class | interspecific compatibility phenotypic class |
| --- | --- | --- | --- | --- | --- | --- | --- | --- | --- | --- |
| MSJJ1821-01F2-001 | 12 | 9 | 1075 | 0.75 | 15 | 8 | 2 | 0.91 | MF | SvSC |
| MSJJ1821-01F2-002 | 12 | 2 | 0 | 0.17 | 15 | 0 | 0 | 0.1 |  |  |
| MSJJ1821-01F2-003 | 15 | 5 | 275 | 0.33 | 10 | 0 | 0 | 0.21 | MF | FIRB |
| MSJJ1821-01F2-004 | 15 | 0 | 0 | 0 | 14 | 0 | 0 | 0.73 | MS | FIRB |
| MSJJ1821-01F2-005 | 12 | 11 | 475 | 0.92 | 4 | 0 | 0 | 0.69 | MF |  |
| MSJJ1821-01F2-006 | 12 | 4 | 103 | 0.33 | 28 | 12 | 4 | 0.24 | MF | SvSC |
| MSJJ1821-01F2-007 | 14 | 9 | 525 | 0.64 | 18 | 0 | 0 | 0.69 | MF | FIRB |
| MSJJ1821-01F2-008 | 11 | 8 | 325 | 0.73 | 20 | 0 | 0 | 0.37 | MF | FIRB |
| MSJJ1821-01F2-009 | 12 | 0 | 0 | 0 | 0 | 0 | 0 | 0.87 | MS |  |
| MSJJ1821-01F2-010 |  |  |  |  | 0 | 0 | 0 |  |  |  |
| MSJJ1821-01F2-011 | 10 | 9 | 81 | 0.9 | 18 | 0 | 0 | 0.43 | MF | FIRB |
| MSJJ1821-01F2-012 | 13 | 5 | 100 | 0.38 | 16 | 0 | 0 | 0.43 | MF | FIRB |
| MSJJ1821-01F2-013 |  |  |  |  | 0 | 0 | 0 | 0.84 |  |  |
| MSJJ1821-01F2-014 | 15 | 8 | 650 | 0.53 | 16 | 0 | 0 | 0.78 | MF | FIRB |
| MSJJ1821-01F2-015 |  |  |  |  | 0 | 0 | 0 |  |  |  |
| MSJJ1821-01F2-016 | 17 | 9 | 575 | 0.53 | 18 | 0 | 0 | 0.77 | MF | FIRB |
| MSJJ1821-01F2-017 | 9 | 0 | 0 | 0 | 9 | 0 | 0 | 0.32 | MS | FIRB |
| MSJJ1821-01F2-018 | 10 | 9 | 1050 | 0.9 | 17 | 0 | 0 | 0.71 | MF | FIRB |
| MSJJ1821-01F2-019 | 11 | 6 | 22 | 0.55 | 17 | 0 | 0 | 0.63 | MF | FIRB |
| MSJJ1821-01F2-020 | 12 | 0 | 0 | 0 | 6 | 0 | 0 | 0.3 | MS | FIRB |
| MSJJ1821-01F2-021 | 9 | 3 | 65 | 0.33 | 9 | 0 | 0 | 0.45 | MF | FIRB |
| MSJJ1821-01F2-022 | 14 | 8 | 125 | 0.57 | 21 | 0 | 0 | 0.83 | MF | FIRB |
| MSJJ1821-01F2-023 | 12 | 0 | 0 | 0 | 5 | 0 | 0 | 0.01 |  |  |
| MSJJ1821-01F2-024 |  |  |  |  | 0 | 0 | 0 | 0.75 |  |  |
| MSJJ1821-01F2-025 |  |  |  |  | 0 | 0 | 0 | 0.47 |  |  |
| MSJJ1821-01F2-026 |  |  |  |  | 0 | 0 | 0 | 0.16 |  |  |
| MSJJ1821-01F2-027 | 18 | 12 | 900 | 0.67 | 11 | 0 | 0 | 0.79 | MF | FIRB |
| MSJJ1821-01F2-028 | 12 | 0 | 0 | 0 | 14 | 1 | 0 | 0.11 |  | FIRB |
| MSJJ1821-01F2-029 | 11 | 8 | 175 | 0.73 | 12 | 0 | 0 | 0.81 | MF | FIRB |
| MSJJ1821-01F2-030 | 4 | 2 | 0 | 0.5 | 0 | 0 | 0 | 0.85 |  |  |
| MSJJ1821-01F2-031 | 15 | 0 | 0 | 0 | 6 | 0 | 0 | 0.09 |  |  |
| MSJJ1821-01F2-032 | 12 | 2 | 28 | 0.17 | 16 | 0 | 0 | 0.8 |  | FIRB |
| MSJJ1821-01F2-033 | 10 | 4 | 14 | 0.4 | 17 | 0 | 0 | 0.61 | MF | FIRB |
| MSJJ1821-01F2-034 | 12 | 8 | 79 | 0.67 | 9 | 0 | 0 | 0.81 | MF | FIRB |
| MSJJ1821-01F2-035 | 10 | 0 | 0 | 0 | 0 | 0 | 0 | 0.35 |  |  |
| MSJJ1821-01F2-036 | 18 | 1 | 0 | 0.06 | 11 | 3 | 0 | 0.08 | MF | SvSC |
| MSJJ1821-01F2-037 |  |  |  |  | 0 | 0 | 0 | 0.37 |  |  |
| MSJJ1821-01F2-038 | 9 | 0 | 0 | 0 | 0 | 0 | 0 | 0.76 | MS |  |
| MSJJ1821-01F2-039 | 15 | 1 | 6 | 0.07 | 7 | 0 | 0 | 0.44 | MS | FIRB |
| MSJJ1821-01F2-040 | 13 | 11 | 260 | 0.85 | 15 | 0 | 0 | 0.48 | MF | FIRB |
| MSJJ1821-01F2-041 | 12 | 0 | 0 | 0 | 8 | 0 | 0 | 0.87 | MS | FIRB |
| MSJJ1821-01F2-042 |  |  |  |  | 0 | 0 | 0 |  |  |  |
| MSJJ1821-01F2-043 | 7 | 0 | 0 | 0 | 8 | 0 | 0 | 0.67 |  | FIRB |
| MSJJ1821-01F2-044 | 11 | 0 | 0 | 0 | 0 | 0 | 0 | 0.89 | MS |  |
| MSJJ1821-01F2-045 | 16 | 2 | 75 | 0.13 | 9 | 0 | 0 | 0.52 |  | FIRB |
| MSJJ1821-01F2-046 | 11 | 0 | 0 | 0 | 5 | 0 | 0 | 0.83 |  |  |
| MSJJ1821-01F2-047 | 13 | 9 | 600 | 0.69 | 18 | 0 | 0 | 0.88 | MF | FIRB |
| MSJJ1821-01F2-048 |  |  |  |  | 0 | 0 | 0 |  |  |  |
| MSJJ1821-01F2-049 | 12 | 8 | 150 | 0.67 | 24 | 23 | 5 | 0.74 | MF | SvSC |
| MSJJ1821-01F2-050 | 15 | 8 | 200 | 0.53 | 12 | 0 | 0 | 0.83 | MF | FIRB |
| MSJJ1821-01F2-051 | 17 | 0 | 0 | 0 | 0 | 0 | 0 | 0.12 |  |  |
| MSJJ1821-01F2-052 | 12 | 8 | 425 | 0.67 | 5 | 0 | 0 | 0.78 | MF |  |
| MSJJ1821-01F2-053 | 18 | 6 | 225 | 0.33 | 23 | 15 | 7 | 0.86 | MF | SvSC |
| MSJJ1821-01F2-054 | 14 | 1 | 2 | 0.07 | 0 | 0 | 0 | 0.79 | MS |  |
| MSJJ1821-01F2-055 | 12 | 0 | 0 | 0 | 0 | 0 | 0 | 0.5 | MS |  |
| MSJJ1821-01F2-056 |  |  |  |  | 0 | 0 | 0 |  |  |  |
| MSJJ1821-01F2-057 | 18 | 14 | 175 | 0.78 | 14 | 7 | 0 | 0.5 | MF | SvSC |
| MSJJ1821-01F2-058 | 4 | 0 | 0 | 0 | 0 | 0 | 0 | 0.25 |  |  |
| MSJJ1821-01F2-059 | 14 | 14 | 825 | 1 | 15 | 0 | 0 | 0.67 | MF | FIRB |
| MSJJ1821-01F2-060 | 9 | 6 | 350 | 0.67 | 3 | 0 | 0 | 0.71 | MF |  |
| MSJJ1821-01F2-061 |  |  |  |  | 0 | 0 | 0 |  |  |  |
| MSJJ1821-01F2-062 | 13 | 6 | 350 | 0.46 | 16 | 0 | 0 | 0.49 | MF | FIRB |
| MSJJ1821-01F2-063 | 13 | 1 | 25 | 0.08 | 6 | 0 | 0 | 0.74 | MS | FIRB |
| MSJJ1821-01F2-064 | 10 | 5 | 51 | 0.5 | 20 | 0 | 0 | 0.71 | MF | FIRB |
| MSJJ1821-01F2-065 | 3 | 0 | 0 | 0 | 0 | 0 | 0 | 0.81 |  |  |
| MSJJ1821-01F2-066 | 12 | 10 | 1000 | 0.83 | 12 | 0 | 0 | 0.78 | MF | FIRB |
| MSJJ1821-01F2-067 | 12 | 9 | 625 | 0.75 | 8 | 6 | 0 | 0.81 | MF | SvSC |
| MSJJ1821-01F2-068 | 13 | 0 | 0 | 0 | 0 | 0 | 0 | 0.69 | MS |  |
| MSJJ1821-01F2-069 | 17 | 1 | 3 | 0.06 | 12 | 0 | 0 | 0.84 | MS | FIRB |
| MSJJ1821-01F2-070 | 16 | 13 | 88 | 0.81 | 7 | 0 | 0 | 0.58 | MF | FIRB |
| MSJJ1821-01F2-071 | 15 | 1 | 13 | 0.07 | 8 | 0 | 0 | 0.81 | MS | FIRB |
| MSJJ1821-01F2-072 | 9 | 0 | 0 | 0 | 0 | 0 | 0 | 0.9 |  |  |
| MSJJ1821-01F2-073 | 10 | 0 | 0 | 0 | 5 | 0 | 0 | 0.19 |  |  |
| MSJJ1821-01F2-074 | 13 | 0 | 0 | 0 | 4 | 0 | 0 | 0.33 | MS |  |
| MSJJ1821-01F2-075 | 15 | 10 | 73 | 0.67 | 16 | 0 | 0 | 0.6 | MF | FIRB |
| MSJJ1821-01F2-076 | 10 | 0 | 0 | 0 | 0 | 0 | 0 | 0.41 | MS |  |
| MSJJ1821-01F2-077 | 11 | 0 | 0 | 0 | 0 | 0 | 0 | 0.5 | MS |  |
| MSJJ1821-01F2-078 | 18 | 0 | 0 | 0 | 9 | 0 | 0 | 0.66 | MS | FIRB |
| MSJJ1821-01F2-079 | 14 | 0 | 0 | 0 | 11 | 8 | 3 | 0.28 | MS | SvSC |
| MSJJ1821-01F2-080 | 11 | 10 | 250 | 0.91 | 7 | 2 | 0 | 0.87 | MF | SvSC |
| MSJJ1821-01F2-081 | 12 | 4 | 50 | 0.33 | 10 | 3 | 0 | 0.7 | MF |  |
| MSJJ1821-01F2-082 | 15 | 10 | 875 | 0.67 | 7 | 0 | 0 | 0.56 | MF | FIRB |
| MSJJ1821-01F2-083 |  |  |  |  | 0 | 0 | 0 | 0.74 |  |  |
| MSJJ1821-01F2-084 |  |  |  |  | 0 | 0 | 0 | 0.78 |  |  |
| MSJJ1821-01F2-085 | 15 | 5 | 20 | 0.33 | 17 | 0 | 0 | 0.33 | MF | FIRB |
| MSJJ1821-01F2-086 | 17 | 0 | 0 | 0 | 12 | 1 | 0 | 0.09 |  |  |
| MSJJ1821-01F2-087 | 3 | 0 | 0 | 0 | 0 | 0 | 0 | 0.35 |  |  |
| MSJJ1821-01F2-088 | 18 | 1 | 0 | 0.06 | 5 | 0 | 0 | 0.41 | MS |  |
| MSJJ1821-01F2-089 | 10 | 0 | 0 | 0 | 0 | 0 | 0 | 0.19 |  |  |
| MSJJ1821-01F2-090 | 9 | 8 | 325 | 0.89 | 13 | 0 | 0 | 0.52 | MF | FIRB |
| MSJJ1821-01F2-091 | 14 | 12 | 675 | 0.86 | 12 | 0 | 0 | 0.83 | MF | FIRB |
| MSJJ1821-01F2-092 | 10 | 0 | 0 | 0 | 0 | 0 | 0 | 0.41 | MS |  |
| MSJJ1821-01F2-093 | 13 | 11 | 465 | 0.85 | 16 | 8 | 0 | 0.66 | MF | SvSC |
| MSJJ1821-01F2-094 | 11 | 8 | 285 | 0.73 | 4 | 0 | 0 | 0.53 | MF |  |
| MSJJ1821-01F2-095 | 22 | 9 | 450 | 0.41 | 9 | 0 | 0 | 0.93 | MF | FIRB |
| MSJJ1821-01F2-096 | 15 | 9 | 225 | 0.6 | 13 | 0 | 0 | 0.75 | MF | FIRB |
| MSJJ1821-01F2-097 | 13 | 10 | 184 | 0.77 | 22 | 0 | 0 | 0.83 | MF | FIRB |
| MSJJ1821-01F2-098 | 18 | 3 | 0 | 0.17 | 0 | 0 | 0 | 0.84 | MS |  |
| MSJJ1821-01F2-099 | 12 | 9 | 650 | 0.75 | 19 | 6 | 1 | 0.59 | MF | SvSC |
| MSJJ1821-01F2-100 | 13 | 10 | 400 | 0.77 | 16 | 0 | 0 | 0.91 | MF | FIRB |
| MSJJ1821-01F2-101 | 14 | 4 | 125 | 0.29 | 7 | 0 | 0 | 0.73 | MF | FIRB |
| MSJJ1821-01F2-102 | 14 | 0 | 0 | 0 | 0 | 0 | 0 | 0.51 | MS |  |
| MSJJ1821-01F2-103 | 9 | 0 | 0 | 0 | 0 | 0 | 0 | 0.69 | MS |  |
| MSJJ1821-01F2-104 | 9 | 6 | 250 | 0.67 | 7 | 0 | 0 | 0.84 | MF | FIRB |
| MSJJ1821-01F2-105 | 13 | 11 | 108 | 0.85 | 15 | 0 | 0 | 0.78 | MF | FIRB |
| MSJJ1821-01F2-106 | 16 | 0 | 0 | 0 | 0 | 0 | 0 | 0.5 | MS |  |
| MSJJ1821-01F2-107 | 9 | 9 | 500 | 1 | 0 | 0 | 0 | 0.75 | MF |  |
| MSJJ1821-01F2-108 | 11 | 11 | 775 | 1 | 13 | 0 | 0 | 0.87 | MF | FIRB |
| MSJJ1821-01F2-109 | 10 | 2 | 0 | 0.2 | 29 | 5 | 0 | 0.83 |  |  |
| MSJJ1821-01F2-110 | 13 | 10 | 1275 | 0.77 | 13 | 4 | 0 | 0.94 | MF | SvSC |
| MSJJ1821-01F2-111 |  |  |  |  | 0 | 0 | 0 |  |  |  |
| MSJJ1821-01F2-112 | 16 | 0 | 0 | 0 | 5 | 0 | 0 | 0.23 | MS |  |
| MSJJ1821-01F2-113 | 13 | 0 | 0 | 0 | 0 | 0 | 0 | 0.55 | MS |  |
| MSJJ1821-01F2-114 | 5 | 0 | 0 | 0 | 0 | 0 | 0 | 0.62 |  |  |
| MSJJ1821-01F2-115 | 6 | 0 | 0 | 0 | 0 | 0 | 0 | 0.38 |  |  |
| MSJJ1821-01F2-116 | 5 | 0 | 0 | 0 | 0 | 0 | 0 | 0.67 |  |  |
| MSJJ1821-01F2-117 | 16 | 9 | 525 | 0.56 | 15 | 0 | 0 | 0.92 | MF | FIRB |
| MSJJ1821-01F2-118 | 16 | 1 | 8 | 0.06 | 4 | 0 | 0 | 0.65 | MS |  |
| MSJJ1821-01F2-119 | 7 | 0 | 0 | 0 | 0 | 0 | 0 | 0.05 |  |  |
| MSJJ1821-01F2-120 | 12 | 0 | 0 | 0 | 0 | 0 | 0 | 0.66 | MS |  |
| MSJJ1821-01F2-121 | 9 | 7 | 1125 | 0.78 | 10 | 4 | 0 | 0.48 | MF | SvSC |
| MSJJ1821-01F2-122 | 23 | 0 | 0 | 0 | 0 | 0 | 0 | 0.69 | MS |  |
| MSJJ1821-01F2-123 | 5 | 0 | 0 | 0 | 0 | 0 | 0 | 0.65 |  |  |
| MSJJ1821-01F2-124 | 7 | 0 | 0 | 0 | 0 | 0 | 0 | 0.34 |  |  |
| MSJJ1821-01F2-125 | 11 | 11 | 275 | 1 | 9 | 0 | 0 | 0.53 | MF | FIRB |
| MSJJ1821-01F2-126 | 15 | 0 | 0 | 0 | 0 | 0 | 0 | 0.53 | MS |  |
| MSJJ1821-01F2-127 | 13 | 12 | 150 | 0.92 | 12 | 0 | 0 | 0.44 | MF | FIRB |
| MSJJ1821-01F2-128 | 18 | 7 | 150 | 0.39 | 9 | 0 | 0 | 0.58 | MF | FIRB |
| MSJJ1821-01F2-129 | 18 | 13 | 30 | 0.72 | 15 | 0 | 0 | 0.63 | MF | FIRB |
| MSJJ1821-01F2-130 | 17 | 0 | 0 | 0 | 14 | 0 | 0 | 0.26 | MS | FIRB |
| MSJJ1821-01F2-131 | 9 | 0 | 0 | 0 | 0 | 0 | 0 | 0.64 | MS |  |
| MSJJ1821-01F2-132 | 16 | 0 | 0 | 0 | 0 | 0 | 0 | 0.6 | MS |  |
| MSJJ1821-01F2-133 |  |  |  |  | 0 | 0 | 0 |  |  |  |
| MSJJ1821-01F2-134 | 15 | 15 | 600 | 1 | 10 | 0 | 0 | 0.81 | MF | FIRB |
| MSJJ1821-01F2-135 | 2 | 0 | 0 | 0 | 0 | 0 | 0 | 0.63 |  |  |
| MSJJ1821-01F2-136 | 12 | 9 | 325 | 0.75 | 9 | 0 | 0 | 0.53 | MF | FIRB |
| MSJJ1821-01F2-137 | 19 | 10 | 67 | 0.53 | 34 | 12 | 3 | 0.6 | MF | SvSC |
| MSJJ1821-01F2-138 | 12 | 0 | 0 | 0 | 11 | 2 | 2 | 0.32 | MS | FIRB |
| MSJJ1821-01F2-139 | 11 | 4 | 81 | 0.36 | 10 | 0 | 0 | 0.53 | MF | FIRB |
| MSJJ1821-01F2-140 | 11 | 0 | 0 | 0 | 11 | 0 | 0 | 0.03 |  | FIRB |
| MSJJ1821-01F2-141 |  |  |  |  | 0 | 0 | 0 |  |  |  |
| MSJJ1821-01F2-142 | 11 | 6 | 230 | 0.55 | 12 | 0 | 0 | 0.62 | MF | FIRB |
| MSJJ1821-01F2-143 | 13 | 0 | 0 | 0 | 0 | 0 | 0 | 0.7 | MS |  |
| MSJJ1821-01F2-144 | 17 | 5 | 160 | 0.29 | 15 | 0 | 0 | 0.83 | MF | FIRB |
| MSJJ1821-01F2-145 | 13 | 0 | 0 | 0 | 0 | 0 | 0 | 0.57 | MS |  |
| MSJJ1821-01F2-146 |  |  |  |  | 0 | 0 | 0 |  |  |  |
| MSJJ1821-01F2-147 | 15 | 3 | 5 | 0.2 | 9 | 0 | 0 | 0.67 | MS | FIRB |
| MSJJ1821-01F2-148 | 13 | 5 | 269 | 0.38 | 11 | 0 | 0 | 0.72 | MF | FIRB |
| MSJJ1821-01F2-149 | 12 | 0 | 0 | 0 | 10 | 6 | 0 | 0.28 | MS | SvSC |
| MSJJ1821-01F2-150 | 18 | 18 | 1150 | 1 | 15 | 0 | 0 | 0.87 | MF | FIRB |

Table S1. Pollination phenotyping data for the F2 mapping population. For the phenotypic classes: MF = individuals that set fruit upon selfing indicating that they are male fertile, MS = represents individuals that are male sterile, SvSC = individuals that lack prezygotic IRBs against all potato species tested similar to *S. verrucosum* parent MSII1813-2, FIRB = individuals that have functional prezygotic IRBs similar to *S. tuberosum* parent DM1S1.

| SNP | Trait | Chromosome | Position DM 1-3 516 v4.03 (Mb) | Position DM 1-3 516 v6.1 (Mb) | Interval Map Position (cM) | LOD | LOD Threshold | % Expl. | Nearest Annotation |
| --- | --- | --- | --- | --- | --- | --- | --- | --- | --- |
| solcap_snp_c2_54811 | Interspecific Compatibility | chr01 | 46.3 | 53.0 | 33.0 | 4.2 | 4.2 | 21 | Soltu.DM.01G019390.1_enoyl-CoA hydratase/isomerase A |
| PotVar0095207 | Interspecific Compatibility | chr01 | 45.8 | 53.4 | 33.0 | 4.2 | 4.2 | 21 | Soltu.DM.01G019510.1_phytochrome B |
| ST4.03ch01_45174658 | Interspecific Compatibility | chr01 | 45.2 | 54.0 | 33.4 | 4.2 | 4.2 | 21 | Soltu.DM.01G019820.1_Glycosyl hydrolase superfamily protein |
| solcap_snp_c2_2653 | Interspecific Compatibility | chr01 | 58.7 | 60.4 | 41.8 | 4.3 | 4.2 | 21.5 | Soltu.DM.01G022300.1_Protein kinase superfamily protein |
| ST4.03ch01_58988173 | Interspecific Compatibility | chr01 | 59.0 | 60.6 | 42.2 | 4.3 | 4.2 | 21.5 | Soltu.DM.01G022450.1_Zinc finger C-x8-C-x5-C-x3-H type family protein |
| PotVar0014217 | Male Fertility | chr03 | 59.6 | 58.1 | 74.9 | 5.19 | 4 | 20.3 | Soltu.DM.03G034700.1_Late embryogenesis abundant (LEA) hydroxyproline-rich glycoprotein family |
| PotVar0021252 | Male Fertility | chr03 | 60.0 | 58.4 | 74.9 | 5.19 | 4 | 20.3 | Soltu.DM.03G035070.1_Pyridoxal phosphate (PLP)-dependent transferases superfamily protein |
| ST4.03ch03_59967862 | Male Fertility | chr03 | 60.0 | 58.4 | 74.9 | 5.19 | 4 | 20.3 | Soltu.DM.03G035090.1_mitochondrial RNAediting factor |
| ST4.03ch03_59989747 | Male Fertility | chr03 | 60.0 | 58.4 | 74.9 | 5.19 | 4 | 20.3 | Soltu.DM.03G035140.1_tRNAse Z4 |
| ST4.03ch03_60088636 | Male Fertility | chr03 | 60.1 | 58.5 | 75.6 | 4.68 | 4 | 18.6 | Soltu.DM.03G035270.2_Major facilitator superfamily protein |
| ST4.03ch03_60234489 | Male Fertility | chr03 | 60.2 | 58.7 | 76.3 | 5.84 | 4 | 22.6 | Soltu.DM.03G035510.1_ATP binding microtubule motor family protein |
| ST4.03ch03_60355453 | Male Fertility | chr03 | 60.4 | 58.8 | 76.3 | 5.84 | 4 | 22.6 | Soltu.DM.03G035640.1_Homeobox-leucine zipper family protein / lipid-binding START domain-containing protein |
| PotVar0021182 | Male Fertility | chr03 | 60.5 | 58.9 | 76.9 | 6.46 | 4 | 24.7 | Soltu.DM.03G035790.1_2-oxoglutarate (2OG) and Fe(II)-dependent oxygenase superfamily protein |
| PotVar0021180 | Male Fertility | chr03 | 60.5 | 58.9 | 76.9 | 6.46 | 4 | 24.7 | Soltu.DM.03G035790.1_2-oxoglutarate (2OG) and Fe(II)-dependent oxygenase superfamily protein |
| ST4.03ch03_60633387 | Male Fertility | chr03 | 60.6 | 59.0 | 78.3 | 7.16 | 4 | 26.9 | Soltu.DM.03G035970.3_basic helix-loop-helix (bHLH) DNA-binding superfamily protein |
| PotVar0021019 | Male Fertility | chr03 | 60.7 | 59.1 | 78.3 | 7.16 | 4 | 26.9 | Soltu.DM.03G035970.3_basic helix-loop-helix (bHLH) DNA-binding superfamily protein |
| PotVar0020948 | Male Fertility | chr03 | 60.8 | 59.3 | 78.7 | 7.16 | 4 | 26.9 | Soltu.DM.03G036220.1_RNA-binding (RRM/RBD/RNP motifs) family protein |
| ST4.03ch03_60862996 | Male Fertility | chr03 | 60.9 | 59.3 | 78.7 | 7.16 | 4 | 26.9 | Soltu.DM.03G036250.1_atypical CYS HIS rich thioredoxin |
| solcap_snp_c2_9497 | Male Fertility | chr03 | 60.9 | 59.3 | 78.7 | 7.16 | 4 | 26.9 | Soltu.DM.03G036320.1_beta galactosidase |
| ST4.03ch03_60952639 | Male Fertility | chr03 | 61.0 | 59.4 | 78.7 | 7.16 | 4 | 26.9 | Soltu.DM.03G036350.1_transmembrane nine |
| PotVar0020829 | Male Fertility | chr03 | 61.2 | 59.6 | 79.0 | 7.2 | 4 | 27.1 | Soltu.DM.03G036720.1_Pyridoxal-5'-phosphate-dependent enzyme family protein |
| ST4.03ch03_61233807 | Male Fertility | chr03 | 61.2 | 59.6 | 79.0 | 7.2 | 4 | 27.1 | Soltu.DM.03G036720.1_Pyridoxal-5'-phosphate-dependent enzyme family protein |
| ST4.03ch03_61342320 | Male Fertility | chr03 | 61.3 | 59.7 | 79.0 | 7.2 | 4 | 27.1 | Soltu.DM.03G036810.1_Transcription initiation factor TFIID subunit A |
| PotVar0020543 | Male Fertility | chr03 | 61.4 | 59.8 | 79.3 | 7.16 | 4 | 26.9 | Soltu.DM.03G036950.1_Aldolase-type TIM barrel family protein |
| PotVar0020457 | Male Fertility | chr03 | 61.5 | 59.9 | 79.7 | 7.13 | 4 | 26.9 | Soltu.DM.03G037040.2_Mono-/di-acylglycerol lipase, N-terminal;Lipase, class |
| PotVar0020194 | Male Fertility | chr03 | 61.6 | 60.0 | 79.7 | 7.13 | 4 | 26.9 | Soltu.DM.03G037170.1_RELA/SPOT homolog |
| PotVar0020171 | Male Fertility | chr03 | 61.6 | 60.0 | 79.7 | 7.13 | 4 | 26.9 | Soltu.DM.03G037180.1_hypothetical protein |
| ST4.03ch03_61682469 | Male Fertility | chr03 | 61.7 | 60.1 | 80.0 | 7.11 | 4 | 26.8 | Soltu.DM.03G037290.1_related to AP2 |
| PotVar0020037 | Male Fertility | chr03 | 61.9 | 60.3 | 80.7 | 7.1 | 4 | 26.7 | Soltu.DM.03G037570.1_cation exchanger |
| PotVar0019900 | Male Fertility | chr03 | 61.9 | 60.3 | 80.7 | 7.1 | 4 | 26.7 | Soltu.DM.03G037580.1_MAP kinase kinase |
| PotVar0019861 | Male Fertility | chr03 | 62.0 | 60.3 | 80.7 | 7.1 | 4 | 26.7 | Soltu.DM.03G037660.1_exocyst complex component 84B |
| solcap_snp_c2_18873 | Male Fertility | chr08 | 42.7 | 45.3 | 25.3 | 4.02 | 4 | 16.1 | Soltu.DM.08G017040.1_conserved hypothetical protein |
| PotVar0086598 | Male Fertility | chr08 | 43.1 | 45.7 | 25.7 | 4.13 | 4 | 16.6 | Soltu.DM.08G017330.1_P-loop containing nucleoside triphosphate hydrolases superfamily protein |
| PotVar0086907 | Male Fertility | chr08 | 43.1 | 45.7 | 25.7 | 4.13 | 4 | 16.6 | Soltu.DM.08G017360.1_Tetratricopeptide repeat (TPR)-like superfamily protein |
| ST4.03ch08_43897919 | Male Fertility | chr08 | 43.9 | 46.4 | 26.0 | 4.13 | 4 | 16.6 | Soltu.DM.08G017850.1_hypothetical protein |
| ST4.03ch08_44746653 | Male Fertility | chr08 | 44.7 | 47.2 | 28.1 | 5.16 | 4 | 20.2 | Soltu.DM.08G018360.1_Magnesium transporter CorA-like family protein |
| ST4.03ch08_44861743 | Male Fertility | chr08 | 44.9 | 47.3 | 28.4 | 5.16 | 4 | 20.2 | Soltu.DM.08G018510.1_rhamnose biosynthesis |
| PotVar0077006 | Male Fertility | chr08 | 45.0 | 47.4 | 29.1 | 5.16 | 4 | 20.2 | Soltu.DM.08G018670.1_arabinose kinase |
| PotVar0077179 | Male Fertility | chr08 | 45.0 | 47.4 | 29.1 | 5.16 | 4 | 20.2 | Soltu.DM.08G018680.1_AIG2-like (avirulence induced gene) family protein |
| solcap_snp_c2_15796 | Male Fertility | chr08 | 45.4 | 47.8 | 29.5 | 5.65 | 4 | 22 | Soltu.DM.08G018980.1_DNA binding;ATP binding;nucleic acid binding;binding;helicases;ATP binding;DNA binding;helicases |
| solcap_snp_c2_44273 | Male Fertility | chr08 | 45.5 | 47.9 | 29.8 | 6.65 | 4 | 25.3 | Soltu.DM.08G019050.1_repressor of lrx1 |
| PotVar0077501 | Male Fertility | chr08 | 45.6 | 48.1 | 30.1 | 6.9 | 4 | 26.1 | Soltu.DM.08G019140.1_Protein of unknown function (DUF_B2219) domain containing protein |
| PotVar0125381 | Male Fertility | chr08 | 45.9 | 48.3 | 30.5 | 6.9 | 4 | 26.1 | Soltu.DM.08G019300.1_Protein of unknown function (DUF_B2219) domain containing protein |
| solcap_snp_c1_14763 | Male Fertility | chr08 | 46.0 | 48.4 | 30.5 | 6.9 | 4 | 26.1 | Soltu.DM.08G019430.1_Protein of Unknown Function (DUF239) |
| solcap_snp_c2_51053 | Male Fertility | chr08 | 46.4 | 48.7 | 30.8 | 7.01 | 4 | 26.5 | Soltu.DM.08G019790.1_DNAse I-like superfamily protein |
| PotVar0103193 | Male Fertility | chr08 | 47.2 | 49.6 | 31.9 | 6.49 | 4 | 24.8 | Soltu.DM.08G020170.1_xyloglucanase |
| ST4.03ch08_47200877 | Male Fertility | chr08 | 47.2 | 49.6 | 32.2 | 6.2 | 4 | 23.8 | Soltu.DM.08G020220.1_hypothetical protein |
| PotVar0103305 | Male Fertility | chr08 | 47.4 | 49.8 | 32.9 | 5.71 | 4 | 22.1 | Soltu.DM.08G020370.1_nine-cis-epoxycarotenoid dioxygenase |
| PotVar0103335 | Male Fertility | chr08 | 47.4 | 49.8 | 32.9 | 5.71 | 4 | 22.1 | Soltu.DM.08G020370.1_nine-cis-epoxycarotenoid dioxygenase |
| solcap_snp_c2_28641 | Male Fertility | chr08 | 48.0 | 50.6 | 33.6 | 5.24 | 4 | 20.5 | Soltu.DM.08G021150.1_Amino acid permease family protein |
| solcap_snp_c2_28521 | Male Fertility | chr08 | 48.1 | 50.7 | 33.9 | 4.98 | 4 | 19.6 | Soltu.DM.08G021200.1_CLP protease P4 |
| solcap_snp_c2_28538 | Male Fertility | chr08 | 48.2 | 50.7 | 34.9 | 4.53 | 4 | 18 | Soltu.DM.08G021310.1_KOW domain-containing protein |
| solcap_snp_c2_28572 | Male Fertility | chr08 | 48.3 | 50.9 | 34.9 | 4.53 | 4 | 18 | Soltu.DM.08G021510.1_growth-regulating factor |
| solcap_snp_c2_28635 | Male Fertility | chr08 | 48.7 | 51.2 | 35.3 | 4.63 | 4 | 18.4 | Soltu.DM.08G021820.1_Protein of unknown function (DUF760) |
| solcap_snp_c2_28637 | Male Fertility | chr08 | 48.7 | 51.2 | 35.3 | 4.63 | 4 | 18.4 | Soltu.DM.08G021820.1_Protein of unknown function (DUF760) |
| ST4.03ch08_48748042 | Male Fertility | chr08 | 48.7 | 51.3 | 35.3 | 4.63 | 4 | 18.4 | Soltu.DM.08G021930.1_conserved hypothetical protein |
| ST4.03ch08_48754700 | Male Fertility | chr08 | 48.8 | 51.3 | 35.3 | 4.63 | 4 | 18.4 | Soltu.DM.08G021940.1_protein dimerizations |
| solcap_snp_c2_49354 | Male Fertility | chr08 | 48.9 | 51.4 | 35.6 | 4.53 | 4 | 18 | Soltu.DM.08G022100.1_early-responsive to dehydration stress protein (ERD4) |
| solcap_snp_c1_2614 | Male Fertility | chr08 | 48.9 | 51.5 | 35.6 | 4.53 | 4 | 18 | Soltu.DM.08G022120.1_alpha/beta-Hydrolases superfamily protein |
| PotVar0063446 | Male Fertility | chr08 | 48.9 | 51.5 | 36.0 | 4.31 | 4 | 17.2 | Soltu.DM.08G022130.1_gamma-tocopherol methyltransferase |
| PotVar0063427 | Male Fertility | chr08 | 48.9 | 51.5 | 36.0 | 4.31 | 4 | 17.2 | Soltu.DM.08G022130.1_gamma-tocopherol methyltransferase |
| ST4.03ch08_49061858 | Male Fertility | chr08 | 49.1 | 51.6 | 36.3 | 4.31 | 4 | 17.2 | Soltu.DM.08G022210.1_shikimate kinase like |
| solcap_snp_c2_44545 | Male Fertility | chr08 | 49.1 | 51.6 | 36.3 | 4.31 | 4 | 17.2 | Soltu.DM.08G022230.1_hypothetical protein |
| solcap_snp_c2_44472 | Male Fertility | chr08 | 49.1 | 51.6 | 36.3 | 4.31 | 4 | 17.2 | Soltu.DM.08G022250.1_NAD(P)-binding Rossmann-fold superfamily protein |
| PotVar0063169 | Male Fertility | chr08 | 49.2 | 51.7 | 36.3 | 4.31 | 4 | 17.2 | Soltu.DM.08G022360.1_Syntaxin/t-SNARE family protein |
| solcap_snp_c2_19080 | Male Fertility | chr08 | 52.5 | 55.0 | 41.4 | 4.07 | 4 | 16.3 | Soltu.DM.08G025160.1_Lactate/malate dehydrogenase family protein |
| solcap_snp_c2_19079 | Male Fertility | chr08 | 52.5 | 55.0 | 41.4 | 4.07 | 4 | 16.3 | Soltu.DM.08G025160.1_Lactate/malate dehydrogenase family protein |
| solcap_snp_c2_19078 | Male Fertility | chr08 | 52.5 | 55.0 | 41.4 | 4.07 | 4 | 16.3 | Soltu.DM.08G025160.1_Lactate/malate dehydrogenase family protein |
| ST4.03ch09_585190 | Male Fertility | chr09 | 0.6 | 0.6 | 6.0 | 4.04 | 4 | 16.2 | Soltu.DM.09G000810.1_receptor lectin kinase |
| solcap_snp_c2_36580 | Interspecific Compatibility | chr11 | 32.2 | 34.1 | 33.2 | 4.68 | 4.2 | 23.1 | Soltu.DM.11G017490_AGAMOUS-like (4) |
| PotVar0071276 | Interspecific Compatibility | chr11 | 34.1 | 36.0 | 33.6 | 4.77 | 4.2 | 23.5 | Soltu.DM.11G017990.1_MSF1-like family protein (2) |
| solcap_snp_c2_31428 | Interspecific Compatibility | chr11 | 34.6 | 36.5 | 33.9 | 4.77 | 4.2 | 23.5 | Soltu.DM.11G018200.1_heavy metal atpase |
| solcap_snp_c1_4359 | Interspecific Compatibility | chr11 | 35.7 | 37.6 | 34.6 | 4.59 | 4.2 | 22.7 | Soltu.DM.11G018860.1_RING/U-box superfamily protein |
| solcap_snp_c1_4378 | Interspecific Compatibility | chr11 | 36.2 | 38.1 | 34.9 | 4.59 | 4.2 | 22.7 | Soltu.DM.11G019140.1_Aldolase superfamily protein |
| solcap_snp_c1_4384 | Interspecific Compatibility | chr11 | 36.3 | 38.1 | 35.3 | 4.68 | 4.2 | 23.1 | Soltu.DM.11G019160.2_Protein of unknown function (DUF803) |
| ST4.03ch11_38047038 | Interspecific Compatibility | chr11 | 38.0 | 39.7 | 36.0 | 4.68 | 4.2 | 23.1 | Soltu.DM.11G020010.1_Thymidine kinase |
| solcap_snp_c2_14947 | Interspecific Compatibility | chr11 | 38.2 | 39.9 | 36.3 | 4.68 | 4.2 | 23.1 | Soltu.DM.11G020180.1_xanthine dehydrogenase |
| ST4.03ch11_39205183 | Interspecific Compatibility | chr11 | 39.2 | 40.8 | 39.0 | 7.2 | 4.2 | 33.3 | Soltu.DM.11G020820.3_Domain of unknown function (DUF966) |
| ST4.03ch11_39617800 | Interspecific Compatibility | chr11 | 39.6 | 41.2 | 39.4 | 7.2 | 4.2 | 33.3 | Soltu.DM.11G021150.1_Phototropic-responsive NPH3 family protein |
| ST4.03ch11_39723410 | Interspecific Compatibility | chr11 | 39.7 | 41.3 | 39.7 | 6.23 | 4.2 | 29.5 | Soltu.DM.11G021210.1_cellulose synthase-like A02 |
| PotVar0047371 | Interspecific Compatibility | chr11 | 39.8 | 41.3 | 40.1 | 6.18 | 4.2 | 29.3 | Soltu.DM.11G021280.1_arogenate dehydratase |
| ST4.03ch11_40111293 | Interspecific Compatibility | chr11 | 40.1 | 41.7 | 40.7 | 7.46 | 4.2 | 34.2 | Soltu.DM.11G021610.1_Jojoba acyl CoA reductase-related male sterility protein |
| PotVar0112839 | Interspecific Compatibility | chr11 | 40.5 | 42.0 | 41.1 | 7.46 | 4.2 | 34.2 | Soltu.DM.11G021960.1_nitrilase-like protein |
| PotVar0112395 | Interspecific Compatibility | chr11 | 40.6 | 42.1 | 41.4 | 7.46 | 4.2 | 34.2 | Soltu.DM.11G022150.1_methyl-CPG-binding domain |
| solcap_snp_c2_22182 | Interspecific Compatibility | chr11 | 40.8 | 42.3 | 42.5 | 7.42 | 4.2 | 34.1 | Soltu.DM.11G022320.1_BEL1-like homeodomain |
| PotVar0112205 | Interspecific Compatibility | chr11 | 40.9 | 42.3 | 43.1 | 7.39 | 4.2 | 34 | Soltu.DM.11G022370.1_TCP-1/cpn60 chaperonin family protein |
| solcap_snp_c2_22219 | Interspecific Compatibility | chr11 | 40.9 | 42.4 | 43.5 | 7.39 | 4.2 | 34 | Soltu.DM.11G022390.1_NB-ARC domain-containing disease resistance protein |
| solcap_snp_c1_6972 | Interspecific Compatibility | chr11 | 41.1 | 42.5 | 43.8 | 6.03 | 4.2 | 28.7 | Soltu.DM.11G022530.1_20S proteasome beta subunit G1 |
| solcap_snp_c2_15420 | Interspecific Compatibility | chr11 | 41.2 | 42.7 | 44.2 | 6.03 | 4.2 | 28.7 | Soltu.DM.11G022680.1_Galactose oxidase/kelch repeat superfamily protein |
| PotVar0008207 | Interspecific Compatibility | chr11 | 41.5 | 42.9 | 44.5 | 6.08 | 4.2 | 28.9 | Soltu.DM.11G022880.1_Protein kinase superfamily protein |
| solcap_snp_c2_15336 | Interspecific Compatibility | chr11 | 41.6 | 43.0 | 45.9 | 6.58 | 4.2 | 30.9 | Soltu.DM.11G022970.1_auxin response factor |
| solcap_snp_c2_15314 | Interspecific Compatibility | chr11 | 41.7 | 43.1 | 46.2 | 6.58 | 4.2 | 30.9 | Soltu.DM.11G023020.1_Protein of unknown function, DUF584 |
| PotVar0008263 | Interspecific Compatibility | chr11 | 41.8 | 43.3 | 46.6 | 6.58 | 4.2 | 30.9 | Soltu.DM.11G023180.1_allene oxide synthase |
| ST4.03ch11_41899809 | Interspecific Compatibility | chr11 | 41.9 | 43.3 | 46.6 | 6.57 | 4.2 | 30.9 | Soltu.DM.11G023210.1_5'-AMP-activated protein kinase beta-2 subunit protein |
| solcap_snp_c2_15257 | Interspecific Compatibility | chr11 | 42.0 | 43.4 | 46.9 | 6.52 | 4.2 | 30.6 | Soltu.DM.11G023280.1_ABC-2 type transporter family protein |
| ST4.03ch11_41999792 | Interspecific Compatibility | chr11 | 42.0 | 43.4 | 46.9 | 6.52 | 4.2 | 30.6 | Soltu.DM.11G023280.1_ABC-2 type transporter family protein |
| solcap_snp_c2_15268 | Interspecific Compatibility | chr11 | 42.3 | 43.7 | 47.2 | 6.52 | 4.2 | 30.6 | Soltu.DM.11G023510.1_FTSH protease |
| PotVar0008494 | Interspecific Compatibility | chr11 | 42.6 | 44.1 | 47.6 | 5.39 | 4.2 | 26.1 | Soltu.DM.11G023900.1_peroxin4 |
| ST4.03ch11_42906980 | Interspecific Compatibility | chr11 | 42.9 | 44.4 | 49.3 | 4.61 | 4.2 | 22.8 | Soltu.DM.11G024290.1_hydroxycinnamoyl-CoA shikimate/quinate hydroxycinnamoyl transferase |
| ST4.03ch11_43028227 | Interspecific Compatibility | chr11 | 43.0 | 44.5 | 49.6 | 4.61 | 4.2 | 22.8 | Soltu.DM.11G024440.1-aldehyde oxidase |
| solcap_snp_c1_11826 | Interspecific Compatibility | chr11 | 43.1 | 44.6 | 50.0 | 4.61 | 4.2 | 22.8 | Soltu.DM.11G024500.1_NF-kappa-B-activating protein C-terminal domain containing protein |
| ST4.03ch11_43347339 | Interspecific Compatibility | chr11 | 43.3 | 44.8 | 51.0 | 4.61 | 4.2 | 22.8 | Soltu.DM.11G024760.1_P-loop containing nucleoside triphosphate hydrolases superfamily protein |
| solcap_snp_c2_31568 | Interspecific Compatibility | chr11 | 43.6 | 45.1 | 52.4 | 4.49 | 4.2 | 22.3 | Soltu.DM.11G025020.1_Bifunctional inhibitor/lipid-transfer protein/seed storage 2S albumin superfamily protein |
| PotVar0008637 | Interspecific Compatibility | chr11 | 43.8 | 45.3 | 52.4 | 4.49 | 4.2 | 22.3 | Soltu.DM.11G025230.1_2-oxoglutarate (2OG) and Fe(II)-dependent oxygenase superfamily protein |
| PotVar0008826 | Interspecific Compatibility | chr11 | 43.9 | 45.3 | 52.7 | 4.43 | 4.2 | 22 | Soltu.DM.11G025350.1_conserved hypothetical protein |
| PotVar0008851 | Interspecific Compatibility | chr11 | 43.9 | 45.3 | 52.7 | 4.43 | 4.2 | 22 | Soltu.DM.11G025350.1_conserved hypothetical protein |
| PotVar0130427 | Interspecific Compatibility | chr11 | 44.3 | 45.7 | 54.1 | 4.49 | 4.2 | 22.3 | Soltu.DM.11G025790.1_NAD(P)-binding Rossmann-fold superfamily protein |
| PotVar0130495 | Interspecific Compatibility | chr11 | 44.4 | 45.8 | 54.1 | 4.49 | 4.2 | 22.3 | Soltu.DM.11G025890.1_related to AP2.7 |
| ST4.03ch11_44646152 | Interspecific Compatibility | chr11 | 44.6 | 46.0 | 54.4 | 4.49 | 4.2 | 22.3 | Soltu.DM.11G026010.1_Protein kinase superfamily protein |
| PotVar0124363 | Interspecific Compatibility | chr11 | 44.6 | 46.0 | 54.4 | 4.49 | 4.2 | 22.3 | Soltu.DM.11G026010.1_Protein kinase superfamily protein |

Table S2. List of significant SNPs, their physical and map positions, LOD values, and nearest annotation on DM v6.1 assembly.


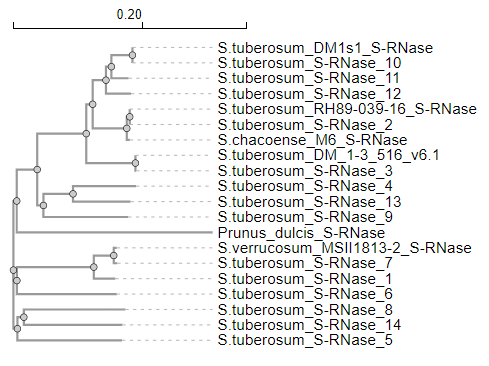


Figure S2. Phylogeny of *S-RNase* CDS sequences from parents of mapping population. The commonly referenced clones DM 1-3 516, RH89-039-16, M6, and *S. tuberosum* *S-RNase* sequences from Ma et al (2021) are included as comparisons. Figure created using Clustal Omega (Madeira et al. 2022).


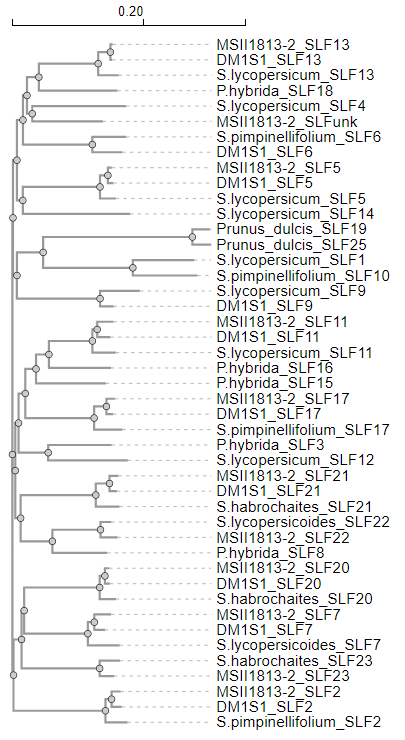


Figure S3. Phylogeny of *SLF* sequences, showing relationship of parental *SLF* sequences used in mapping population. Figure created using Clustal Omega (Madeira et al. 2022).

| Name | Gene Name | Genome Assembly | Position on chromosome 1 |
| --- | --- | --- | --- |
| S.tuberosum_DM1S1_S-RNase | Soltu.DM1S1.01G015930.1 | DM1S1 v1 | 37325138-37325862 |
| S.verrucosum_MSII1813-2_S-RNase | Solver.v1.01_VERG042990.1 | VER v1 | 33839524-33840302 |
| S.tuberosum_DM1S1_SLF2 | NA | DM1S1 v1 | 41441486-41440062 |
| S.tuberosum_DM1S1_SLF5 | NA | DM1S1 v1 | 41546341-41545172 |
| S.tuberosum_DM1S1_SLF6 | NA | DM1S1 v1 | 45765739-45764615 |
| S.tuberosum_DM1S1_SLF7 | NA | DM1S1 v1 | 41746745-41745576 |
| S.tuberosum_DM1S1_SLF9 | NA | DM1S1 v1 | 43428396-43427346 |
| S.tuberosum_DM1S1_SLF11 | NA | DM1S1 v1 | 45051018-45052179 |
| S.tuberosum_DM1S1_SLF13 | NA | DM1S1 v1 | 46345248-46344046 |
| S.tuberosum_DM1S1_SLF17 | NA | DM1S1 v1 | 39802967-39801820 |
| S.tuberosum_DM1S1_SLF20 | NA | DM1S1 v1 | 41833415-41832249 |
| S.tuberosum_DM1S1_SLF21 | NA | DM1S1 v1 | 42197316-42196008 |
| S.verrucosum_MSII1813-2_SLF2 | Solver.v1.01_VERG042790.1 | VER v1 | 32722880-32724070 |
| S.verrucosum_MSII1813-2_SLF5 | Solver.v1.01_VERG058430.1 | VER v1 | 32722880-32724070 |
| S.verrucosum_MSII1813-2_SLF7 | Solver.v1.01_VERG058460.1 | VER v1 | 42625635_42626507 |
| S.verrucosum_MSII1813-2_SLF11 | Solver.v1.01_VERG059550.1 | VER v1 | 46391374-46392075 |
| S.verrucosum_MSII1813-2_SLF13 | Solver.v1.01_VERG060660.1 | VER v1 | 49070336-49071538 |
| S.verrucosum_MSII1813-2_SLF17 | Solver.v1.01_VERG042760.1 | VER v1 | 32653330-32654262 |
| S.verrucosum_MSII1813-2_SLF20 | Solver.v1.01_VERG058480.1 | VER v1 | 42705188-42706276 |
| S.verrucosum_MSII1813-2_SLF21 | Solver.v1.01_VERG058650.1 | VER v1 | 43296637-43296657 |
| S.verrucosum_MSII1813-2_SLF22 | Solver.v1.01_VERG042560.2 | VER v1 | 31695445-31695731 |
| S.verrucosum_MSII1813-2_SLF23 | Solver.v1.01_VERG042750.1 | VER v1 | 32574635-32575810 |
| S.verrucosum_MSII1813-2_SLFunk | Solver.v1.01_VERG058760.1 | VER v1 | 44305792-44306970 |

Table S3. *S-RNase* and *SLF* sequences from parents used in mapping population used in construction of phylogenies (Supplemental Figures 2 and 3). Loci name listed in the first column with the annotation name in the second column, no SLFs are annotated on the DM1S1 assembly. Position on the parental genome is listed in the fourth column.


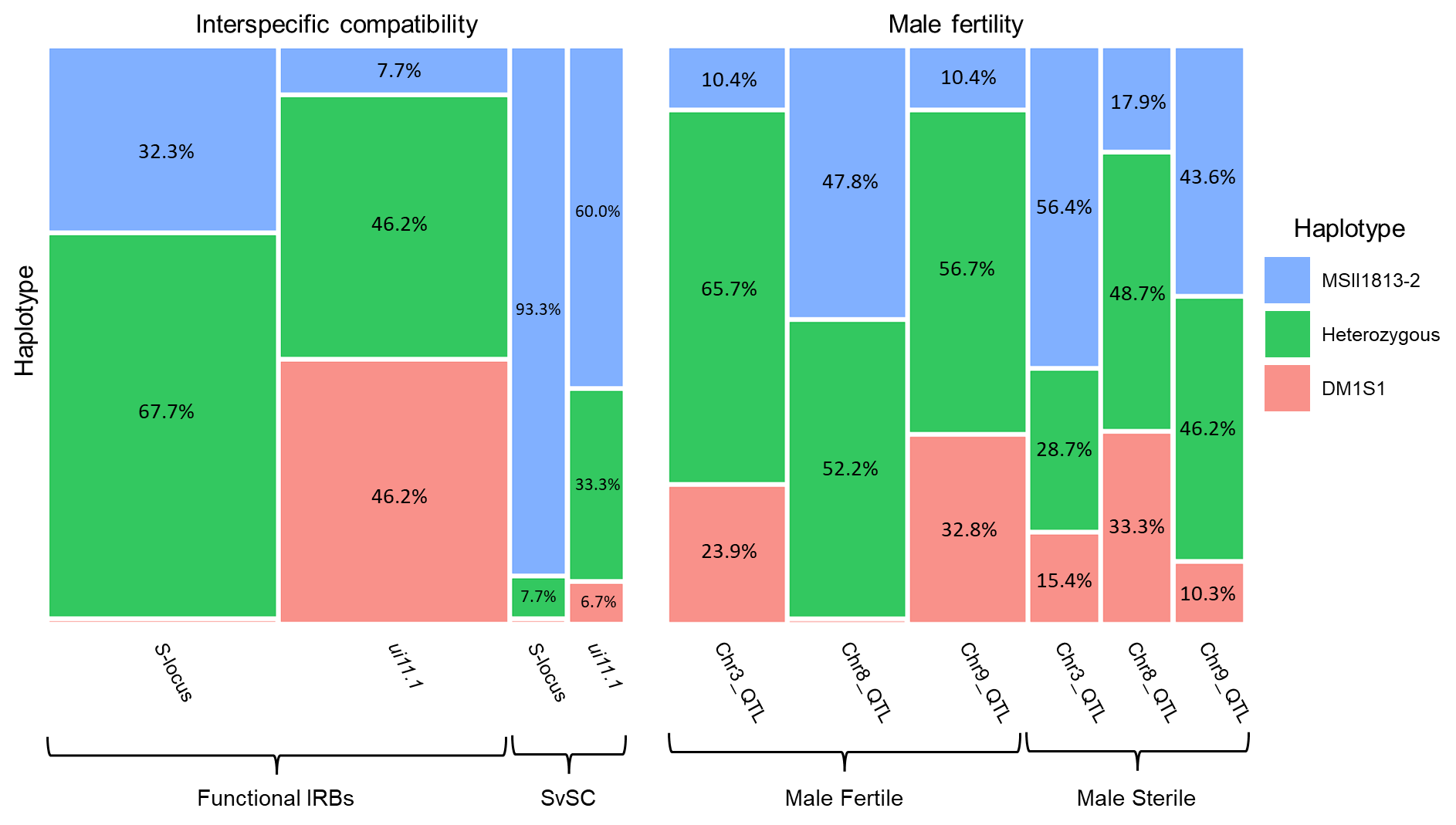


Figure S4. Mosaic plots showing distribution of haplotypes across phenotypic classes. Calculations were made using the most significant SNPs from the QTL analysis.

| Name | Genbank ID | Species |
| --- | --- | --- |
| S.tuberosum_S-RNase_1 | MZ561404.1 | *Solanum tuberosum* |
| S.tuberosum_S-RNase_2 | MZ561405.1 | *Solanum tuberosum* |
| S.tuberosum_S-RNase_3 | MZ561406.1 | *Solanum tuberosum* |
| S.tuberosum_S-RNase_4 | MZ561407.1 | *Solanum tuberosum* |
| S.tuberosum_S-RNase_5 | MZ561408.1 | *Solanum tuberosum* |
| S.tuberosum_S-RNase_6 | MZ561409.1 | *Solanum tuberosum* |
| S.tuberosum_S-RNase_7 | MZ561410.1 | *Solanum tuberosum* |
| S.tuberosum_S-RNase_8 | MZ561411.1 | *Solanum tuberosum* |
| S.tuberosum_S-RNase_9 | MZ561412.1 | *Solanum tuberosum* |
| S.tuberosum_S-RNase_10 | MZ561413.1 | *Solanum tuberosum* |
| S.tuberosum_S-RNase_11 | MZ561414.1 | *Solanum tuberosum* |
| S.tuberosum_S-RNase_12 | MZ561415.1 | *Solanum tuberosum* |
| S.tuberosum_S-RNase_13 | MZ561416.1 | *Solanum tuberosum* |
| S.tuberosum_S-RNase_14 | MZ561417.1 | *Solanum tuberosum* |
| Prunus_dulcis_S-RNase | EF619314.1 | *Prunus dulcis* |
| S.tuberosum_DM_1-3_516_S-RNase | NA | *Solanum tuberosum* Group phureja |
| S.tuberosum_RH89-039-16_S-RNase | NA | *Solanum tuberosum* |
| S.chacoense_M6_S-RNase | NA | *Solanum chacoense* |
| S.lycopersucum_SLF1 | KJ814895.1 | *Solanum lycopersicum* |
| S.pimpinellifolium_SLF2 | KJ814870.1 | *Solanum pimpinellifolium* |
| P.hybrida_SLF3 | AB933074.1 | *Petunia x hybrida* |
| S.lycopersucum_SLF4 | KJ814943.1 | *Solanum lycopersicum* |
| S.lycopersucum_SLF5 | NM_001302892.1 | *Solanum lycopersicum* |
| S.pimpinellifolium_SLF6 | KJ814873.1 | *Solanum pimpinellifolium* |
| S.lycopersicoides_SLF7 | KU987625.1 | *Solanum lycopersicoides* |
| P.hybrida_SLF8 | AB933130.1 | *Petunia x hybrida* |
| S.lycopersucum_SLF9 | KJ814898.1 | *Solanum lycopersicum* |
| S.pimpinellifolium_SLF10 | KJ814876.1 | *Solanum pimpinellifolium* |
| S.lycopersucum_SLF11 | NM_001301437.1 | *Solanum lycopersicum* |
| S.lycopersucum_SLF12 | NM_001301441.1 | *Solanum lycopersicum* |
| S.lycopersucum_SLF13 | NM_001301435.1 | *Solanum lycopersicum* |
| S.lycopersucum_SLF14 | KJ814903.1 | *Solanum lycopersicum* |
| S.lycopersucum_SLF15 | AB933105.1 | *Solanum lycopersicum* |
| P.hybrida_SLF16 | AB933086.1 | *Petunia x hybrida* |
| S.pimpinellifolium_SLF17 | KU987617.1 | *Solanum pimpinellifolium* |
| P.hybrida_SLF18 | AB933139.1 | *Petunia x hybrida* |
| Prunus_dulcis_SLF19 | MH316065.1 | *Prunus dulcis* |
| S.habrochaites_SLF20 | KU960917.1 | *Solanum habrochaites* |
| S.habrochaites_SLF21 | KU960918.1 | *Solanum habrochaites* |
| S.lycopersicoides_SLF22 | KU960924.1 | *Solanum lycopersicoides* |
| S.habrochaites_SLF23 | KU960920.1 | *Solanum habrochaites* |
| Prunus_dulcis_SLF24 | MH316068.1 | *Prunus dulcis* |

Table S4. *S-RNase* and *SLF* sequences used as comparison for the construction of phylogenies (Supplemental Figures 2 and 3).

|  | MSII1813-2 | | | MSJJ1821F2-041 | | | MSJJ1821F2-049 | | | MSJJ1821F2-091 | | |
| --- | --- | --- | --- | --- | --- | --- | --- | --- | --- | --- | --- | --- |
|  | Rep 1 | Rep 2 | Rep 3 | Rep 1 | Rep 2 | Rep 3 | Rep 1 | Rep 2 | Rep 3 | Rep 1 | Rep 2 | Rep 3 |
| Solver.v1.03_VERG035240.1 | 15.87818 | 18.61916 | 19.87628 | 11.10638 | 12.31979 | 9.535234 | 22.48761 | 16.86629 | 17.7268 | 15.61083 | 16.26674 | 21.93393 |

Table S5. Transcripts per million (TPM) values for pollen expressed Solver.v1.03_VERG035240.1, a phospholipid:diacylglycerol acyltransferase (PDAT) in the chromosome 3 QTL. Male sterile MSJJ1821F2-041 had significantly reduced expression relative to the other male fertile F2 progeny and MSII1813-2.

Solver.v1.03_VERG035240.1 MSLLRRRRTPENEIQSDVEPKLDAEEDDKKSKKKNVKSGKKKKWSCIDNCCWFVGCICCV 60

Soltu.DM.03G036770.1 MSLLRRRKAPENEIQSNVEPKLDAEEDDKKSKKKNVKSGKKKKWSCIDNCCWFVGCICCV 60

Soltu.DM1S1.03G035420.1 MSLLRRRKAAENEIQSYVEPKLDAEEDDKKSKKKNVKSGKKKKWSCIDNCCWFVGCICCV 60

*******:: ****** *******************************************

Solver.v1.03_VERG035240.1 WWILLFLYNAMPASFPQYVTEAITGPLPDPPGIKLQKEGLKAKHPVVFIPGIVTCGLELW 120

Soltu.DM.03G036770.1 WWILLFLYNAMPASFPQYVAEAITGPLPDPPGIKLQKEGLKAKHPVVFIPGIVTCGLELW 120

Soltu.DM1S1.03G035420.1 WWILLFLYNAMPASFPQYVTEAITGPLPDPPGIKLQKEGLKAKHPVVFIPGIVTCGLELW 120

*******************:****************************************

Solver.v1.03_VERG035240.1 EGHQCAEGLFRKRLWGGTFGEVYKRPLCWVNHMTLDNETGMDPPGIRVRPVSGLVAADYF 180

Soltu.DM.03G036770.1 EGHQCAEGLFRKRLWGGTFGEVYKRPLCWVNHMTLDNETGMDPPGIRVRPVSGLVAADYF 180

Soltu.DM1S1.03G035420.1 EGHQCAEGLFRKRLWGGTFGEVYKRPLCWVNHMTLDNETGMDPPGIRVRPVSGLVAADYF 180

************************************************************

Solver.v1.03_VERG035240.1 APGYFVWAVLIANLARIGYEEKTMYMAAYDWRLAFQNTEVRDQTLSRIKSNIELMVATSG 240

Soltu.DM.03G036770.1 APGYFVWAVLIANLARIGYEEKTMYMAAYDWRLAFQNTEVRDQTLSRIKSNIELMVATSG 240

Soltu.DM1S1.03G035420.1 APGYFVWAVLIANLARIGYEEKTMYMAAYDWRLAFQNTEVRDQTLSRIKSNIELMVATSG 240

************************************************************

Solver.v1.03_VERG035240.1 KKAVIVPHSMGVVYFLHFMKWVEAPAPVGGGGGPDWCAKHIKAVMNIGGPLLGVPKSISG 300

Soltu.DM.03G036770.1 KKAVIVPHSMGVVYFLHFMKWVEAPAPVGGGGGPDWCAKHIKAVMNIGGPLLGVPKSIAG 300

Soltu.DM1S1.03G035420.1 KKAVIVPHSMGVVYFLHFMKWVEAPAPVGGGGGPDWCAKNIKAVMNIGGPLLGVPKAIAG 300

***************************************:****************:*:*

Solver.v1.03_VERG035240.1 LFSAEARDIAVARALAPGVLDTDLFHFQTLEHIMKMSRTWDATMSMIPRGGDTIWGGLDW 360

Soltu.DM.03G036770.1 LFSAEARDIAVARALAPGVLDTDIFHFQTLEHIMKMSRTWDATMSMIPRGGDTIWGGLDW 360

Soltu.DM1S1.03G035420.1 LFSAEARDIAVARALAPGVLDTDIFHFQTLEHIMKMSRTWDATMSMIPRGGDTIWGGLDW 360

***********************:************************************

Solver.v1.03_VERG035240.1 SPEEGYSPCKSKSRDDAAQNSGHHENQTTDSKAKYYSYGRMMSFGKDAAEAHPSDLKRID 420

Soltu.DM.03G036770.1 SPEEGYSPCKSKSRDDAAQNSGHHENQTTDSKAKYYSYGRMMSFGKDATEAHPSDLKRID 420

Soltu.DM1S1.03G035420.1 SPEEGYSPCKSKSRDDAAQNSGHHENQTTDSKAKYYSYGRMMSFGKDAAEAHPSDLKRID 420

************************************************:***********

Solver.v1.03_VERG035240.1 FRDAVKGSNVANNTCDVWNEYQDMGVSGTKAVEEYKVYTAGEIVDLLNFVAPKMMARGNA 480

Soltu.DM.03G036770.1 FRDAVKGSNVANNTCDVWNEYQDMGVSGTKAVEEYKVYTAGEIVDLLNFVAPKMMARGNA 480

Soltu.DM1S1.03G035420.1 FRDAVKGTNVANNTCDVWNEYQDMGVSGTKAVEEYKVYTAGEIVDLLNFVAPKMMARGNA 480

*******:****************************************************

Solver.v1.03_VERG035240.1 HFSYGIADDLDDPKYSHYKYWSNPLETKLPNAPDMEIYSMYGVGIETERAYVYKRIPTAG 540

Soltu.DM.03G036770.1 HFSYGIADDLDDPKYSHYKYWSNPLETKLPNAPDMEIYSMYGVGIETERAYVYKRIPTAG 540

Soltu.DM1S1.03G035420.1 HFSYGIADDLDDPKYSHYKYWSNPLETKLPNAPDMEIYSMYGVGIETERAYVYKRIPTAG 540

************************************************************

Solver.v1.03_VERG035240.1 CNIPFQIDTSADDGDEGSCLKSGVFTIDGDETVPALSAGFMCAKGWRGRTRFNPSGIKTY 600

Soltu.DM.03G036770.1 CNIPFQIDTSADDGDEGSCLKSGVYTIDGDETVPALSAGFMCAKGWRGRTRFNPSGIKTY 600

Soltu.DM1S1.03G035420.1 CNIPFQIDTSADDGDEGSCLKSGVYTIDGDETVPALSAGFMCAKGWRGRTRFNPSGIKTY 600

************************:***********************************

Solver.v1.03_VERG035240.1 IREYFHAPPANLLEGRGTQSGAHVDIMGNFALIEDIMRVAAGGTSENLGESIVVSDQSPF 660

Soltu.DM.03G036770.1 IREYFHSPPANLLEGRGTQSGAHVDIMGNFALIEDIMRVAAGGTSENLGGDQVYSDIFKW 660

Soltu.DM1S1.03G035420.1 IREYFHAPPANLLEGRGTQSGAHVDIMGNFALIEDIMRVAAGGTSENLGGDQVYSDIFKW 660

******:****************************************** . * ** :

Solver.v1.03_VERG035240.1 LDRYLQSLSLYLSIYSV 677

Soltu.DM.03G036770.1 SEKINLRL--------- 668

Soltu.DM1S1.03G035420.1 SEKINLRL--------- 668

:: *

Figure S5. Multiple sequence alignment of Soltu.DM.03G036770.1 and the DM1S1 and MSII1813-2 orthologs Soltu.DM1S1.03G035420.1 and Solver.v1.03_VERG035240.1 respectively. Generated using CLUSTAL O(1.2.4) multiple sequence alignment.


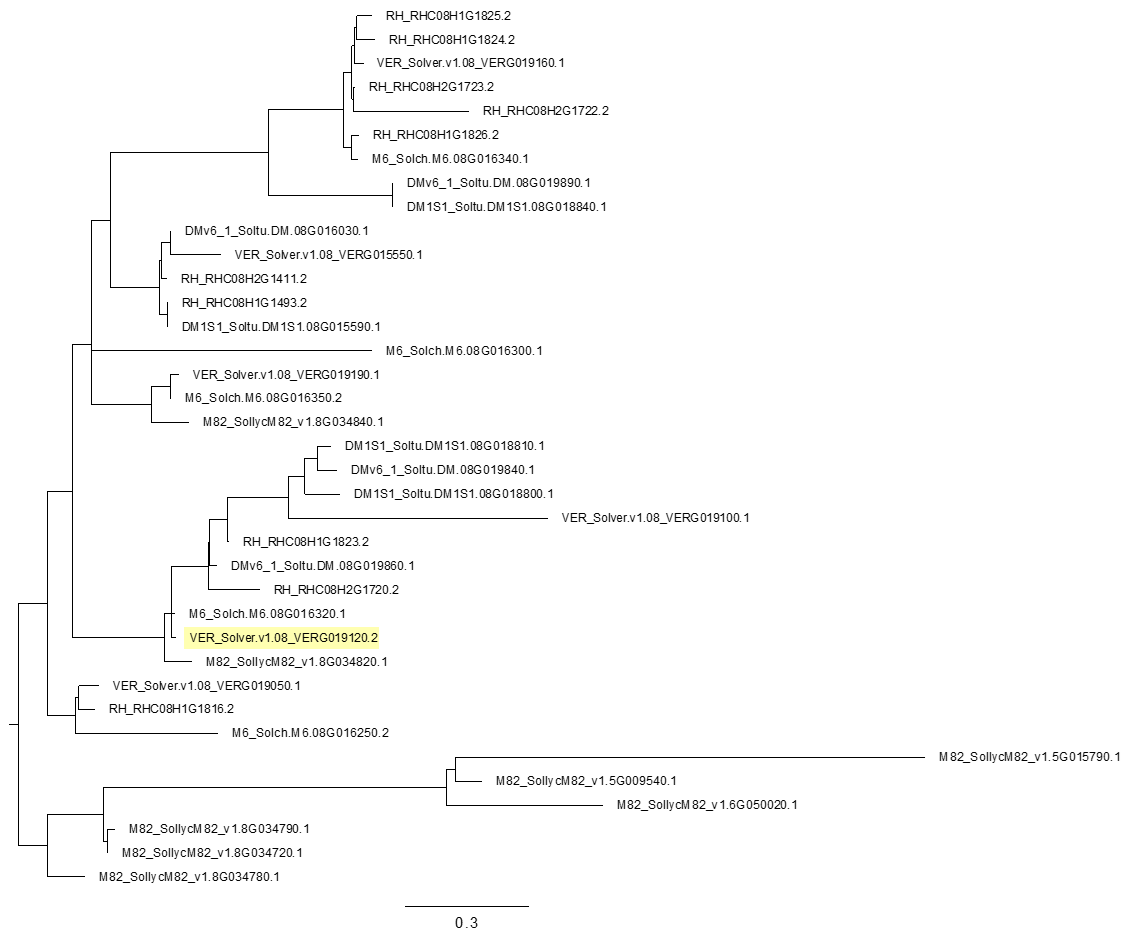


Figure S6. Gene tree of pectinacetylesterases from *S. verrucosum* MSII1813-2, *S. chacoense* M6, *S. tuberosum* DM, *S. tuberosum* DM1S1, *S. tuberosum* RH, and *Solanum lycopersicum* M82 that was used as an outgroup. Of the *S. verrucosum* pectinacetylesterases, Solver.v1.08_VERG019120 (highlighted in yellow) has elevated expression in pollen.

Solver.v1.09_VERG000590.1 MFFKIVSLIVSSLFVVTNSENFGFIYNGFNRANLSLDGIAQLTSNGLLELTNTSRLQKGH 60

Soltu.DM.09G000840.1 MFFKIVSLIVSALFVVTNSEDFGFIYNGFNRANLSLDGIAQFTSNGLLELTNTSRLQKGH 60

Soltu.DM1S1.09G000610.1 MFFKIVSLIVSALFVVTNSEDFGFIYNGFNRANLSLDGIAQFTSNGLLELTNTSRLQKGH 60

***********:********:********************:******************

Solver.v1.09_VERG000590.1 AFYSTPINFKNLQNGSNFSFSTTFVFAIVPSVLPGHGMAFVIAPVGGLVDALPSPFLGLF 120

Soltu.DM.09G000840.1 AFYPTPINFKNLQNGSNFSFPTTFVFAIVPSVLPGHGMAFVIAPVGGLVDALPSPFLGLF 120

Soltu.DM1S1.09G000610.1 AFYPTPINFKNLQNGSNFSFSTTFVFAIVPSVLPGHGMAFVIAPVGGLVDALPSPFLGLF 120

*** **************** ***************************************

Solver.v1.09_VERG000590.1 NDNTTGRVSNHVFAVEFDTLQNREFNDIDGNHVGIDINGLKSVESKTAGYYYGVKFNNMT 180

Soltu.DM.09G000840.1 NDNTTGRVSNHVFAVEFDTLQNREFNDIDGNHVGIDINGLKSVESKTAGYYYGVKFNNMT 180

Soltu.DM1S1.09G000610.1 NDNTTGRVSNHVFAVEFDTLQNREFNDIDGNHVGIDINGLKSIESKTAGYYHGVKFDNMT 180

******************************************:********:****:***

Solver.v1.09_VERG000590.1 LASGQPMQGWVDYNGMAKQINVTLAPMNVAKPNAPLLSLLYDLSPILNETMYIGFSGSTG 240

Soltu.DM.09G000840.1 LASGQPMQGWVDYDGVAKQINVTLAPMNVAKPNVPLLSLLYDLSPILNETMHIGFSGSTG 240

Soltu.DM1S1.09G000610.1 LASGQPMQGWVDYDGVAKQINVTLAPMNVAKPNAPLLSLLYDLSPILNETMYIGFSGSTG 240

*************:*:*****************.*****************:********

Solver.v1.09_VERG000590.1 SVVSTQYVLGWSFEMNGIAQGLDLARLPKLPRVGPKKQSKLLLIALPMISAVVVVIAFSV 300

Soltu.DM.09G000840.1 SVVSTQYVLGWSFEMNGIAQGLDLARLPKLPRVGPKKQSKLLLVALPMISAVVVVIAFSV 300

Soltu.DM1S1.09G000610.1 SVVSTQYVLGWSFEMNGIAQGLDLARLPKLPRVGPKKQSKLLLIALPMISAVVVVIAFSV 300

*******************************************:****************

Solver.v1.09_VERG000590.1 LIYYVGRKRRFAELLEDWELEYGPHRFKYKDLYIATKGFANKELLGCGGFGRVYKGVLPT 360

Soltu.DM.09G000840.1 LIYYVGRKRKFAELLEDWELEYGPHRFKYKDLYIATKGFANKKLLGCGGFGRVYKGVLPT 360

Soltu.DM1S1.09G000610.1 LIYYVGRKRKFAELLEDWELEYGPHRFKYKDLYIATKGFANKELLGCGGFGRVYKGVLPT 360

*********:********************************:*****************

Solver.v1.09_VERG000590.1 SSIEVAVKKVSHDSKQGLREFVAEIVSIGCLRHRNLVPLLGYCRRKGELLLVYEYMPNGS 420

Soltu.DM.09G000840.1 SSIEVAVKKVSHDSKQGLREFVAEIVSIGRLRHRNLVQLLGYCRRKGELLLVYEYMPNGS 420

Soltu.DM1S1.09G000610.1 SSIEVAVKKVSHDSKQGLREFVAEIVSIGRLRHRNLVPLLGYCRRKGELLLVYEYMPNGS 420

***************************** ******* **********************

Solver.v1.09_VERG000590.1 LDKFLYDKPICALSWNQRFRVIKGVASALVYLHEEWEQVVIHRDVKASNVLLDSELNAKL 480

Soltu.DM.09G000840.1 LDKFLYDKPICALSWNQRFRVIKGVASALVYLHEEWEQVVIHRDVKASNVLLDSELNAKL 480

Soltu.DM1S1.09G000610.1 LDKFLYDKPICALSWNQRFRVIKGVASALVYLHEEWEQVVIHRDVKASNVLLDSELNAKL 480

************************************************************

Solver.v1.09_VERG000590.1 GDFGLARLYDHGSDPLTTHVVGTVGYLAPEQTRTGKATTISDVYAFGAFLLEVACGRRPI 540

Soltu.DM.09G000840.1 GDFGLARLYDHGTDPLTTHVVGTVGYLAPEQTRTGKATTISDVYAFGAFLLEVACGRRPI 540

Soltu.DM1S1.09G000610.1 GDFGLARLYDHGTDPLTTHVVGTVGYLAPEQTRTGKATTISDVYAFGAFLLEVACGRRPI 540

************:***********************************************

Solver.v1.09_VERG000590.1 DPRVSDEDIVLVDYVFSCWSR---------------------------------EPTTRP 567

Soltu.DM.09G000840.1 DPRVSDEDIVLVDYVFSCWSRGDILQSIDQNLGNEYVKEEVELVLKLGLVCSQTEPTRRR 600

Soltu.DM1S1.09G000610.1 DPRVSDEDIVLVDYVFSCWSRGDILQSIDQNLGNEYVKEEVELVLKLGLVCSQTEPTTRP 600

********************* *** *

Solver.v1.09_VERG000590.1 SMRQVLIYLEDIVPPPLPELSLLQSSAHYFSFPGFDHHLSMSSSFSPEKVLSYPSTDSNS 627

Soltu.DM.09G000840.1 SMRQVLIYLEDIVPPPLPELSLLQSSAHYFSFPGFDHHLSMSSSFSPEKVLSYPSTDSNS 660

Soltu.DM1S1.09G000610.1 SMRQVLIYLEDIVPPPLPELSLLQSSAHDFSFPGFDHHLSMSSSFSPYKVLSYPSTDSDS 660

**************************** ****************** **********:*

Solver.v1.09_VERG000590.1 LPFTGP* 633

Soltu.DM.09G000840.1 LPFTGP- 666

Soltu.DM1S1.09G000610.1 LPFTGP- 666

******

Figure S7. Multiple sequence alignment of Soltu.DM.09G000840.1 and the DM1S1 and MSII1813-2 orthologs Soltu.DM1S1.09G000610.1 and Solver.v1.09_VERG000590.1, respectively. A deletion is evident in the *S. verrucosum* ortholog Solver.v1.09_VERG000590.1 relative to the DM and DM1S1. Generated using CLUSTAL O(1.2.4) multiple sequence alignment.

| Pseudomolecule | Total Length | Total Sequence Length | Total Gap Length | Number of Gaps |
| --- | --- | --- | --- | --- |
| chr01_VER | 90,421,390 | 90,420,178 | 1,212 | 12 |
| chr02_VER | 46,831,522 | 46,831,522 | 0 | 0 |
| chr03_VER | 59,844,308 | 59,843,904 | 404 | 4 |
| chr04_VER | 68,653,275 | 68,652,770 | 505 | 5 |
| chr05_VER | 50,246,749 | 50,246,143 | 606 | 6 |
| chr06_VER | 54,216,895 | 54,216,794 | 101 | 1 |
| chr07_VER | 54,327,197 | 54,326,692 | 505 | 5 |
| chr08_VER | 56,348,775 | 56,348,573 | 202 | 2 |
| chr09_VER | 60,250,147 | 60,249,743 | 404 | 4 |
| chr10_VER | 57,124,562 | 57,124,461 | 101 | 1 |
| chr11_VER | 43,480,931 | 43,480,931 | 0 | 0 |
| chr12_VER | 58,927,637 | 58,927,031 | 606 | 6 |
| Total Pseudomolecules | 700,673,388 | 700,668,742 | 4,646 | 46 |
|  |  |  |  |  |
| Unanchored Scaffolds | 37,903,700 | 37,903,700 | 0 | 0 |
| Total Assembly | 738,577,088 | 738,572,442 | 4,646 | 46 |

Table S6. Genome assembly metrics for *S. verrucosum* MSII1813-2.

| Class of BUSCO | Genome  Assembly | Annotation  Working Models |
| --- | --- | --- |
|  |  |  |
| Complete BUSCOs | 1,603 (99.3%) | 1,509 (93.5%) |
| Complete and single-copy BUSCOs (S) | 1,570 (97.3%) | 1,341 (83.1%) |
| Complete and duplicated BUSCOs (D) | 33 (2.0%) | 168 (10.4%) |
| Fragmented BUSCOs (F) | 5 (0.3%) | 79 (4.9%) |
| Missing BUSCOs (M) | 6 (0.4%) | 26 (1.6%) |
| Total BUSCOs searched | 1614 | 1614 |
| BUSCO v5.4.3 with embryophyta_odb10 |  |  |

Table S7. Benchmarking universal single copy orthologs in the *S. verrucosum* MSII1813-2 genome sequence and annotation.

|  | Number of Elements | Length Occupied | Percentage of Sequence |
| --- | --- | --- | --- |
| Retroelements | 219,804 | 256,052,768 | 34.67% |
| DNA transposons | 74,800 | 26,044,287 | 3.53% |
| Rolling Circles | 6,432 | 1,516,577 | 0.21% |
| Unclassified interspersed repeats | 623,575 | 183,628,938 | 24.86% |
| Small RNA | 15,118 | 22,617,849 | 3.06% |
| Satellites | 14,324 | 4,230,407 | 0.57% |
| Simple repeats | 3,034 | 480,129 | 0.07% |
| Low complexity | 288 | 78,875 | 0.01% |

Table S8. Repetitive sequences identified in *S. verrucosum* MSII1813-2.

|  | Working Models |
| --- | --- |
| Number of Gene Models | 48,510 |
| Maximum Transcript Length (bp) | 31,467 |
| Maximum CDS Length (bp) | 31,467 |
|  |  |
| Average Transcript Length (bp) | 1,073.1 |
| Average CDS Length (bp) | 1,073.1 |
| Average Exon Length (bp) | 230.6 |
| Average Intron Length (bp) | 610.5 |
|  |  |
| Single Exon Transcripts | 20,406 |

Table S9. Protein coding genes annotated in *S. verrucosum* MSII1813-2.

| **DM_v6.1 gene** | **VER_v1 gene** | **DM1S1_v1 gene** | **Functional Annotation** | **Comment** |
| --- | --- | --- | --- | --- |
| Soltu.DM.11G020470.1 | Solver.v1.11_VERG018950 | Soltu.DM1S1.11G020610 | Pathogenesis-related thaumatin superfamily protein |  |
| Soltu.DM.11G020480.1 | Solver.v1.11_VERG018960 | Soltu.DM1S1.11G020620 | Fusaric acid resistance protein-like domain containing protein |  |
| Soltu.DM.11G020490.1 | Solver.v1.11_VERG018970 | Soltu.DM1S1.11G020630 | UDP-XYL synthase |  |
| Soltu.DM.11G020500.1 |  | Soltu.DM1S1.11G020640 | Histone superfamily protein |  |
| Soltu.DM.11G020520.1 | Solver.v1.11_VERG019020 | Soltu.DM1S1.11G020670 | Histone superfamily protein |  |
| Soltu.DM.11G020580.1 |  | Soltu.DM1S1.11G020750 | response regulator |  |
| Soltu.DM.11G020600.1 | Solver.v1.11_VERG019100 | Soltu.DM1S1.11G020770 | conserved hypothetical protein |  |
| Soltu.DM.11G020610.1 | Solver.v1.11_VERG019110 | Soltu.DM1S1.11G020780 | serine carboxypeptidase-like |  |
| Soltu.DM.11G020620.2 |  | Soltu.DM1S1.11G020790 | hypothetical protein |  |
| Soltu.DM.11G020630.1 | Solver.v1.11_VERG019120 |  | SNF7 family protein |  |
| Soltu.DM.11G020640.1 |  | Soltu.DM1S1.11G020810 | xyloglucan endotransglucosylase/hydrolase |  |
| Soltu.DM.11G020650.1 | Solver.v1.11_VERG019150 | Soltu.DM1S1.11G020820 | Ribosomal RNA processing Brix domain protein |  |
| Soltu.DM.11G020660.1 | Solver.v1.11_VERG019160 | Soltu.DM1S1.11G020830 | purple acid phosphatase |  |
| Soltu.DM.11G020670.1 | Solver.v1.11_VERG019170 | Soltu.DM1S1.11G020840 | purple acid phosphatase |  |
| Soltu.DM.11G020680.1 | Solver.v1.11_VERG019200 | Soltu.DM1S1.11G020850 | RNA-binding CRS1 / YhbY (CRM) domain-containing protein |  |
| Soltu.DM.11G020690.2 | Solver.v1.11_VERG019210 |  | conserved hypothetical protein |  |
| Soltu.DM.11G020700.2 | Solver.v1.11_VERG019220 | Soltu.DM1S1.11G020870 | signal recognition particle binding |  |
| Soltu.DM.11G020710.2 | Solver.v1.11_VERG019230 | Soltu.DM1S1.11G020880 | Exostosin family protein |  |
| Soltu.DM.11G020720.2 | Solver.v1.11_VERG019240 | Soltu.DM1S1.11G020890 | Major facilitator superfamily protein |  |
| Soltu.DM.11G020730.1 | Solver.v1.11_VERG019250 | Soltu.DM1S1.11G020900 | conserved hypothetical protein |  |
| Soltu.DM.11G020740.2 | Solver.v1.11_VERG019290 | Soltu.DM1S1.11G020910 | Shugoshin C terminus |  |
| Soltu.DM.11G020750.1 | Solver.v1.11_VERG019300 | Soltu.DM1S1.11G020920 | Protein of unknown function (DUF1635) |  |
| Soltu.DM.11G020760.1 | Solver.v1.11_VERG019310 | Soltu.DM1S1.11G020930 | DNA ligase |  |
| Soltu.DM.11G020820.2 | Solver.v1.11_VERG019370 | Soltu.DM1S1.11G021000 | Domain of unknown function (DUF966) |  |
| Soltu.DM.11G020830.1 | Solver.v1.11_VERG019380 | Soltu.DM1S1.11G021010 | copper/zinc superoxide dismutase |  |
| Soltu.DM.11G020840.2 | Solver.v1.11_VERG019390 | Soltu.DM1S1.11G021020 | C2H2-type zinc finger family protein |  |
| Soltu.DM.11G020850.3 | Solver.v1.11_VERG019400 | Soltu.DM1S1.11G021030 | ribosomal protein L9 |  |
| Soltu.DM.11G020870.1 | Solver.v1.11_VERG019420 | Soltu.DM1S1.11G021050 | Histone superfamily protein |  |
| Soltu.DM.11G020880.1 | Solver.v1.11_VERG019430 | Soltu.DM1S1.11G021060 | Pheophorbide a oxygenase family protein with Rieske [2Fe-2S] domain |  |
| Soltu.DM.11G020890.1 | Solver.v1.11_VERG019440 | Soltu.DM1S1.11G021070 | conserved hypothetical protein |  |
| Soltu.DM.11G020900.1 | Solver.v1.11_VERG019450 | Soltu.DM1S1.11G021080 | hypothetical protein |  |
| Soltu.DM.11G020910.1 | Solver.v1.11_VERG019460 | Soltu.DM1S1.11G021090 | cyclin-dependent kinase-subunit |  |
| Soltu.DM.11G020920.1 | Solver.v1.11_VERG019470 | Soltu.DM1S1.11G021100 | Acyl-CoA N-acyltransferase with RING/FYVE/PHD-type zinc finger domain |  |
| Soltu.DM.11G020940.1 | Solver.v1.11_VERG019480 | Soltu.DM1S1.11G021110 | Ring/U-Box superfamily protein |  |
| Soltu.DM.11G020950.1 | Solver.v1.11_VERG019490 | Soltu.DM1S1.11G021120 | RING/U-box superfamily protein |  |
| Soltu.DM.11G020960.1 | Solver.v1.11_VERG019500 | Soltu.DM1S1.11G021130 | serine carboxypeptidase-like |  |
| Soltu.DM.11G020970.1 | Solver.v1.11_VERG019510 | Soltu.DM1S1.11G021140 | Vacuolar-sorting protein 54, of GARP complex domain containing protein |  |
| Soltu.DM.11G020980.1 | Solver.v1.11_VERG019540 | Soltu.DM1S1.11G021150 | vacuolar protein sorting |  |
| Soltu.DM.11G020990.1 | Solver.v1.11_VERG019550 | Soltu.DM1S1.11G021160 | Cytochrome P450 superfamily protein |  |
| Soltu.DM.11G021000.1 |  | Soltu.DM1S1.11G021170 | Serine carboxypeptidase S28 family protein |  |
| Soltu.DM.11G021010.1 | Solver.v1.11_VERG019570 | Soltu.DM1S1.11G021180 | alpha/beta-Hydrolases superfamily protein |  |
| Soltu.DM.11G021020.1 | Solver.v1.11_VERG019590 | Soltu.DM1S1.11G021190 | ATP10 protein domain containing protein |  |
| Soltu.DM.11G021030.4 | Solver.v1.11_VERG019610 | Soltu.DM1S1.11G021200 | Eukaryotic aspartyl protease family protein |  |
| Soltu.DM.11G021040.1 | Solver.v1.11_VERG019620 | Soltu.DM1S1.11G021210 | RNA-binding (RRM/RBD/RNP motifs) family protein |  |
| Soltu.DM.11G021050.1 | Solver.v1.11_VERG019630 | Soltu.DM1S1.11G021220 | hydroxycinnamoyl-CoA shikimate/quinate hydroxycinnamoyl transferase |  |
| Soltu.DM.11G021060.3 | Solver.v1.11_VERG019640 | Soltu.DM1S1.11G021230 | Protein kinase superfamily protein |  |
| Soltu.DM.11G021070.1 | Solver.v1.11_VERG019650 | Soltu.DM1S1.11G021240 | magnesium (Mg) transporter |  |
| Soltu.DM.11G021080.1 | Solver.v1.11_VERG019670 | Soltu.DM1S1.11G021270 | UDP-Glycosyltransferase superfamily protein |  |
| Soltu.DM.11G021090.1 | Solver.v1.11_VERG019660 | Soltu.DM1S1.11G021260 | UDP-glucosyl transferase 72E1 |  |
| Soltu.DM.11G021110.1 | Solver.v1.11_VERG019680 | Soltu.DM1S1.11G021280 | RecQ-mediated genome instability protein 2 domain containing protein |  |
| Soltu.DM.11G021120.1 | Solver.v1.11_VERG019690 | Soltu.DM1S1.11G021290 | GDSL-like Lipase/Acylhydrolase superfamily protein |  |
| Soltu.DM.11G021130.1 | Solver.v1.11_VERG019700 | Soltu.DM1S1.11G021300 | Lecithin:cholesterol acyltransferase family protein |  |
| Soltu.DM.11G021140.1 | Solver.v1.11_VERG019710 | Soltu.DM1S1.11G021310 | UDP-D-apiose/UDP-D-xylose synthase |  |
| Soltu.DM.11G021150.1 | Solver.v1.11_VERG019720 | Soltu.DM1S1.11G021320 | Phototropic-responsive NPH3 family protein |  |
| Soltu.DM.11G021160.1 | Solver.v1.11_VERG019730 | Soltu.DM1S1.11G021330 | reticulata-related |  |
| Soltu.DM.11G021170.1 | Solver.v1.11_VERG019740 | Soltu.DM1S1.11G021340 | alpha-adaptin |  |
| Soltu.DM.11G021180.1 | Solver.v1.11_VERG019750 | Soltu.DM1S1.11G021350 | PHD finger family protein |  |
| Soltu.DM.11G021190.1 | Solver.v1.11_VERG019760 | Soltu.DM1S1.11G021360 | DNA/RNA helicase protein |  |
| Soltu.DM.11G021200.1 | Solver.v1.11_VERG019770 | Soltu.DM1S1.11G021370 | Transmembrane amino acid transporter family protein |  |
| Soltu.DM.11G021210.1 | Solver.v1.11_VERG019780 | Soltu.DM1S1.11G021380 | cellulose synthase-like A02 |  |
| Soltu.DM.11G021220.1 | Solver.v1.11_VERG019790 | Soltu.DM1S1.11G021390 | U2 snRNP auxiliary factor small subunit, putative |  |
| Soltu.DM.11G021230.1 | Solver.v1.11_VERG019800 | Soltu.DM1S1.11G021400 | histone deacetylase 2C |  |
| Soltu.DM.11G021240.4 | Solver.v1.11_VERG019810 | Soltu.DM1S1.11G021410 | endonucleases |  |
| Soltu.DM.11G021250.1 |  | Soltu.DM1S1.11G021420 | conserved hypothetical protein |  |
| Soltu.DM.11G021260.1 | Solver.v1.11_VERG019840 | Soltu.DM1S1.11G021430 | MORN (Membrane Occupation and Recognition Nexus) repeat-containing protein |  |
| Soltu.DM.11G021280.1 | Solver.v1.11_VERG019850 | Soltu.DM1S1.11G021440 | arogenate dehydratase |  |
| Soltu.DM.11G021290.3 | Solver.v1.11_VERG019860 | Soltu.DM1S1.11G021450 | nucleobase-ascorbate transporter |  |
| Soltu.DM.11G021300.2 | Solver.v1.11_VERG019870 | Soltu.DM1S1.11G021460 | Syntaxin/t-SNARE family protein |  |
| Soltu.DM.11G021310.1 | Solver.v1.11_VERG019880 | Soltu.DM1S1.11G021470 | Tetratricopeptide repeat (TPR)-like superfamily protein |  |
| Soltu.DM.11G021330.1 | Solver.v1.11_VERG019890 | Soltu.DM1S1.11G021480 | conserved hypothetical protein |  |
| Soltu.DM.11G021340.1 | Solver.v1.11_VERG019900 | Soltu.DM1S1.11G021490 | Stress responsive A/B Barrel Domain |  |
| Soltu.DM.11G021350.1 | Solver.v1.11_VERG019910 | Soltu.DM1S1.11G021520 | Stress responsive A/B Barrel Domain |  |
| Soltu.DM.11G021360.2 | Solver.v1.11_VERG019920 | Soltu.DM1S1.11G021530 | tRNAisopentenyltransferase |  |
| Soltu.DM.11G021370.1 | Solver.v1.11_VERG019930 | Soltu.DM1S1.11G021540 | Family of unknown function (DUF662) |  |
| Soltu.DM.11G021380.1 | Solver.v1.11_VERG019940 | Soltu.DM1S1.11G021550 | XS domain containing protein |  |
| Soltu.DM.11G021390.1 | Solver.v1.11_VERG019950 | Soltu.DM1S1.11G021560 | pleiotropic drug resistance |  |
| Soltu.DM.11G021400.1 | Solver.v1.11_VERG019960 | Soltu.DM1S1.11G021570 | G10 family protein |  |
| Soltu.DM.11G021410.1 | Solver.v1.11_VERG019970 |  | hypothetical protein |  |
| Soltu.DM.11G021420.1 | Solver.v1.11_VERG019980 | Soltu.DM1S1.11G021590 | histone deacetylase |  |
| Soltu.DM.11G021440.1 | Solver.v1.11_VERG019990 | Soltu.DM1S1.11G021600 | Adenine nucleotide alpha hydrolases-like superfamily protein |  |
| Soltu.DM.11G021450.11 | Solver.v1.11_VERG020000 | Soltu.DM1S1.11G021610 | alkaline/neutral invertase |  |
| Soltu.DM.11G021460.1 | Solver.v1.11_VERG020010 | Soltu.DM1S1.11G021620 | protein tyrosine phosphatases;protein tyrosine phosphatases |  |
| Soltu.DM.11G021470.1 | Solver.v1.11_VERG020020 | Soltu.DM1S1.11G021630 | Protein kinase superfamily protein |  |
| Soltu.DM.11G021480.1 | Solver.v1.11_VERG020040 | Soltu.DM1S1.11G021640 | Protein kinase superfamily protein |  |
| Soltu.DM.11G021490.1 | Solver.v1.11_VERG020050 | Soltu.DM1S1.11G021650 | cyclophilin71 |  |
| Soltu.DM.11G021500.1 | Solver.v1.11_VERG020060 | Soltu.DM1S1.11G021660 | 60S acidic ribosomal protein family |  |
| Soltu.DM.11G021510.1 | Solver.v1.11_VERG020070 | Soltu.DM1S1.11G021670 | DNA polymerase epsilon catalytic subunit |  |
| Soltu.DM.11G021520.1 | Solver.v1.11_VERG020080 | Soltu.DM1S1.11G021680 | conserved hypothetical protein |  |
| Soltu.DM.11G021530.4 | Solver.v1.11_VERG020090 | Soltu.DM1S1.11G021690 | NAD(P)-linked oxidoreductase superfamily protein |  |
| Soltu.DM.11G021540.1 | Solver.v1.11_VERG020100 | Soltu.DM1S1.11G021700 | hypothetical protein |  |
| Soltu.DM.11G021550.1 | Solver.v1.11_VERG020110 | Soltu.DM1S1.11G021720 | AGAMOUS-like |  |
| Soltu.DM.11G021580.1 | Solver.v1.11_VERG020120 | Soltu.DM1S1.11G021740 | fatty acid reductase |  |
| Soltu.DM.11G021590.2 | Solver.v1.11_VERG020160 | Soltu.DM1S1.11G021760 | fatty acid reductase |  |
| Soltu.DM.11G021610.1 | Solver.v1.11_VERG020170 | Soltu.DM1S1.11G021810 | Jojoba acyl CoA reductase-related male sterility protein | Peak SNP region |
| Soltu.DM.11G021620.1 | Solver.v1.11_VERG020180 | Soltu.DM1S1.11G021820 | homolog of histone chaperone HIRA | Peak SNP region |
| Soltu.DM.11G021630.1 | Solver.v1.11_VERG020190 | Soltu.DM1S1.11G021830 | Tetratricopeptide repeat (TPR)-like superfamily protein | Peak SNP region |
| Soltu.DM.11G021640.1 | Solver.v1.11_VERG020200 | Soltu.DM1S1.11G021840 | AAA-type ATPase family protein | Peak SNP region |
| Soltu.DM.11G021650.1 | Solver.v1.11_VERG020210 | Soltu.DM1S1.11G021850 | AAA-type ATPase family protein | Peak SNP region |
| Soltu.DM.11G021660.1 | Solver.v1.11_VERG020220 | Soltu.DM1S1.11G021860 | NAD+ ADP-ribosyltransferases;NAD+ ADP-ribosyltransferases | Peak SNP region |
| Soltu.DM.11G021670.1 |  | Soltu.DM1S1.11G021870 | hypothetical protein | Peak SNP region |
| Soltu.DM.11G021680.1 | Solver.v1.11_VERG020230 | Soltu.DM1S1.11G021880 | alpha/beta-Hydrolases superfamily protein | Peak SNP region |
| Soltu.DM.11G021690.1 | Solver.v1.11_VERG020240 | Soltu.DM1S1.11G021890 | HXXXD-type acyl-transferase family protein | Peak SNP region |
| Soltu.DM.11G021700.1 | Solver.v1.11_VERG020250 | Soltu.DM1S1.11G021900 | myb-like HTH transcriptional regulator family protein | Peak SNP region |
| Soltu.DM.11G021710.1 | Solver.v1.11_VERG020260 | Soltu.DM1S1.11G021910 | HXXXD-type acyl-transferase family protein | Peak SNP region |
| Soltu.DM.11G021720.1 | Solver.v1.11_VERG020280 | Soltu.DM1S1.11G021920 | P-glycoprotein | Peak SNP region |
| Soltu.DM.11G021730.1 | Solver.v1.11_VERG020300 | Soltu.DM1S1.11G021930 | ATP binding cassette subfamily B4 | Peak SNP region |
| Soltu.DM.11G021740.1 | Solver.v1.11_VERG020310 | Soltu.DM1S1.11G021940 | sulfotransferase | Peak SNP region |
| Soltu.DM.11G021760.1 | Solver.v1.11_VERG020350 | Soltu.DM1S1.11G021970 | HXXXD-type acyl-transferase family protein | Peak SNP region |
| Soltu.DM.11G021790.3 | Solver.v1.11_VERG020360 | Soltu.DM1S1.11G022000 | F-box/RNI-like superfamily protein | Peak SNP region |
| Soltu.DM.11G021800.2 | Solver.v1.11_VERG020380 | Soltu.DM1S1.11G022010 | bZIP transcription factor family protein | Peak SNP region |
| Soltu.DM.11G021810.1 | Solver.v1.11_VERG020390 | Soltu.DM1S1.11G022020 | conserved hypothetical protein | Peak SNP region |
| Soltu.DM.11G021820.1 | Solver.v1.11_VERG020400 | Soltu.DM1S1.11G022030 | conserved hypothetical protein | Peak SNP region |
| Soltu.DM.11G021830.1 | Solver.v1.11_VERG020410 |  | ubiquinol-cytochrome C reductase UQCRX/QCR9-like family protein | Peak SNP region |
| Soltu.DM.11G021840.2 | Solver.v1.11_VERG020420 |  | Ribosomal protein L1p/L10e family | Peak SNP region |
| Soltu.DM.11G021850.1 |  | Soltu.DM1S1.11G022060 | ferredoxin | Peak SNP region |
| Soltu.DM.11G021860.1 | Solver.v1.11_VERG020430 | Soltu.DM1S1.11G022070 | Glycosyl hydrolase superfamily protein | Peak SNP region |
| Soltu.DM.11G021870.1 | Solver.v1.11_VERG020440 | Soltu.DM1S1.11G022080 | GCN5L1 family protein | Peak SNP region |
| Soltu.DM.11G021880.1 | Solver.v1.11_VERG020450 | Soltu.DM1S1.11G022090 | Calcium-binding EF-hand family protein | Peak SNP region |
| Soltu.DM.11G021890.2 | Solver.v1.11_VERG020460 | Soltu.DM1S1.11G022100 | PPR repeat family domain containing protein | Peak SNP region |
| Soltu.DM.11G021900.1 | Solver.v1.11_VERG020470 | Soltu.DM1S1.11G022110 | nuclear factor Y, subunit B11 | Peak SNP region |
| Soltu.DM.11G021910.1 | Solver.v1.11_VERG020480 | Soltu.DM1S1.11G022130 | sec23/sec24 transport family protein | Peak SNP region |
| Soltu.DM.11G021920.1 | Solver.v1.11_VERG020490 | Soltu.DM1S1.11G022140 | copper ion binding | Peak SNP region |
| Soltu.DM.11G021930.1 | Solver.v1.11_VERG020500 | Soltu.DM1S1.11G022150 | hypothetical protein | Peak SNP region |
| Soltu.DM.11G021940.1 | Solver.v1.11_VERG020510 | Soltu.DM1S1.11G022160 | Rho GTPase activating protein with PAK-box/P21-Rho-binding domain | Peak SNP region |
| Soltu.DM.11G021950.1 | Solver.v1.11_VERG020520 | Soltu.DM1S1.11G022170 | Protein of unknown function (DUF3049) | Peak SNP region |
| Soltu.DM.11G021960.1 | Solver.v1.11_VERG020530 | Soltu.DM1S1.11G022190 | nitrilase-like protein | Peak SNP region |
| Soltu.DM.11G021970.1 | Solver.v1.11_VERG020540 | Soltu.DM1S1.11G022200 | Late embryogenesis abundant (LEA) hydroxyproline-rich glycoprotein family | Peak SNP region |
| Soltu.DM.11G021980.1 | Solver.v1.11_VERG020550 |  | hypothetical protein | Peak SNP region |
| Soltu.DM.11G021990.1 | Solver.v1.11_VERG020560 | Soltu.DM1S1.11G022210 | germin-like protein | Peak SNP region |
| Soltu.DM.11G022000.1 | Solver.v1.11_VERG020570 | Soltu.DM1S1.11G022220 | germin-like protein subfamily 2 member 2 precursor | Peak SNP region |
| Soltu.DM.11G022010.1 | Solver.v1.11_VERG020580 | Soltu.DM1S1.11G022230 | germin-like protein | Peak SNP region |
| Soltu.DM.11G022020.1 | Solver.v1.11_VERG020590 | Soltu.DM1S1.11G022240 | germin-like protein | Peak SNP region |
| Soltu.DM.11G022030.1 | Solver.v1.11_VERG020600 | Soltu.DM1S1.11G022250 | Arp2/3 complex, 34 kD subunit p34-Arc | Peak SNP region |
| Soltu.DM.11G022040.1 | Solver.v1.11_VERG020610 | Soltu.DM1S1.11G022260 | NAC domain containing protein | Peak SNP region |
| Soltu.DM.11G022050.1 | Solver.v1.11_VERG020620 |  | hypothetical protein | Peak SNP region |
| Soltu.DM.11G022060.1 | Solver.v1.11_VERG020640 | Soltu.DM1S1.11G022270 | ARM repeat superfamily protein | Peak SNP region |
| Soltu.DM.11G022070.1 | Solver.v1.11_VERG020650 | Soltu.DM1S1.11G022280 | Protein of unknown function (DUF1022) | Peak SNP region |
| Soltu.DM.11G022080.1 | Solver.v1.11_VERG020670 | Soltu.DM1S1.11G022290 | OTU-like cysteine protease family protein | Peak SNP region |
| Soltu.DM.11G022090.1 | Solver.v1.11_VERG020680 | Soltu.DM1S1.11G022300 | conserved hypothetical protein | Peak SNP region |
| Soltu.DM.11G022100.1 | Solver.v1.11_VERG020690 | Soltu.DM1S1.11G022310 | conserved hypothetical protein | Peak SNP region |
| Soltu.DM.11G022110.1 | Solver.v1.11_VERG020700 | Soltu.DM1S1.11G022320 | Leucine-rich repeat (LRR) family protein | Peak SNP region |
| Soltu.DM.11G022120.1 | Solver.v1.11_VERG020710 | Soltu.DM1S1.11G022330 | F-box family protein | Peak SNP region |
| Soltu.DM.11G022130.1 | Solver.v1.11_VERG020720 | Soltu.DM1S1.11G022340 | conserved hypothetical protein | Peak SNP region |
| Soltu.DM.11G022140.1 | Solver.v1.11_VERG020730 | Soltu.DM1S1.11G022350 | nitrilase | Peak SNP region |
| Soltu.DM.11G022150.1 | Solver.v1.11_VERG020770 |  | methyl-CPG-binding domain | Peak SNP region |
| Soltu.DM.11G022160.1 | Solver.v1.11_VERG020810 | Soltu.DM1S1.11G022360 | NTM1-like |  |
| Soltu.DM.11G022170.1 | Solver.v1.11_VERG020820 | Soltu.DM1S1.11G022380 | conserved hypothetical protein |  |
| Soltu.DM.11G022180.1 | Solver.v1.11_VERG020830 | Soltu.DM1S1.11G022390 | Polynucleotidyl transferase, ribonuclease H-like superfamily protein |  |
| Soltu.DM.11G022190.1 | Solver.v1.11_VERG020840 | Soltu.DM1S1.11G022400 | ovate family protein |  |
| Soltu.DM.11G022200.1 | Solver.v1.11_VERG020850 | Soltu.DM1S1.11G022410 | Protein of unknown function (DUF1279) |  |
| Soltu.DM.11G022210.1 | Solver.v1.11_VERG020860 | Soltu.DM1S1.11G022420 | E2F transcription factor |  |
| Soltu.DM.11G022220.2 | Solver.v1.11_VERG020870 | Soltu.DM1S1.11G022430 | conserved hypothetical protein |  |
| Soltu.DM.11G022230.1 | Solver.v1.11_VERG020880 | Soltu.DM1S1.11G022440 | ribosomal protein large subunit |  |
| Soltu.DM.11G022240.1 | Solver.v1.11_VERG020890 | Soltu.DM1S1.11G022450 | uridine 5'-monophosphate synthase / UMP synthase (PYRE-F) (UMPS) |  |
| Soltu.DM.11G022250.1 | Solver.v1.11_VERG020900 | Soltu.DM1S1.11G022460 | Coiled-coil domain-containing protein 55 (DUF2040) |  |
| Soltu.DM.11G022260.1 | Solver.v1.11_VERG020910 | Soltu.DM1S1.11G022470 | Eukaryotic aspartyl protease family protein |  |
| Soltu.DM.11G022280.1 | Solver.v1.11_VERG020930 | Soltu.DM1S1.11G022510 | Late embryogenesis abundant (LEA) hydroxyproline-rich glycoprotein family |  |
| Soltu.DM.11G022290.2 | Solver.v1.11_VERG020940 | Soltu.DM1S1.11G022520 | methyl-CPG-binding domain |  |
| Soltu.DM.11G022300.1 | Solver.v1.11_VERG020950 | Soltu.DM1S1.11G022530 | plant U-box |  |
| Soltu.DM.11G022310.1 | Solver.v1.11_VERG020960 | Soltu.DM1S1.11G022540 | ThiF family protein |  |
| Soltu.DM.11G022320.1 | Solver.v1.11_VERG020980 | Soltu.DM1S1.11G022550 | BEL1-like homeodomain |  |
| Soltu.DM.11G022330.1 | Solver.v1.11_VERG020990 | Soltu.DM1S1.11G022560 | transcription factor-related |  |
| Soltu.DM.11G022340.1 | Solver.v1.11_VERG021000 | Soltu.DM1S1.11G022570 | Aluminium activated malate transporter family protein |  |
| Soltu.DM.11G022350.1 | Solver.v1.11_VERG021010 | Soltu.DM1S1.11G022580 | Mitochondrial import inner membrane translocase subunit Tim17/Tim22/Tim23 family protein | |
| Soltu.DM.11G022360.2 | Solver.v1.11_VERG021020 | Soltu.DM1S1.11G022590 | S-adenosyl-L-methionine-dependent methyltransferases superfamily protein |  |
| Soltu.DM.11G022370.1 | Solver.v1.11_VERG021030 | Soltu.DM1S1.11G022600 | TCP-1/cpn60 chaperonin family protein |  |
| Soltu.DM.11G022380.1 | Solver.v1.11_VERG021040 | Soltu.DM1S1.11G022610 | alpha-L-fucosidase |  |
| Soltu.DM.11G022390.2 | Solver.v1.11_VERG021050 | Soltu.DM1S1.11G022620 | NB-ARC domain-containing disease resistance protein |  |
| Soltu.DM.11G022400.1 | Solver.v1.11_VERG021060 | Soltu.DM1S1.11G022630 | myb domain protein |  |
| Soltu.DM.11G022410.1 | Solver.v1.11_VERG021070 | Soltu.DM1S1.11G022640 | lactoylglutathione lyase family protein / glyoxalase I family protein |  |
| Soltu.DM.11G022420.1 | Solver.v1.11_VERG021080 | Soltu.DM1S1.11G022650 | AMP-dependent synthetase and ligase family protein |  |
| Soltu.DM.11G022430.1 | Solver.v1.11_VERG021090 | Soltu.DM1S1.11G022660 | Heavy metal transport/detoxification superfamily protein |  |
| Soltu.DM.11G022440.1 | Solver.v1.11_VERG021100 | Soltu.DM1S1.11G022670 | Heavy metal transport/detoxification superfamily protein |  |
| Soltu.DM.11G022450.1 |  | Soltu.DM1S1.11G022680 | Ribosomal L18p/L5e family protein |  |
| Soltu.DM.11G022460.1 | Solver.v1.11_VERG021120 | Soltu.DM1S1.11G022690 | ABC transporter family protein |  |
| Soltu.DM.11G022470.1 | Solver.v1.11_VERG021130 |  | SAUR-like auxin-responsive protein family |  |
| Soltu.DM.11G022490.1 | Solver.v1.11_VERG021140 | Soltu.DM1S1.11G022710 | GTP binding Elongation factor Tu family protein |  |
| Soltu.DM.11G022500.1 | Solver.v1.11_VERG021150 | Soltu.DM1S1.11G022720 | conserved hypothetical protein |  |
| Soltu.DM.11G022510.1 |  | Soltu.DM1S1.11G022730 | F-box family protein |  |
| Soltu.DM.11G022520.1 | Solver.v1.11_VERG021160 | Soltu.DM1S1.11G022740 | F-box associated ubiquitination effector family protein |  |
| Soltu.DM.11G022530.1 | Solver.v1.11_VERG021170 | Soltu.DM1S1.11G022750 | 20S proteasome beta subunit G1 |  |
| Soltu.DM.11G022540.5 | Solver.v1.11_VERG021180 | Soltu.DM1S1.11G022760 | DNA-binding protein with MIZ/SP-RING zinc finger, PHD-finger and SAP domain |  |
| Soltu.DM.11G022550.2 | Solver.v1.11_VERG021190 | Soltu.DM1S1.11G022770 | Cytochrome B561, N terminal domain containing protein |  |
| Soltu.DM.11G022560.1 | Solver.v1.11_VERG021200 | Soltu.DM1S1.11G022780 | isovaleryl-CoA-dehydrogenase |  |
| Soltu.DM.11G022570.1 | Solver.v1.11_VERG021210 | Soltu.DM1S1.11G022790 | auxin response factor |  |
| Soltu.DM.11G022580.1 | Solver.v1.11_VERG021220 | Soltu.DM1S1.11G022800 | conserved hypothetical protein |  |
| Soltu.DM.11G022600.1 | Solver.v1.11_VERG021240 | Soltu.DM1S1.11G022820 | syntaxin of plants |  |
| Soltu.DM.11G022610.1 | Solver.v1.11_VERG021250 | Soltu.DM1S1.11G022830 | C2H2-like zinc finger protein |  |
| Soltu.DM.11G022620.1 | Solver.v1.11_VERG021260 |  | FASCICLIN-like arabinogalactan-protein |  |
| Soltu.DM.11G022630.1 | Solver.v1.11_VERG021270 | Soltu.DM1S1.11G022840 | ABC transporter family protein |  |
| Soltu.DM.11G022640.2 | Solver.v1.11_VERG021280 | Soltu.DM1S1.11G022850 | beta-galactosidase |  |
| Soltu.DM.11G022650.1 | Solver.v1.11_VERG021290 | Soltu.DM1S1.11G022860 | Late embryogenesis abundant (LEA) protein-related |  |
| Soltu.DM.11G022660.2 | Solver.v1.11_VERG021300 | Soltu.DM1S1.11G022870 | pyridoxine biosynthesis |  |
| Soltu.DM.11G022670.2 | Solver.v1.11_VERG021320 | Soltu.DM1S1.11G022880 | geminivirus rep interacting kinase |  |
| Soltu.DM.11G022680.1 | Solver.v1.11_VERG021330 | Soltu.DM1S1.11G022890 | Galactose oxidase/kelch repeat superfamily protein |  |
| Soltu.DM.11G022690.1 | Solver.v1.11_VERG021340 | Soltu.DM1S1.11G022900 | conserved hypothetical protein |  |
| Soltu.DM.11G022710.1 | Solver.v1.11_VERG021350 | Soltu.DM1S1.11G022910 | zinc finger (CCCH-type) family protein |  |
| Soltu.DM.11G022730.4 | Solver.v1.11_VERG021360 | Soltu.DM1S1.11G022920 | RING/U-box superfamily protein |  |
| Soltu.DM.11G022740.2 | Solver.v1.11_VERG021370 | Soltu.DM1S1.11G022940 | P-loop containing nucleoside triphosphate hydrolases superfamily protein |  |
| Soltu.DM.11G022750.1 | Solver.v1.11_VERG021380 | Soltu.DM1S1.11G022950 | Kiwellin |  |
| Soltu.DM.11G022780.1 | Solver.v1.11_VERG021410 | Soltu.DM1S1.11G023010 | Kiwellin |  |
| Soltu.DM.11G022790.1 | Solver.v1.11_VERG021420 | Soltu.DM1S1.11G023020 | Kiwellin |  |
| Soltu.DM.11G022800.1 | Solver.v1.11_VERG021430 | Soltu.DM1S1.11G023030 | POX (plant homeobox) family protein |  |
| Soltu.DM.11G022820.1 | Solver.v1.11_VERG021440 | Soltu.DM1S1.11G023040 | DNA-directed RNA polymerase, RBP11-like |  |
| Soltu.DM.11G022830.1 | Solver.v1.11_VERG021460 | Soltu.DM1S1.11G023050 | alpha carbonic anhydrase |  |
| Soltu.DM.11G022850.1 | Solver.v1.11_VERG021470 | Soltu.DM1S1.11G023070 | Protein kinase superfamily protein |  |
| Soltu.DM.11G022860.1 | Solver.v1.11_VERG021480 | Soltu.DM1S1.11G023090 | ATP-dependent helicase family protein |  |
| Soltu.DM.11G022870.1 | Solver.v1.11_VERG021490 | Soltu.DM1S1.11G023100 | Zinc finger (C3HC4-type RING finger) family protein |  |
| Soltu.DM.11G022880.1 | Solver.v1.11_VERG021500 | Soltu.DM1S1.11G023110 | Protein kinase superfamily protein |  |
| Soltu.DM.11G022890.1 | Solver.v1.11_VERG021510 | Soltu.DM1S1.11G023120 | cyclic nucleotide-gated channel |  |
| Soltu.DM.11G022900.1 | Solver.v1.11_VERG021520 |  | lysine decarboxylase family protein |  |
| Soltu.DM.11G022910.1 | Solver.v1.11_VERG021530 | Soltu.DM1S1.11G023130 | glycosyltransferase family protein |  |
| Soltu.DM.11G022920.1 | Solver.v1.11_VERG021540 | Soltu.DM1S1.11G023140 | Protein of unknown function (DUF707) |  |
| Soltu.DM.11G022930.1 | Solver.v1.11_VERG021550 | Soltu.DM1S1.11G023150 | Nucleotide/sugar transporter family protein |  |
| Soltu.DM.11G022940.1 | Solver.v1.11_VERG021560 | Soltu.DM1S1.11G023160 | Pleckstrin homology (PH) and lipid-binding START domains-containing protein |  |
| Soltu.DM.11G022950.1 | Solver.v1.11_VERG021570 | Soltu.DM1S1.11G023170 | tyrosylprotein sulfotransferase |  |
| Soltu.DM.11G022960.1 | Solver.v1.11_VERG021580 | Soltu.DM1S1.11G023180 | GATA transcription factor |  |
| Soltu.DM.11G022970.2 | Solver.v1.11_VERG021590 | Soltu.DM1S1.11G023190 | auxin response factor |  |
| Soltu.DM.11G022980.1 | Solver.v1.11_VERG021600 | Soltu.DM1S1.11G023200 | SIT4 phosphatase-associated family protein |  |
| Soltu.DM.11G022990.1 | Solver.v1.11_VERG021610 | Soltu.DM1S1.11G023210 | conserved hypothetical protein |  |
| Soltu.DM.11G023000.1 | Solver.v1.11_VERG021620 | Soltu.DM1S1.11G023220 | Homeobox-leucine zipper family protein / lipid-binding START domain-containing protein | |
| Soltu.DM.11G023010.1 | Solver.v1.11_VERG021630 | Soltu.DM1S1.11G023230 | dsRNA-binding protein |  |
| Soltu.DM.11G023020.1 | Solver.v1.11_VERG021640 | Soltu.DM1S1.11G023240 | Protein of unknown function, DUF584 |  |
| Soltu.DM.11G023030.1 | Solver.v1.11_VERG021650 |  | Ribosomal protein L11 family protein |  |
| Soltu.DM.11G023040.1 | Solver.v1.11_VERG021660 | Soltu.DM1S1.11G023260 | plasma membrane intrinsic protein |  |
| Soltu.DM.11G023050.1 | Solver.v1.11_VERG021680 | Soltu.DM1S1.11G023280 | conserved hypothetical protein |  |
| Soltu.DM.11G023060.1 | Solver.v1.11_VERG021670 | Soltu.DM1S1.11G023270 | conserved hypothetical protein |  |
| Soltu.DM.11G023070.1 | Solver.v1.11_VERG021690 | Soltu.DM1S1.11G023290 | PDI-like 1-4 |  |
| Soltu.DM.11G023090.1 | Solver.v1.11_VERG021700 | Soltu.DM1S1.11G023300 | 4-hydroxy-3-methylbut-2-enyl diphosphate synthase |  |
| Soltu.DM.11G023100.1 | Solver.v1.11_VERG021710 | Soltu.DM1S1.11G023310 | Glutaredoxin family protein |  |
| Soltu.DM.11G023110.1 | Solver.v1.11_VERG021720 | Soltu.DM1S1.11G023320 | TRF-like |  |
| Soltu.DM.11G023120.1 | Solver.v1.11_VERG021730 | Soltu.DM1S1.11G023330 | Protein of unknown function, DUF547 |  |
| Soltu.DM.11G023130.1 | Solver.v1.11_VERG021740 | Soltu.DM1S1.11G023340 | P-loop containing nucleoside triphosphate hydrolases superfamily protein |  |
| Soltu.DM.11G023140.1 | Solver.v1.11_VERG021750 | Soltu.DM1S1.11G023350 | ABC transporter family protein |  |
| Soltu.DM.11G023150.1 | Solver.v1.11_VERG021760 |  | AGAMOUS-like |  |
| Soltu.DM.11G023170.1 | Solver.v1.11_VERG021770 | Soltu.DM1S1.11G023360 | Cysteine proteinases superfamily protein |  |
| Soltu.DM.11G023180.1 | Solver.v1.11_VERG021780 | Soltu.DM1S1.11G023370 | allene oxide synthase |  |
| Soltu.DM.11G023190.1 | Solver.v1.11_VERG021790 | Soltu.DM1S1.11G023380 | chaperonin-60alpha |  |
| Soltu.DM.11G023200.1 | Solver.v1.11_VERG021800 | Soltu.DM1S1.11G023390 | P-loop containing nucleoside triphosphate hydrolases superfamily protein |  |
| Soltu.DM.11G023210.2 | Solver.v1.11_VERG021830 | Soltu.DM1S1.11G023400 | 5'-AMP-activated protein kinase beta-2 subunit protein |  |
| Soltu.DM.11G023220.1 | Solver.v1.11_VERG021840 | Soltu.DM1S1.11G023410 | nitrate transporter 2.4 |  |
| Soltu.DM.11G023230.1 |  | Soltu.DM1S1.11G023420 | Cytochrome P450 superfamily protein |  |
| Soltu.DM.11G023240.1 | Solver.v1.11_VERG021850 | Soltu.DM1S1.11G023430 | nitrate transporter 2.4 |  |
| Soltu.DM.11G023250.1 | Solver.v1.11_VERG021860 | Soltu.DM1S1.11G023440 | nitrate transporter 2.4 |  |
| Soltu.DM.11G023280.1 | Solver.v1.11_VERG021880 | Soltu.DM1S1.11G023460 | ABC-2 type transporter family protein |  |
| Soltu.DM.11G023290.1 | Solver.v1.11_VERG021890 | Soltu.DM1S1.11G023470 | GTP binding Elongation factor Tu family protein |  |
| Soltu.DM.11G023300.1 | Solver.v1.11_VERG021900 | Soltu.DM1S1.11G023480 | PDI-like 5-1 |  |
| Soltu.DM.11G023310.1 |  | Soltu.DM1S1.11G023490 | HXXXD-type acyl-transferase family protein |  |
| Soltu.DM.11G023330.1 | Solver.v1.11_VERG021930 |  | hypothetical protein |  |
| Soltu.DM.11G023370.1 | Solver.v1.11_VERG021910 | Soltu.DM1S1.11G023510 | HXXXD-type acyl-transferase family protein |  |
| Soltu.DM.11G023420.1 |  | Soltu.DM1S1.11G023550 | NB-ARC domain-containing disease resistance protein |  |
| Soltu.DM.11G023430.1 |  | Soltu.DM1S1.11G023560 | hypothetical protein |  |
| Soltu.DM.11G023500.1 |  | Soltu.DM1S1.11G023600 | Thioredoxin superfamily protein |  |
| Soltu.DM.11G023510.2 | Solver.v1.11_VERG021970 | Soltu.DM1S1.11G023610 | FTSH protease |  |

Table S10. Syntenic genes within the 1.5-LOD supported interval in the chromosome 11 QTL for *ui11.1*.

| Gene name | Chromosome | Start position | End position | Log_2_ fold change between MSJJ1821F2-041 and DM1S1 | Log_2_ fold change between MSJJ1821F2-049 and DM1S1 | Log_2_ fold change between MSJJ1821F2-091 and DM1S1 | Log_2_ fold change between MSJJ1821F2-041 and MSII1813-2 | Log_2_ fold change between MSJJ1821F2-049 and MSII1813-2 | Log_2_ fold change between MSJJ1821F2-091 and MSII1813-2 | Annotation |
| --- | --- | --- | --- | --- | --- | --- | --- | --- | --- | --- |
| Solver.v1.01_VERG009090 | 1 | 13321393 | 13328170 | 0.545731 | 0.507910 | 0.398132 | 0.350004 | 0.176543 | 0.200804 | VH1-interacting kinase |
| Solver.v1.01_VERG042990 | 1 | 33839524 | 33840302 | -19.054800 | -20.801200 | -0.661030 | - | - | 20.351040 | Ribonuclease T2 family protein (S-RNase) |
| Solver.v1.01_VERG044150 | 1 | 37563006 | 37565956 | -1.347443 | -0.249594 | -0.163998 | -0.898047 | 0.073518 | 0.296377 | conserved hypothetical protein |
| Solver.v1.01_VERG059340 | 1 | 45781848 | 45783136 | -4.537804 | -1.551699 | -0.731577 | -2.858067 | -0.014939 | 0.947089 | F-box/RNI-like superfamily protein |
| Solver.v1.01_VERG062380 | 1 | 56717779 | 56728824 | -0.624119 | 0.260545 | -0.044132 | -0.337344 | 0.410400 | 0.239842 | VERG062380WD40/YVTN repeat-like-containing domain;Bromodomain |
| Solver.v1.01_VERG062390 | 1 | 56734424 | 56735221 | -0.500509 | -0.331591 | -0.153598 | 0.165281 | 0.199091 | 0.510775 | zinc knuckle (CCHC-type) family protein |
| Solver.v1.01_VERG062410 | 1 | 56761483 | 56764828 | -2.834547 | -1.147423 | 0.339529 | 0.001008 | 1.550951 | 3.159267 | Mitochondrial import inner membrane translocase subunit Tim17/Tim22/Tim23 family protein |
| Solver.v1.01_VERG062420 | 1 | 56774817 | 56788597 | -0.729569 | 0.062786 | -0.102210 | -0.356069 | 0.299992 | 0.268933 | binding |
| Solver.v1.01_VERG062520 | 1 | 57120757 | 57122886 | -1.286057 | -0.115800 | -0.209697 | -0.836545 | 0.198919 | 0.238401 | pentatricopeptide (PPR) domain protein |
| Solver.v1.01_VERG062840 | 1 | 58169896 | 58175363 | -0.690812 | 0.083085 | 0.019196 | -0.333994 | 0.304736 | 0.373893 | pumilio |
| Solver.v1.01_VERG062960 | 1 | 58762898 | 58764385 | -3.499334 | -1.314440 | -0.403507 | -0.870439 | 1.192743 | 2.235362 | Telomere-capping, CST complex subunit domain containing protein |
| Solver.v1.01_VERG063050 | 1 | 58895710 | 58901349 | -4.537804 | -1.931223 | -0.731577 | -1.911234 | 0.522581 | 1.847278 | RNI-like superfamily protein |
| Solver.v1.01_VERG064250 | 1 | 61662765 | 61663082 | -4.608487 | -0.854738 | -1.636914 | -2.816186 | 0.798071 | 0.155453 | flowering promoting factor |
| Solver.v1.01_VERG064540 | 1 | 62078299 | 62087931 | -7.140802 | -2.778324 | -0.314840 | -2.909039 | 1.282696 | 3.905523 | cullin |
| Solver.v1.01_VERG064840 | 1 | 62522644 | 62526906 | 0.568543 | 0.780703 | 0.361490 | -0.095599 | -0.020855 | -0.305207 | GTP1/OBG family protein |
| Solver.v1.11_VERG018200 | 11 | 36056000 | 36066786 | 2.213157 | -1.098187 | -0.590854 | -0.418647 | -3.879383 | -3.228054 | Late embryogenesis abundant protein (LEA) family protein |

Table S11. List of significantly differentially expressed genes (α = 0.01) in the styles of F2 progeny and parents used in the RNA-seq and differential expression analysis, corresponding to the observed phenotypic classes for interspecific compatibility.

Figure S8. Heatmap of Log_2_ fold change of *ui11.1* candidate gene Soltu.DM.11G021610.1 in the chromosome 11 QTL. Comparisons show similar expression in MSJJ1821F2-049 and MSII1813-2 which lack IRBs. While DM1S1 and MSJJ1821F2-041 with functional IRBs are similar and having significantly lower expression. MSJJ1821F2-091 also has functional IRBs, and has an intermediate expression of Soltu.DM.11G021610.1 but more similar to DM1S1. Based on SNP data, MSJJ1821F2-041 is homozygous for the DM1S1 allele, MSJJ1821F2-049 is homozygous for the MSII1813-2 allele, and MSJJ1821F2-091 is heterozygous.

Solver.v1.11_VERG020170.1 MELTSVLNFLENRTILVTGATGFLAKIFVEKILRVQPNVKKLYLLLRAADDKSAMQRFNN 60

Soltu.DM.11G021610.1 MELTSVLNFLENRTILVTGATGFLAKIFVEKILRVQPNVKKLYLLLRAADDKSAMQRFNN 60

Soltu.DM1S1.11G021810.1 MELTSVLNFLENRTILVTGATGFLAKIFVEKILRVQPNVKKLYLLLRAADDKSAMQRFNN 60

************************************************************

Solver.v1.11_VERG020170.1 EVVGKDLFKVLREKCGPNFTTFVSQRTTIVPGDITCDNLGVNDTNLLEQMWKEVDIVVNL 120

Soltu.DM.11G021610.1 EVVGKDLFKVLREKCGPNFTTFVSQRTTIVPGDITCENLGVNDTNLLEQMWKEVDIVVNL 120

Soltu.DM1S1.11G021810.1 EVVGKDLFKVLREKCGPNFTTFVSQRTTIVPGDITCENLGVNDTNLLEQMWKEVDIVVNL 120

************************************:***********************

Solver.v1.11_VERG020170.1 AATTNFDERYDVALGLNTFGARHVLNFAKKCNKLKVLLHVSTAYVCGEKEGLMLEKPYYM 180

Soltu.DM.11G021610.1 AATTNFDERYDVALGLNTFGARHVLNFAKKCNKLKVLLHVSTAYVCGEKEGLMLEKPYYM 180

Soltu.DM1S1.11G021810.1 AATTNFDERYDVALGLNTFGARHVLNFAKKCNKLKVLLHVSTAYVCGEKEGLMLEKPYYM 180

************************************************************

Solver.v1.11_VERG020170.1 GETLNGTLGLDIEVEKKVMDEKLKQLNAENASEKSITTAMKELGLERARKYGWPNTYVFT 240

Soltu.DM.11G021610.1 GETLNGTLGLDIEAEKKVMDEKLKQLNAENASEKSITTTMKELGLERARKYGWPNTYVFT 240

Soltu.DM1S1.11G021810.1 GETLNGTLGLDIEAEKKVMDEKLKQLNAENASEKSITTAMKELGLERARKYGWPNTYVFT 240

*************.************************:*********************

Solver.v1.11_VERG020170.1 KAMGEMLLGKLKEEVPLVINRPTIITSTFKEPFPGWVEGIRTIDSLAVGYGKGRITCFLG 300

Soltu.DM.11G021610.1 KAMGEMLLGKLKEEVPLVINRPTIITSTFKEPFPGWVEGIRTIDSLAVGYGKGRITCFLG 300

Soltu.DM1S1.11G021810.1 KAMGEMLLGKLKEEVPLVINRPTIITSTFKEPFPGWVEGIRTIDSLAVGYGKGRITCFLG 300

************************************************************

Solver.v1.11_VERG020170.1 NPKTILDVIPADMVVNSMIVAMMAHADQKGSETIYQIGSSVSNPLNITNLRDYGFNYFRK 360

Soltu.DM.11G021610.1 NPKTILDVIPADMVVNSMIVAMMAHADQKGSETIYQIGSSVSNPLNITNLRDYGFNYFRK 360

Soltu.DM1S1.11G021810.1 NPKTILDVIPADMVVNSMIVAMMAHADQKGSETIYQIGSSVSNPLNITNLRDYGFNYFRK 360

************************************************************

Solver.v1.11_VERG020170.1 NPWINKVDGKPIIVGKVNVLSSMDSFQRYMALHYMLPLKGLEIVNAAFCQYFQGKYLELY 420

Soltu.DM.11G021610.1 NPWINKVDGKPIIVGKVNVLSSMDSFQRYMALHYMLPLKGLEIVNAAFCQYFQGKYLELY 420

Soltu.DM1S1.11G021810.1 NPWINKVDGKPIIVGKVNVLSSMDSFQRYMALHYMLPLKGLEIVNAAFCQYFQGKYLELY 420

************************************************************

Solver.v1.11_VERG020170.1 RKIKFVMRLIDLYGPYLFLKAAFDDLNTEKLRMAAKESGIEPEIFYFDPKIINWEDYFMK 480

Soltu.DM.11G021610.1 RKIKFVMRLIDLYGPYLFLKAAFDDLNTEKLRMAAKESGIEPEIFYFDPKIINWEDYFMK 480

Soltu.DM1S1.11G021810.1 RKIKFVMRLIDLYGPYLFLKAAFDDLNTEKLRMAAKESGIEPEIFYFDPKIINWEDYFMK 480

************************************************************

Solver.v1.11_VERG020170.1 IHLPGVVRYVFK 492

Soltu.DM.11G021610.1 IHLPGVVRYVFK 492

Soltu.DM1S1.11G021810.1 IHLPGVVRYVFK 492

************

Figure S9. Multiple sequence alignment of Soltu.DM.11G021610 and the DM1S1 and MSII1813-2 orthologs Soltu.DM1S1.11G021810 and Solver.v1.11_VERG020170, respectively. Generated using CLUSTAL O(1.2.4) multiple sequence alignment.
