# Supplementary figures and images for: Genetic basis for broad interspecific compatibility in Solanum verrucosum
Source: Plant J. 2025 Aug 21;123(4):e70426. doi: 10.1111/tpj.70426 (PMC12368477; doi:10.1111/tpj.70426)

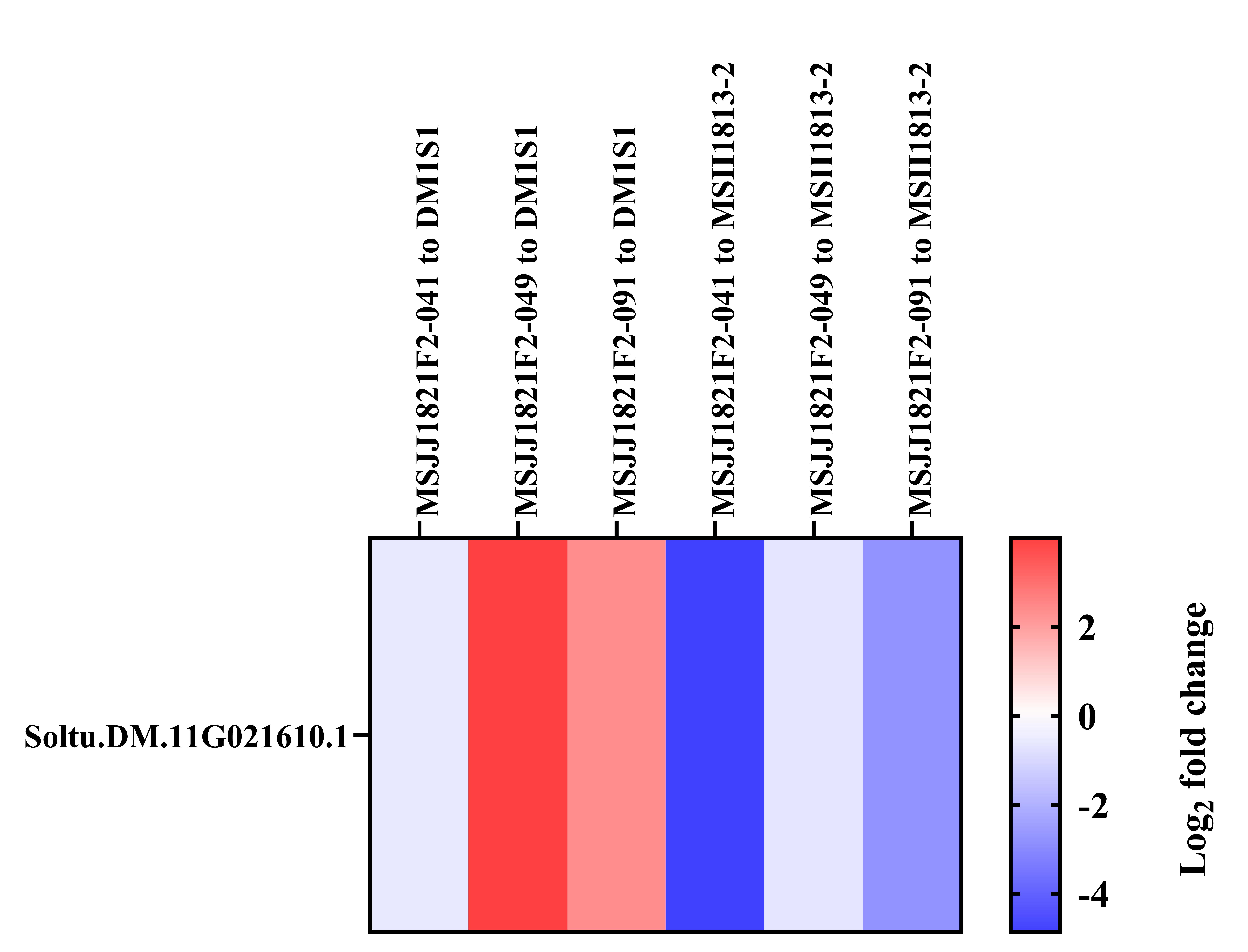

Supplement: Supplementary file 2 — Figure S1. Behavior of S. pinnatisectum pollen tubes in the styles of the parents, F1, and F2 progeny. Figure S2. Phylogeny of S‐RNase CDS sequences from parents of the mapping population. Figure S3. Phylogeny of SLF sequences, showing the relationship of parental SLF sequences used in the mapping population. Figure S4. Mosaic plots showing the distribution of QTL haplotypes across phenotypic classes. Figure S5. Multiple sequence alignment of Soltu.DM.03G036770.1 and the DM1S1 and MSII1813‐2 orthologs. Figure S6. Gene tree of pectinacetylesterases from S. verrucosum MSII1813‐2, S. chacoense M6, S. tuberosum DM, S. tuberosum DM1S1, S. tuberosum RH, and Solanum lycopersicum M82 that was used as an outgroup. Figure S7. Multiple sequence alignment of Soltu.DM.09G000840.1 and the DM1S1 and MSII1813‐2 orthologs. Figure S8. Heat map of Log2 fold change of ui11.1 candidate gene Soltu.DM.11G021610.1 in the chromosome 11 QTL. Figure S9. Multiple sequence alignment of Soltu.DM.11G021610 and the DM1S1 and MSII1813‐2 orthologs. Table S1. Pollination phenotyping data for the F2 mapping population. Table S2. List of significant SNPs, their physical and map positions, LOD values, and nearest annotation on DM v6.1 assembly. Table S3. S‐RNase and SLF sequences from parents used in the mapping population used in the construction of phylogenies. Table S4. S‐RNase and SLF sequences used as comparison for the construction of phylogenies. Table S5. Transcripts per million (TPM) values for pollen expressed Solver.v1.03_VERG035240.1. Table S6. Genome assembly metrics for S. verrucosum MSII1813‐2. Table S7. Benchmarking universal single copy orthologs in the S. verrucosum MSII1813‐2 genome sequence and annotation. Table S8. Repetitive sequences identified in S. verrucosum MSII1813‐2. Table S9. Protein coding genes annotated in S. verrucosum MSII1813‐2. Table S10. Syntenic genes within the chromosome 11 QTL for ui11.1. Table S11. List of significantly differentially expressed genes (α = 0.01) [file TPJ-123-0-s002.zip › Figure S8.jpg]
